# Comparative efficacy of dietary interventions for overweight or obese adults with type 2 diabetes: a systematic review and network meta-analysis of randomized controlled trials

**DOI:** 10.3389/fnut.2026.1853598

**Published:** 2026-07-15

**Authors:** Ranjun Xu, Yuru Chang, Enhui Su, Yongheng Wang, Yifan Zhang, Xueying Ma, Xue Han, Ruifang Zhu

**Affiliations:** 1 College of Nursing, Shanxi Medical University, Taiyuan, Shanxi Province, China; 2 The First Hospital of Shanxi Medical University, Taiyuan, Shanxi Province, China

**Keywords:** dietary patterns, network meta-analysis, obese, overweight, type 2 diabetes

## Abstract

**Background:**

Dietary intervention is a cornerstone of management for patients with type 2 diabetes who are overweight or obese; however, the optimal strategy remains uncertain. We therefore conducted a network meta-analysis to compare the effects of time-restricted eating (TRE), short-term fasting (STF), and continuous energy restriction (CER) vs. general diet (GD) on glycemic, anthropometric, and lipid-related outcomes.

**Methods:**

PubMed, Embase, Web of Science, and the Cochrane Library were systematically searched for randomized controlled trials published through 15 February 2026. Eligible studies enrolled overweight or obese adults with type 2 diabetes undergoing TRE, STF, or CER, with GD as the comparator. Risk of bias was assessed using the Cochrane Risk of Bias 2.0 tool. A frequentist random-effects network meta-analysis was conducted using Stata 17.0 (MP), with interventions ranked by the surface under the cumulative ranking curve (SUCRA). Univariable network meta-regression was used to explore potential effect modifiers. Pairwise random-effects meta-analyses were performed as supportive analyses for available direct comparisons. Evidence certainty was evaluated using the CINeMA framework.

**Results:**

We included 13 RCTs involving 805 participants. In the network meta-analysis, TRE was associated with a significant reduction in body weight (MD = −2.32 kg, 95% CI: −2.99 to −1.65), BMI (MD = −1.32 kg/m^2^, 95% CI: −1.71 to −0.92), waist circumference (MD = −3.69 cm, 95% CI: −5.07 to −2.30), and fasting blood glucose (MD = −0.85 mmol/L, 95% CI: −1.24 to −0.47). Compared with GD, TRE significantly reduced HbA1c (MD = −0.55, 95% CI: −0.77 to −0.33) and total cholesterol (MD = −0.17 mmol/L, 95% CI: −0.19 to −0.14). CER showed similar favorable effects on HbA1c, body weight, BMI, and waist circumference. According to SUCRA and treatment probability rankings, TRE consistently ranked first for multiple outcomes. The evidence for STF remained insufficient to draw definitive conclusions.

**Conclusion:**

TRE was associated with favorable effects on glycemic control, weight reduction, and waist circumference, and it ranked highly across multiple outcomes. However, direct comparisons between TRE and CER were generally not statistically significant. Therefore, TRE may be considered a promising dietary strategy, while CER also remains an effective option for selected metabolic outcomes. In practice, dietary strategies should be individualized according to the patient’s clinical profile and treatment needs.

**Systematic review registration:**

PROSPERO, identifier (CRD420261339818).

## 1 Introduction

Type 2 diabetes mellitus (T2DM) is a chronic metabolic disorder characterized by insulin resistance and relative insulin deficiency. Its development is closely associated with obesity; approximately 80–90% of individuals with T2DM are overweight or obese (1). In patients with T2DM and excess adiposity, achieving glycemic control can be difficult. Adipose tissue expansion is accompanied by increased secretion of proinflammatory cytokines, which impair insulin signaling. As obesity progresses, systemic insulin resistance and disordered glucose metabolism worsen further, thereby accelerating disease progression (2, 3). With the rising prevalence of overweight and obesity, the economic burden of T2DM is also expected to increase substantially (4), with global costs projected to reach approximately $2.5 trillion by 2030 (5).

Glucagon-like peptide-1 receptor agonists, such as liraglutide, are widely used in clinical practice for the treatment of obesity in patients with T2DM. These agents reduce body weight and improve metabolic control (6, 7). However, they are frequently associated with gastrointestinal adverse effects, with reported rates as high as 72.5% (7, 8), and require subcutaneous administration, which may compromise adherence (9). Although metformin has been recommended as the first-line glucose-lowering therapy, its effect on weight reduction is limited, and its use is often restricted by gastrointestinal intolerance in some patients (10, 11).

More recently, a range of nutritional strategies for individuals with overweight/obesity and T2DM has attracted considerable interest because of their metabolic plausibility and clinical feasibility (12, 13). Time-restricted eating (TRE) and short-term fasting (STF) may reduce hepatic lipid accumulation by promoting glycogen depletion during fasting, accelerating fatty acid oxidation, and increasing ketogenesis (14). In addition, TRE may help restore circadian alignment between melatonin and insulin secretion, thereby potentially easing the metabolic burden imposed on pancreatic *β*-cells by late-night food intake (15). In contrast, continuous energy restriction (CER) appears to exert its metabolic effects by reducing the flux of free fatty acids to the liver and improving insulin signaling through enhanced phosphorylation cascades (16).

There is substantial evidence for dietary strategies to manage overweight or obesity in patients with T2DM; however, the comparative effectiveness of these approaches remains unclear. Accordingly, this network meta-analysis compared the relative efficacy of TRE, STF, and CER against general diet (GD) in overweight or obese adults with type 2 diabetes. Previous systematic reviews have often overlooked both the certainty of the evidence and the clinical relevance of the observed Minimal Important Differences (MIDs). To address this gap, this study employs a Network Meta-Analysis (NMA) and applies both the GRADE and CINeMA frameworks to enhance confidence in the findings. By anchoring the analysis to MID-based evaluations, we move beyond statistical significance to identify which dietary interventions produce clinically meaningful health benefits, thereby providing more targeted dietary guidance.

## 2 Methods

### 2.1 Materials and methods

We performed the network meta-analysis in accordance with the PRISMA-NMA extension statement for systematic reviews and meta-analyses (Supplementary Table 1) (17). To ensure transparency, methodological rigor, and novelty, the protocol was prospectively registered in the International Prospective Register of Systematic Reviews (PROSPERO; CRD420261339818).

### 2.2 Data sources and search strategy

We conducted a systematic literature search of PubMed, Embase, the Cochrane Library, and Web of Science from inception through 15 February 2026. The search strategy incorporated Medical Subject Headings (MeSH), Emtree, and relevant keywords pertaining to “obesity,” “overweight,” “randomized clinical trials,” “type 2 diabetes mellitus,” and various fasting regimens, such as “intermittent fasting,” “caloric restriction,” and “time-restricted eating” (Supplementary Table 2). No language restrictions were applied to ensure a comprehensive synthesis of the available evidence.

### 2.3 Selection criteria

Inclusion criteria:

1. Adult patients with a clinically confirmed diagnosis of T2DM who are obese or overweight (defined as a BMI of ≥25 kg/m^2^).
2. The experimental group adopted one of the following three dietary intervention models (18):

Continuous energy restriction (CER): Daily energy intake is reduced by 20–30% compared to daily energy requirements.

Short-term fasting (STF): On two to three consecutive or non-consecutive days each week, daily calorie intake is limited to approximately 25% of the target requirement.

Time-restricted eating (TRE): This approach involves limiting daily eating to less than 12 h and fasting during the remaining hours.

1. Control group: Head-to-head comparative studies of the three dietary patterns described above, or randomized controlled trials (RCTs) comparing these dietary patterns with a general diet (GD), standard diabetes dietary management, or guideline-recommended dietary regimens. In this study, comparator interventions were harmonized and grouped as GD when they represented usual care, standard dietary advice, or guideline-based dietary management.
2. Randomized controlled trials should report at least one of the following outcome measures:

Glycated hemoglobin (HbA1c), fasting blood glucose (FBG), weight, BMI, waist circumference (WC), and total cholesterol (TC).

Exclusion criteria:

1. Randomized controlled trials conducted at different stages within the same cohort of patients.
2. Randomized controlled trials with unclear outcome measures.
3. Reviews or case reports.

Before inclusion in the review, studies were screened by title and abstract by two independent reviewers. Subsequently, the full texts of potentially eligible studies were assessed, and data extraction was performed independently by two reviewers to ensure accuracy and minimize bias. Any discrepancies were resolved through discussion or consultation with a third reviewer.

### 2.4 Data extraction

Two reviewers independently extracted data in accordance with PRISMA guidelines. We resolved disagreements through discussion or, when necessary, by consulting a third reviewer. We recorded the following information for each study: lead author, year of publication, study site, sample size, and baseline patient characteristics (including mean age, BMI, and HbA1c). We also detailed the treatment protocols for each intervention arm. For continuous outcomes, we preferentially extracted change-from-baseline means and standard deviations (SDs) when they were reported by the original studies. When change scores were not reported, the mean change was calculated as the difference between post-intervention and baseline values. The corresponding SD of the change score was estimated using the standard formula:


$$\begin{array}{l} \text{SDchange}\\ =\sqrt{\text{Baseline}\;{\mathrm{SD}}^{2}+\text{Endpoint}\;{\mathrm{SD}}^{2}-2R\cdot \text{Baseline}\;\mathrm{SD}\cdot \text{Endpoint}\;\mathrm{SD}} \end{array}$$

where R denotes the within-person correlation between baseline and follow-up measurements. When R was not reported, it was imputed from the most complete eligible study reporting both baseline and follow-up data for the same outcome and applied consistently within that outcome (19). This approach was used to standardize outcome reporting across trials and to maximize comparability in the network meta-analysis.

### 2.5 Quality assessment

We assessed the methodological quality using the updated Cochrane Risk of Bias (RoB 2) tool. Potential sources of bias were evaluated across five predefined domains: the randomization process, deviations from intended interventions, missing outcome data, measurement of outcomes, and selection of the reported results. For each domain and overall study quality, risk of bias was categorized as low, some concerns, or high (20).

### 2.6 Statistical analysis

We conducted a frequentist random-effects network meta-analysis using Stata 17.0. For outcomes reported on the same scale and in identical units, treatment effects were expressed as mean differences (MDs) with 95% confidence intervals (CIs). When outcomes were measured using different instruments, standardized mean differences (SMDs) were calculated. We applied a random-effects model throughout, with between-study variance (τ^2^) estimated using restricted maximum-likelihood. Multi-arm trials were incorporated using the augment command to account for correlations between comparisons within the same study and to avoid double-counting of shared control groups. We assessed consistency in networks with closed loops. Global inconsistency was examined using the design-by-treatment interaction model, and local inconsistency was evaluated through node-splitting analyses. Inconsistency factors (IFs) and their 95% CIs were calculated for each loop; intervals that included zero were interpreted as indicating no significant disagreement between the direct and indirect evidence. A two-sided *p*-value < 0.05 was considered statistically significant. Network plots were generated to illustrate the structure of the evidence. Node size reflected the total sample size for each intervention, and edge thickness corresponded to the number of direct comparisons. We ranked treatments using the surface under the cumulative ranking curve (SUCRA), the probability of being the best treatment, and mean ranks. To provide results more directly interpretable in clinical practice, conventional pairwise meta-analyses were also performed for head-to-head comparisons between TRE, CER, and GD when direct evidence was available. These analyses used the same effect measures and random-effects model, as described above. Forest plots were generated to present pooled estimates for each direct comparison. We constructed comparison-adjusted funnel plots when at least 10 studies were available to explore potential small-study effects. We performed sensitivity analyses using a leave-one-out approach, sequentially excluding each study to assess the stability of the results. We conducted univariate network meta-regression analyses to examine the influence of prespecified study-level covariates. Regression coefficients, 95% CIs, and Wald test *p* values were reported.

### 2.7 GRADE grading

We evaluated the certainty of evidence from the NMA using the CINeMA framework, which operationalizes the GRADE approach for NMA. As all included studies were randomized controlled trials, the initial certainty rating was high. Six domains were assessed: within-study bias, indirectness, imprecision, heterogeneity, incoherence, and reporting bias (including publication bias and small-study effects). Risk of bias was evaluated using the ROB 2.0 tool. Domain-level judgments were incorporated into CINeMA and weighted against each study’s contribution to the corresponding network estimate at the comparison level. Indirectness was assessed in terms of transitivity and exchangeability. We examined potential effect modifiers identified *a priori*—such as baseline disease severity, intervention intensity, and duration of follow-up—and evaluated the comparability of direct and indirect evidence with respect to population characteristics, intervention modalities, comparators, and outcome definitions and metrics. Imprecision was judged against prespecified minimal clinically important differences (MCIDs). The MCID thresholds were defined as follows: 0.5% for HbA1c; 0.5 mmol/L for FBG; 2 kg for body weight; 1 kg/m^2^ for BMI; 3 cm for WC; and 0.38 mmol/L for TC (21–26). For each outcome, we examined whether the 95% confidence interval (CI) crossed the line of no effect and whether it lay entirely beyond the corresponding MCID threshold. Heterogeneity was primarily evaluated using the between-study variance (τ^2^) derived from the random-effects model, together with the extent to which the CI overlapped the MCID boundaries. Incoherence within closed loops was assessed using node-splitting methods implemented in CINeMA, complemented by the SIDE approach and the design-by-treatment interaction model. Reporting bias was explored through searches of clinical trial registries and gray literature, as well as visual inspection of funnel plots to assess potential small-study effects. Each domain was rated as having no concerns, some concerns, or major concerns. In accordance with GRADE guidance, evidence was downgraded by one level for serious concerns and by two levels for very serious concerns. The overall certainty of evidence was ultimately categorized as high, moderate, low, or very low.

## 3 Result

### 3.1 Literature review and study characteristics

**Figure 1:**
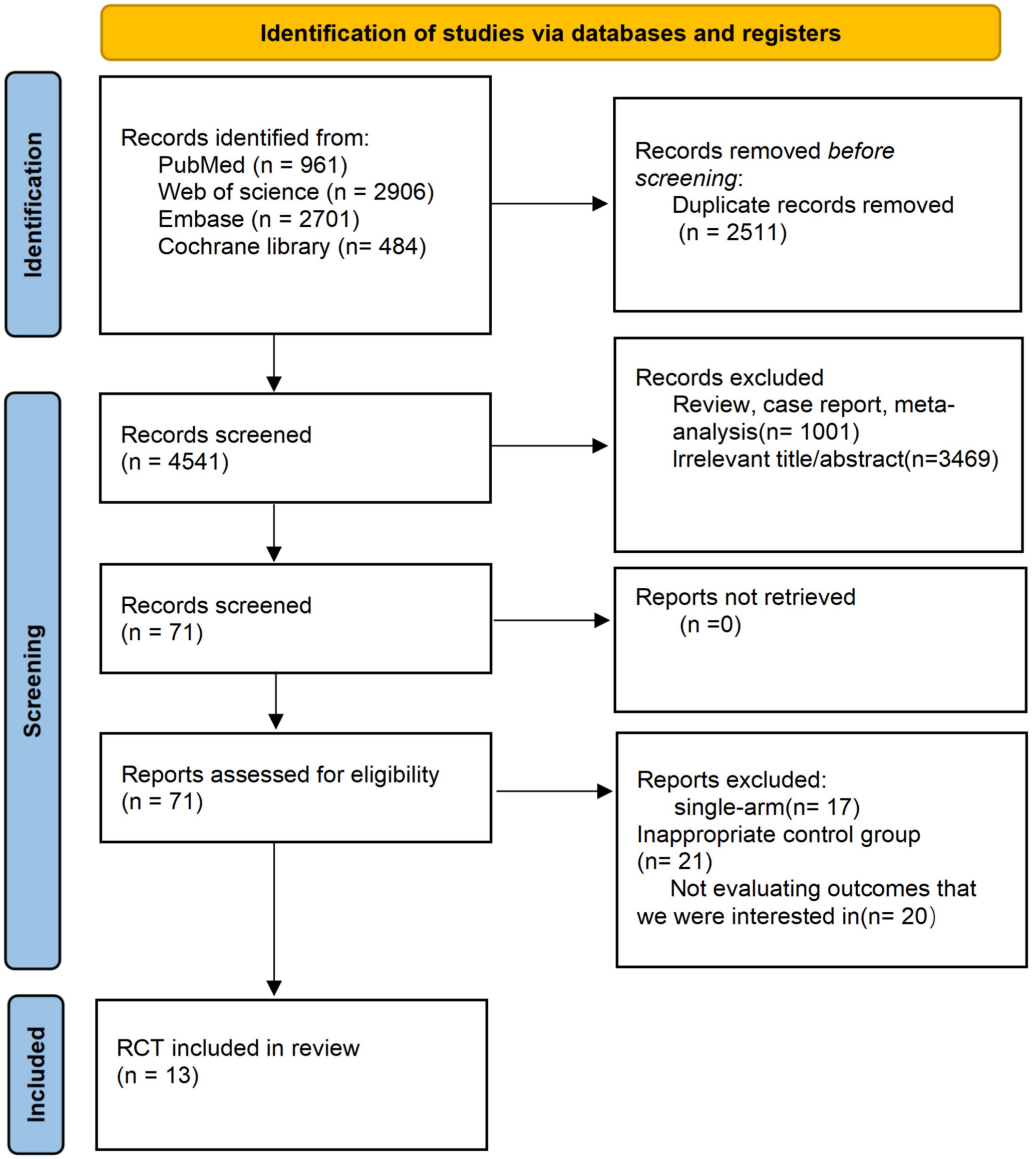
PRISMA flow diagram of study selection.

The initial database search yielded 7,052 records. After removal of duplicates and screening of titles and abstracts for relevance, 71 articles were selected for full-text assessment. Of these, 13 studies met the predefined inclusion and exclusion criteria and were included in the final analysis (Figure 1) (27–39). A total of 805 participants were included across the 13 studies and received one of four dietary interventions: TRE, CER, STF, or GD. Intervention duration varied by dietary strategy, ranging from 4 to 16 weeks for TRE, 12 to 96 weeks for CER, 3 to 24 weeks for STF, and 3 to 96 weeks for GD. The included studies were conducted across multiple international centers, including Australia (*n* = 3), the United States (*n* = 2), and Italy (*n* = 2), among others. Overall, the sample size was moderate and comprised predominantly middle-aged to older adults, with mean ages ranging from 45 to 68 years, mean BMI values from 27.5 to 35.8 kg/m^2^, and mean baseline HbA1c levels between 6.8 and 8.5%. Detailed characteristics of the included studies are presented in Table 1.

**Table 1:** Characteristics of included trials.

First author	Year	Intervention time (W)	Mean age (years)	Country	Mean BMI (kg/㎡)	Mean HbA1c (%)	Experimental group (n)	Control group (n)	Experimental intervention	Control intervention	Outcomes
S Carter	2016	12	61.5	Australia	35.5	7.4	26	25	2 days of severe energy restriction (1670–2,500 kJ/day) and 5 days of normal feeding	A 12-week moderate-intensity CER diet (5,000–6,500 kJ/day).	HbA1c
B T Corley	2018	12	59.9	New Zealand	36.7	8.3	18	19	A very low-calorie diet for two consecutive days	A very low-calorie diet for two non-consecutive days	Weight, BMI, WC, TC, HbA1c
S Carter	2019	48	61	Australia	36	7.3	51	46	A 500–600 kcal/day energy-restricted diet on two non-consecutive days each week (participants follow their normal diet on the other 5 days)	A restricted-calorie diet (1,200–1,500 kcal per day) for 7 consecutive days	HbA1c, FBG, Weight, TC, BMI
Tingting Che	2021	12	48.5	China	26.25	8.51	54	50	A 12-week restricted feeding program (10 h per day; free feeding from 08:00 to 18:00; fasting from 18:00 to 08:00)	General diet	HbA1c, FBG, Weight, TC
Charlotte Andriessen	2022	3	67.5	Netherlands	30.5	6.4	7	7	A 3-week TRE (eating within a 10-h window each day)	Spread out over 14 h	Weight, FBG
Piero Ruggenenti	2022	96	63.9	Italy	32.2	7.1	53	50	25% CER over 2 years	General diet	Weight, TC, WC, BMI
Vasiliki Pavlou	2023	24	55	USA	39	8.1	25/25	25	8-h TRE (eat only between 12 noon and 8 p.m.; do not count calories)/CER (25% daily energy restriction)	General diet	HbA1c, WC, Weight, TC, BMI
Evelyn B Parr	2024	24	55.9	Australia	33	7.6	22	21	Participants are required to restrict their daily eating time to between 10:00 a.m. and 7:00 p.m.; fasting or abstaining from caloric intake during the remaining time (after 7:00 p.m. until before 10:00 a.m. the next day). The intervention lasts for 6 months.	General diet	HbA1c, FBG, Weight, BMI
Naparat Sukkriang	2024	12	45.29	Thailand	31.91	7.75	33/33	33	16:8 Group: Participants were required to perform 16:8 intermittent fasting for 3 days per week over 3 months (fasting for 16 h and eating for 8 h); water was allowed during the fasting period. 14:10 Group: Participants were required to perform 14:10 intermittent fasting for 3 days per week over 3 months (fasting for 14 h and eating for 10 h); water was allowed during the fasting period.	General diet	HbA1c, FBG, Weight
Domenico Tricò	2024	12	67.2	Italy	29.4	6.6	12	11	A 12-week TRE diet with calorie restriction and the following macronutrient distribution (50% carbohydrates, 30% fat, and 20% protein)	A low-calorie Mediterranean diet	HbA1c, FBG, Weight
Caroline Kaercher Kramer	2025	6	56.3	Canada	32.4	6.6	23	16	6 weeks of TRE (20 h of fasting/4 h of eating)	General diet	HbA1c, FBG, Weight, BMI, WC
Maarya Mohammed Siddiqi	2025	24	56.15	India	28.55	8.15	50	50	Intermittent fasting group (after the 8-h eating window)	General diet	HbA1c, FBG, BMI, WC
Mongkontida Umphonsathien	2025	12	48.1	Thailand	32.6	7.4	15	5	Blood glucose and metabolic control: 1,000–1,200 kcal per day for 6 weeks	General diet	HbA1c, FBG, Weight, BMI

### 3.2 ROB 2 literature quality assessment

**Figure 2:**
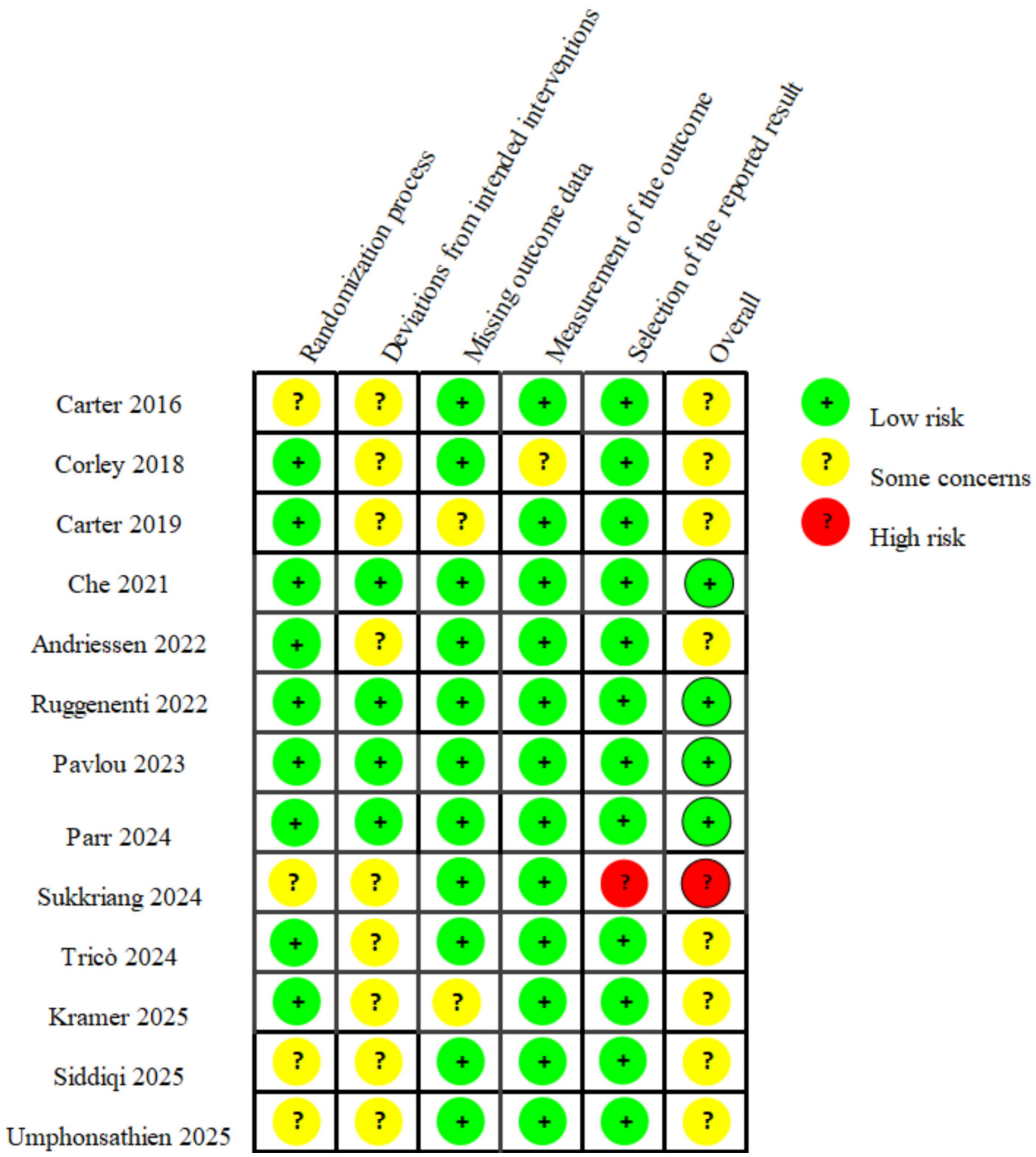
ROB 2 risk of bias assessment.

Risk of bias was evaluated for the 13 included randomized controlled trials using the Cochrane RoB 2 tool. Four studies were judged to be at low risk of bias, eight had some concerns, and one was rated as high risk, indicating an overall moderate methodological quality. For the randomization process, most trials were considered low risk, as they described appropriate sequence generation and adequate allocation concealment. Four studies raised concerns because the details of these procedures were insufficient. In the domain of deviations from intended interventions, most trials were assessed as low risk, largely on the basis of intention-to-treat analyses. However, several reports provided limited information on blinding, raising concerns in this domain. Regarding missing outcome data, attrition was generally low or appropriately addressed, resulting in low-risk judgments for most studies. Outcome measurement was also considered low risk in all but one trial, which was rated high risk due to potential measurement bias. Selective reporting was not apparent, and all trials were judged to be at low risk in this domain. Taken together, although certain methodological limitations and reporting gaps were noted, the overall risk of bias across studies is acceptable, and the main findings appear relatively stable. Nonetheless, cautious interpretation remains warranted. The detailed assessment is shown in Figure 2.

### 3.3 Direct comparisons of TRE and CER vs. GD

Pairwise meta-analyses were performed for all available direct comparisons between TRE or CER and GD. For TRE vs. GD, five studies reported HbA1c outcomes (I^2^ = 92.1%), and the random-effects model showed that TRE was associated with a significant reduction in HbA1c (MD = −0.59, 95% CI: −0.92 to −0.26). Five studies reported FBG outcomes (I^2^ = 64.5%); TRE was associated with a significant reduction in FBG (MD = −0.86 mmol/L, 95% CI: −1.25 to −0.48). Seven studies reported body weight outcomes (I^2^ = 60.1%); TRE significantly reduced body weight compared with GD (MD = −2.35 kg, 95% CI: −2.80 to −1.89). Three studies reported BMI outcomes (I^2^ = 0%), and TRE significantly reduced BMI (MD = −1.32 kg/m^2^, 95% CI: −1.72 to −0.92). Three studies reported WC outcomes (I^2^ = 0%), with TRE also showing a significant reduction in WC (MD = −3.77 cm, 95% CI: −5.17 to −2.38). Two studies reported TC outcomes (I^2^ = 2.6%), and TRE was associated with a significant reduction in TC (MD = −0.17 mmol/L, 95% CI: −0.19 to −0.14).

For CER vs. GD, two studies reported HbA1c outcomes (I^2^ = 57.3%), and CER showed a non-significant trend toward lower HbA1c levels (MD = −0.58, 95% CI: −1.39 to 0.24). Three studies reported body weight outcomes (I^2^ = 0%), and CER significantly reduced body weight (MD = −1.57 kg, 95% CI: −2.99 to −0.14). Three studies reported BMI outcomes (I^2^ = 0%), and CER was associated with a significant reduction in BMI (MD = −0.60 kg/m^2^, 95% CI: −1.15 to −0.05). Two studies reported WC outcomes (I^2^ = 42.2%), and CER significantly reduced WC (MD = −2.64 cm, 95% CI: −4.50 to −0.78). Two studies reported TC outcomes (I^2^ = 0%), and CER showed a non-significant trend toward lower TC levels than GD (MD = −0.03 mmol/L, 95% CI: −0.29 to 0.23). The detailed results are presented in Table 2 and Supplementary Figures 1–11.

**Table 2:** Pairwise meta-analysis of TRE and CER vs. GD.

Outcome indicators	Comparison of two interventions	MD	95% CI	I^2^(%)
HbA1c	TRE vs. GD	−0.59%	−0.92 to −0.26	92.10
	CER vs. GD	−0.58%	−1.39 to 0.24	57.30
FBG	TRE vs. GD	−0.86 mmol/L	−1.25 to −0.48	64.50
Weight	TRE vs. GD	−2.35 kg	−2.80 to −1.89	60.10
	CER vs. GD	−1.57 kg	−2.99 to −0.14	0.00
BMI	TRE vs. GD	−1.32 kg/m^2^	−1.72 to −0.92	0.00
	CER vs. GD	−0.60 kg/m^2^	−1.15 to −0.05	0.00
WC	TRE vs. GD	−3.77 cm	−5.17 to −2.38	0.00
	CER vs. GD	−2.64 cm	−4.50 to −0.78	42.20
TC	TRE vs. GD	−0.17 mmol/L	−0.19 to −0.14	2.60
	CER vs. GD	−0.03 mmol/L	−0.29 to 0.23	0.00

### 3.4 Homogeneity and heterogeneity

**Figure 3:**
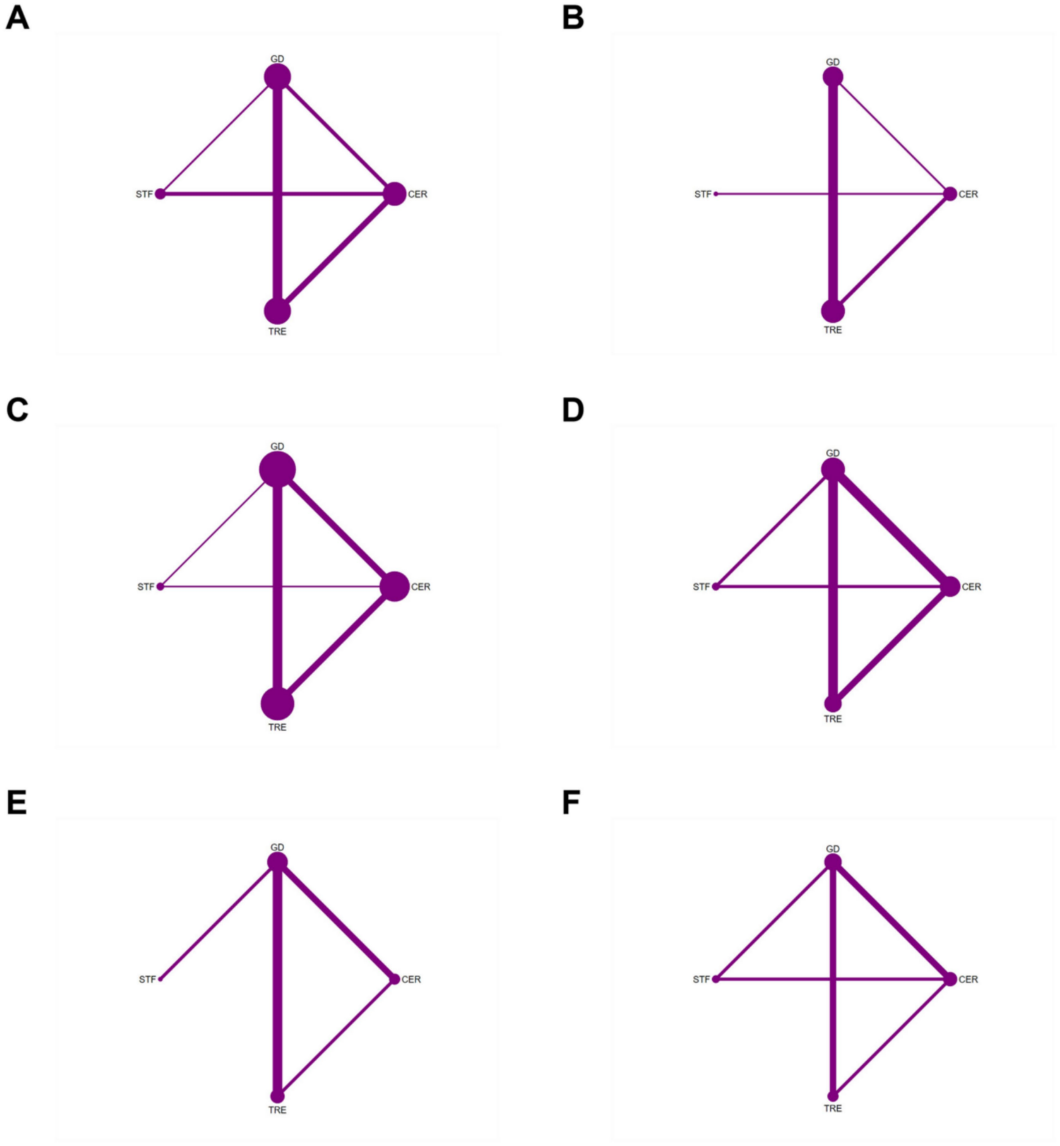
Network maps showing connections between various dietary plans for patients with type 2 diabetes who are overweight or obese. Nodes represent interventions, with node size proportional to the total sample size across included trials. Edges indicate direct comparisons between interventions, and edge thickness is proportional to the number of trials contributing to each comparison. **(A)** HbA1c; **(B)** FBG; **(C)** Weight; **(D)** BMI; **(E)** WC; **(F)** TC.

With respect to HbA1c, FBG, weight, BMI, WC, and TC, the developed evidence networks had closed loops (Figure 3). Hence, a global-inconsistency analysis was carried out, and it has been observed that all *p*-values > 0.05, and hence, there is no significant global-inconsistency (Supplementary Table 3) Node splitting for local inconsistency was also done, and all the special comparison *p*-values were > 0.05 (Supplementary Tables 4–9) Moreover, we examined the concordance of the direct and indirect data with loop inconsistency evaluation. Of all 95% in the loops that had inconsistencies, none were left out, so this is in strong agreement with the network framework (Supplementary Tables 10–15). So the main network meta-analysis was done with a consistency model.

In terms of evaluating between-study variation, the values are 0.2356, 0.3426 and 0.6516 for HbA1c, FBG and body weight. There is little heterogeneity here. On the other hand, BMI and WC’s *τ* values are less than 0.001, which means that there is no significant between-group difference (Supplementary Table 16).

### 3.5 HbA1c

**Figure 4:**
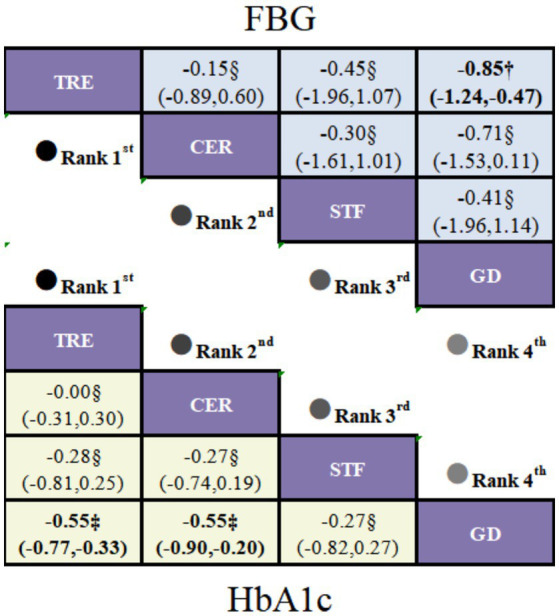
Network meta-analysis league table for HbA1c and FBG in overweight or obese patients with type 2 diabetes. Estimated treatment effects are presented in the lower-left triangle (HbA1c) and upper-right triangle (FBG). Certainty of evidence is reported according to GRADE, with * indicating high certainty, † moderate certainty, ‡ low certainty, and § very low certainty. These estimates reflect network comparisons and are not identical to pairwise meta-analysis results.

For HbA1c, 11 studies were included, encompassing 688 overweight or obese patients with T2DM and evaluating the effects of four interventions (Figure 3A). Low-certainty evidence indicated that, relative to GD, both TRE (MD = −0.55, 95% CI: −0.77 to −0.33) and CER (MD = −0.55, 95% CI: −0.90 to −0.20) were associated with significant reductions in HbA1c. By contrast, although STF also showed a potential glucose-lowering effect compared with GD, the difference did not reach statistical significance (MD = −0.27, 95% CI: −0.82 to 0.27), and the certainty of evidence was very low (Figure 4).

**Figure 5:**
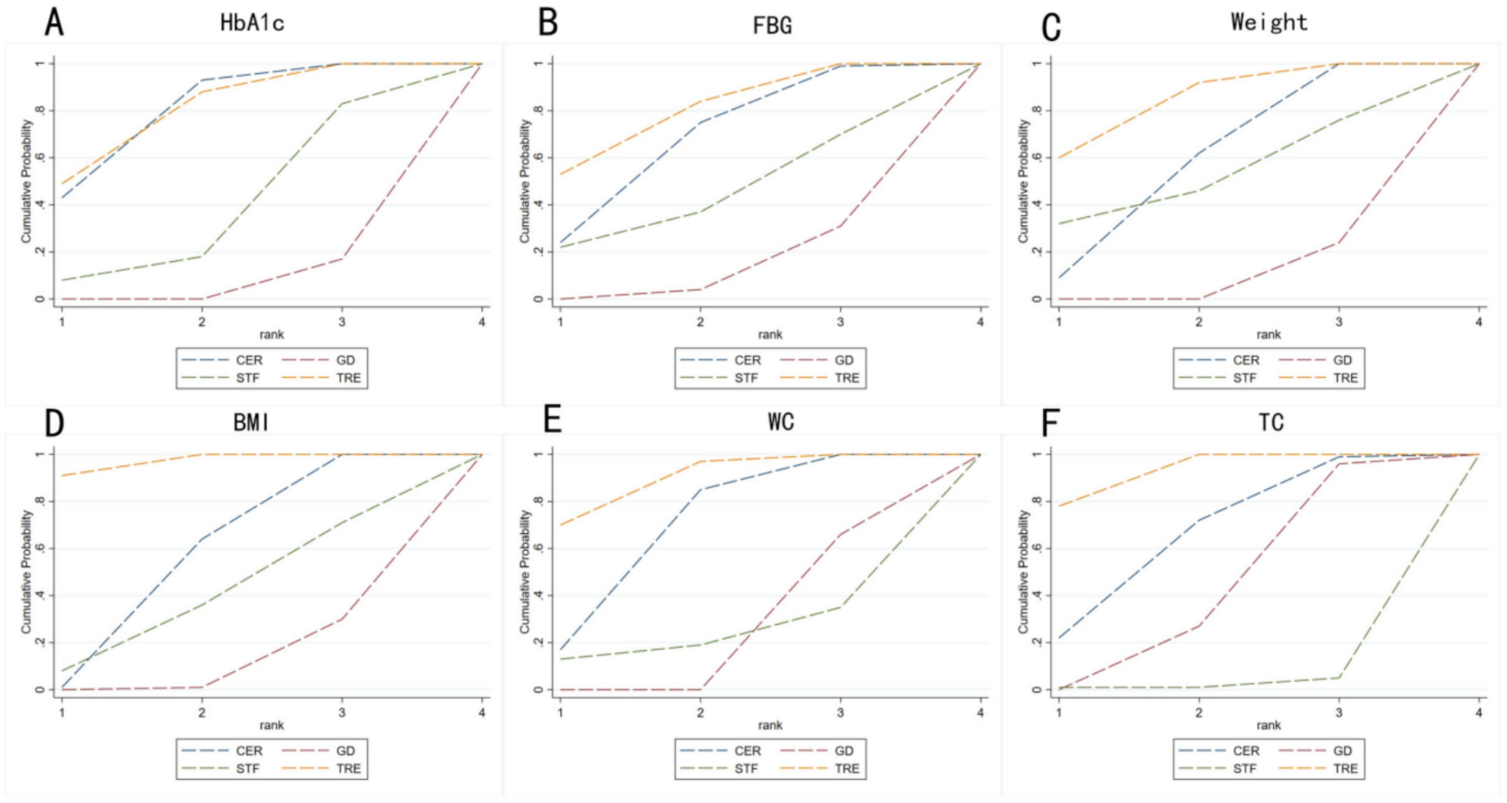
SUCRA ranking curves for each dietary intervention across various outcome measures in overweight/obese patients with type 2 diabetes. **(A)** HbA1c; **(B)** FBG; **(C)** Weight; **(D)** BMI; **(E)** WC; **(F)** TC.

According to the SUCRA estimates, TRE ranked highest (79.0%), followed closely by CER (78.9%), suggesting broadly comparable and favorable efficacy profiles for these two interventions. Rank probability analysis showed that TRE had the greatest likelihood of being the most effective intervention (48.9%), followed by CER (43.3%) and STF (7.9%). These findings were consistent with the SUCRA-based rankings. Average rank analysis further supported the relative advantage of TRE and CER, both of which had the lowest mean rank (1.6), whereas STF ranked lower, with a mean rank of 2.9 (Figure 5; Supplementary Table 17).

### 3.6 FBG

For FBG, we pooled data from 9 studies that assessed 4 interventions in 539 overweight or obese patients with T2DM (Figure 3B). Moderate-certainty evidence showed that, relative to GD, TRE significantly reduced FBG (MD = −0.85 mmol/L, 95% CI: −1.24 to −0.47). Compared with GD, CER (MD = −0.71 mmol/L, 95% CI: −1.53 to 0.11) and STF (MD = −0.41 mmol/L, 95% CI: −1.96 to 1.14) also tended to lower FBG, although neither comparison reached statistical significance; the certainty of evidence for both was very low (Figure 4).

According to the SUCRA estimates, TRE ranked first (79.1%), followed by CER (66.0%), suggesting that these two interventions were relatively more effective overall. Rank-probability analysis indicated that TRE had the highest posterior probability (53.4%) as the best intervention, followed by CER (24.4%) and STF (22.3%). These findings were consistent with the SUCRA results. Average-rank analysis further supported the advantage of TRE, which had the lowest mean rank (1.6), followed by CER (2.0; Figure 5; Supplementary Table 18).

### 3.7 Weight

**Figure 6:**
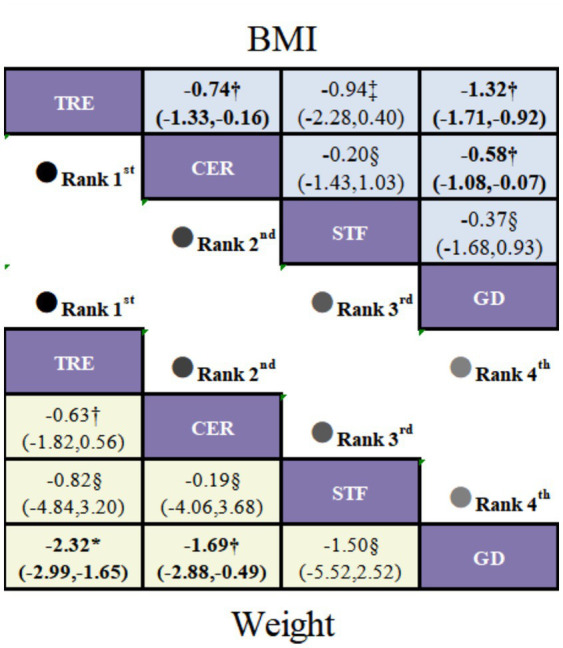
Network meta-analysis league table for body weight and BMI in overweight or obese patients with type 2 diabetes. Estimated treatment effects are presented in the lower-left triangle (body weight) and upper-right triangle (BMI). Certainty of evidence is reported according to GRADE, with * indicating high certainty, † moderate certainty, ‡ low certainty, and § very low certainty. These estimates reflect network comparisons and are not identical to pairwise meta-analysis results.

For body weight, we pooled data from 11 studies involving 654 overweight or obese patients with T2DM to compare the effects of four interventions on body weight (Figure 3C). High-certainty evidence showed that, relative to GD, TRE significantly reduced body weight (MD = −2.32 kg, 95% CI: −2.99 to −1.65). Moderate-certainty evidence likewise indicated a significant reduction with CER compared with GD (MD = −1.69 kg, 95% CI: −2.88 to −0.49). By contrast, STF was also associated with a reduction in body weight relative to GD (MD = −1.50 kg, 95% CI: −5.52 to 2.52), but this effect did not reach statistical significance and was supported by very low-certainty evidence (Figure 6).

According to the SUCRA estimates, TRE ranked highest (84.0%), followed by CER (56.8%) and STF (51.1%), suggesting that all three interventions may confer some advantage in reducing body weight. Ranking probability analysis further indicated that TRE had the greatest likelihood of being the most effective intervention (59.7%), followed by STF (31.6%). Thus, although CER ranked above STF in the SUCRA analysis, the uncertainty around STF remains substantial, and no firm comparative conclusion can be drawn. This pattern was also reflected in the mean ranking results: TRE had the lowest mean rank (1.5), followed by CER (2.3) and STF (2.5; Figure 5; Supplementary Table 19).

### 3.8 BMI

For BMI, 8 studies were included in the pooled analysis, encompassing 514 overweight or obese patients with T2DM and evaluating 4 interventions (Figure 3D). Moderate-certainty evidence indicated that, relative to GD, both TRE (MD = −1.32 kg/m^2^, 95% CI: −1.71 to −0.92) and CER (MD = −0.58 kg/m^2^, 95% CI: −1.08 to −0.07) were associated with significant reductions in BMI. By contrast, STF also showed a tendency to reduce BMI compared with GD (MD = −0.37 kg/m^2^, 95% CI: −1.68 to 0.93), although the difference was not statistically significant; the certainty of evidence was very low (Figure 6).

According to the SUCRA estimates, TRE ranked first (97.0%), followed by CER (54.6%), indicating a clear advantage of TRE in overall efficacy. Rank-probability analysis further showed that TRE had the highest probability of being the most effective intervention (91.3%), followed by STF (8.2%). Thus, although CER ranked higher than STF based on SUCRA, the available evidence for STF remains insufficient to support a firm conclusion. This pattern was also supported by the mean rank analysis: TRE had the lowest mean rank (1.1), followed by CER (2.4) and STF (2.9; Figure 5; Supplementary Table 20).

### 3.9 WC

**Figure 7:**
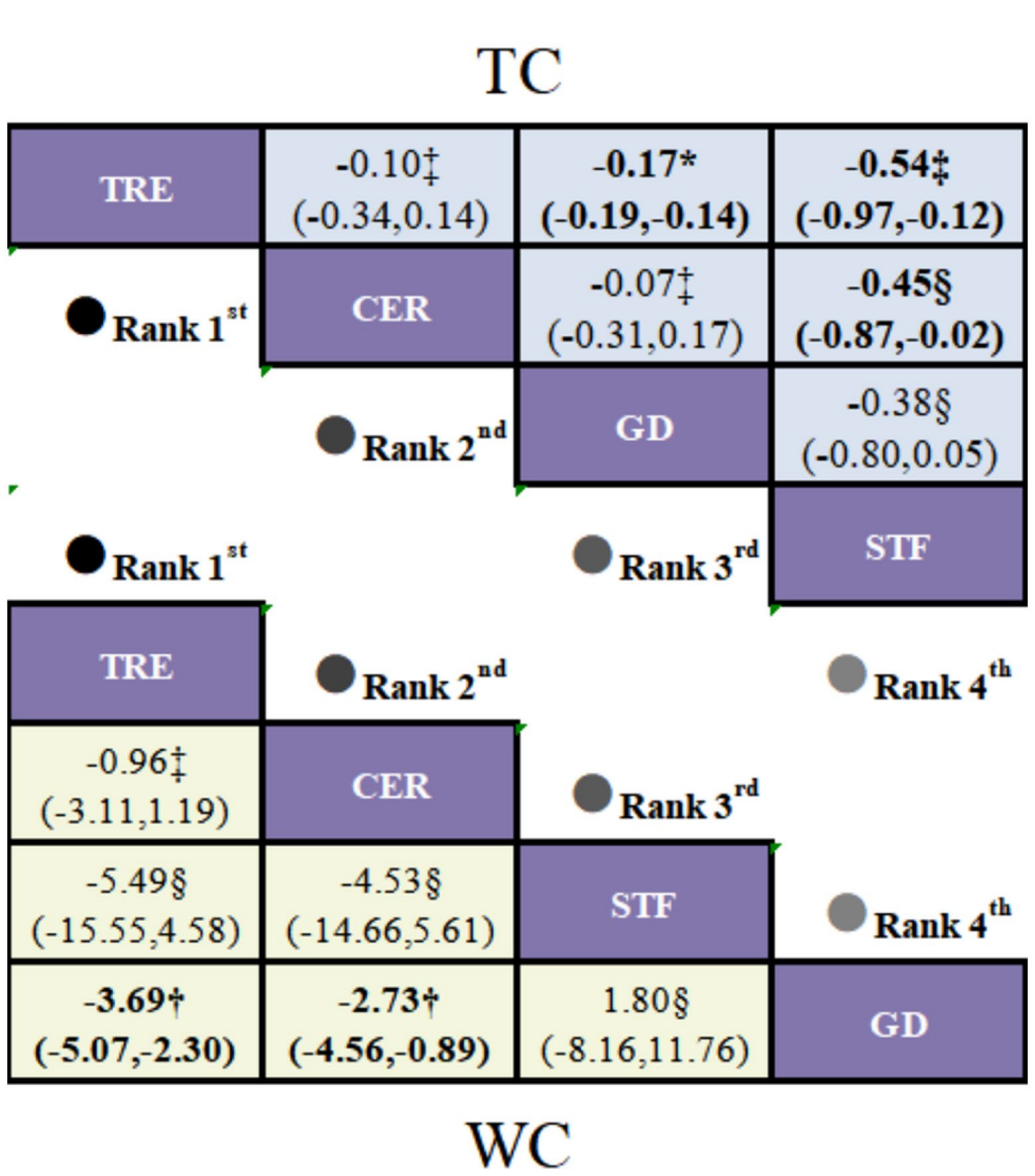
Network meta-analysis league table for waist circumference and total cholesterol in overweight or obese patients with type 2 diabetes. Estimated treatment effects are presented in the lower-left triangle (WC) and upper-right triangle (TC). Certainty of evidence is reported according to GRADE, with * indicating high certainty, † moderate certainty, ‡ low certainty, and § very low certainty. These estimates reflect network comparisons and are not identical to pairwise meta-analysis results.

For WC, we pooled data from five studies assessing four interventions in 354 overweight or obese patients with T2DM (Figure 3E). Moderate-certainty evidence indicated that, relative to GD, both TRE (MD = −3.69 cm, 95% CI: −5.07 to −2.30) and CER (MD = −2.73 cm, 95% CI: −4.56 to −0.89) were associated with significant reductions in WC. By contrast, STF did not reduce WC compared with GD (MD = 1.80 cm, 95% CI: −8.16 to 11.76), and the between-group difference was not statistically significant; the certainty of evidence was very low (Figure 7).

According to the SUCRA estimates, TRE ranked first (89.0%), followed by CER (67.2%), indicating superior overall efficacy for these two interventions. Rank probability analysis further showed that TRE had the highest probability of being the most effective intervention (70.1%), followed by CER (17.0%) and STF (12.8%). These findings were consistent with the SUCRA results. Mean rank analysis likewise supported the relative advantage of TRE and CER, with TRE ranking first (mean rank, 1.3) and CER second (mean rank, 2.0; Figure 5; Supplementary Table 21).

### 3.10 TC

For TC, five studies were included, covering four interventions in 416 overweight or obese patients with T2DM (Figure 3F). High-certainty evidence showed that, relative to GD, TRE significantly reduced TC (MD = −0.17 mmol/L, 95% CI: −0.19 to −0.14) in overweight or obese patients with T2DM. By contrast, CER was associated with a non-significant reduction in TC compared with GD (MD = −0.07 mmol/L, 95% CI: −0.31 to 0.17); the certainty of evidence was low (Figure 7).

According to the SUCRA estimates, TRE had the highest probability of being the most effective intervention (92.4%), followed by CER (64.2%), suggesting superior overall efficacy for these two approaches. The ranking probabilities showed a similar pattern: TRE was most likely to be the best intervention (posterior probability, 77.6%), followed by CER (21.9%). Mean rank analysis further supported the advantage of TRE, which had the lowest mean rank (1.2), compared with CER (2.1; Figure 5; Supplementary Table 22).

### 3.11 Subgroup analysis

**Figure 8:**
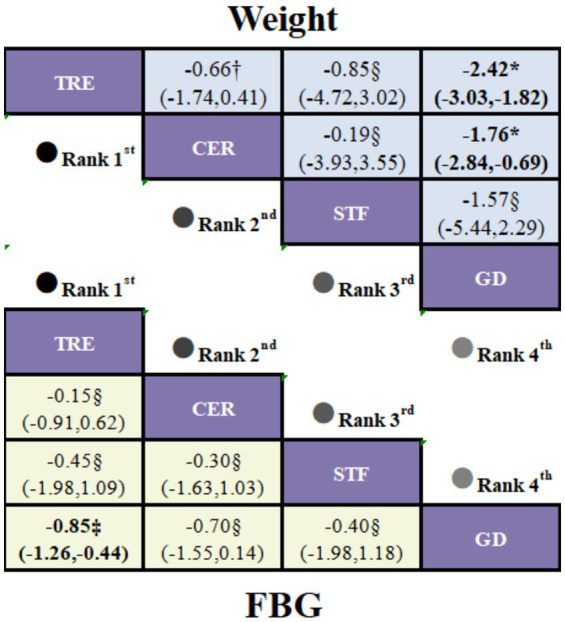
Subgroup network meta-analysis for participants with BMI > 28 kg/m^2^. Estimated treatment effects for FBG are presented in the lower-left triangle (yellow shading), whereas those for body weight are shown in the upper-right triangle (blue shading). Certainty of evidence is reported according to GRADE, with * indicating high certainty, † moderate certainty, ‡ low certainty, and § very low certainty. This figure presents subgroup-specific estimates and complements, rather than repeats, the main network analysis.

A prespecified subgroup analysis was conducted in participants with BMI > 28 kg/m^2^ to evaluate the effects of different dietary strategies on body weight and FBG. Only body weight and FBG were analyzed in this subgroup, as the number of available studies reporting the other outcomes was too limited to support reliable subgroup analyses. Compared with the GD group, both TRE (MD − 2.42 kg; 95% CI: −3.03 to −1.82) and CER (MD − 1.76 kg; 95% CI: −2.84 to −0.69) were associated with significant weight reduction in overweight or obese individuals with T2DM. STF also showed a reduction in body weight relative to GD (MD − 1.57 kg; 95% CI: −5.44 to 2.29), although this difference did not reach statistical significance. Regarding FBG, TRE produced a significant decrease compared with GD (MD − 0.85 mmol/L; 95% CI: −1.26 to −0.44) in this subgroup. CER (MD − 0.70 mmol/L; 95% CI: −1.55 to 0.14) and STF (MD − 0.40 mmol/L; 95% CI: −1.98 to 1.18) were also associated with numerically lower FBG levels than GD; however, neither comparison achieved statistical significance (Figure 8).

### 3.12 Meta-analysis, sensitivity analysis, and publication bias

To explore possible sources of heterogeneity, univariable network meta-regression analyses were conducted for the five outcomes. The baseline HbA1c, BMI, duration of treatment, age, and country of origin did not have a large effect on results, so the validity of the transitivity assumption is confirmed (Supplementary Tables 23–28).

Robustness of the pooled estimates was evaluated using leave-one-out sensitivity analyses for all outcomes, with each study removed in turn to determine its impact on the overall effect size. Excluding any individual trial did not materially affect either the direction or the magnitude of the associations. The estimates were largely unchanged, as reflected by the broad overlap of 95% confidence intervals and minimal variation in statistical significance across comparisons. Although slight shifts in significance occurred in a few pairwise comparisons after removal of specific studies, the overall network geometry and ranking pattern remained intact. Taken together, these results indicate that no single study exerted a disproportionate influence on the findings (Supplementary Tables 29–34).

Funnels were made with respect to HbA1c, FBG, body weight, BMI, WC, and TC, as well as all the sums for potential publication biases. The research in general was pretty evenly spread; there is not much visible systematic bias or extreme outliers. Although a few points seem near the edges of the graph, they do not appear to be indicative of any strong small study effect or publication bias. These findings indicate a low likelihood of substantial publication bias (Supplementary Figures 12–17).

### 3.13 Grading

We used GRADE methodology and CINeMA to evaluate the certainty of the available evidence for the main results from the network meta-analysis (Supplementary Tables 39–40): In each of the 4 network evaluations for endpoints, certainty is high in 2 cases, moderate in 8 cases, low in 7, and very low in 19. Higher certainty ratings were generally observed in comparisons informed by multiple direct head-to-head trials, with confidence intervals that did not cross the line of no effect or the MID. By contrast, low or very low certainty was typically attributable to sparse network structures or heavy reliance on indirect evidence, often together with wide confidence intervals and possible small-study effects.

## 4 Discussion

There are currently no solid head-to-head comparisons between the different nutritional intervention approaches on blood glucose levels and weight loss in overweight or obese individuals with T2DM. Therefore, we utilized a network meta-analysis to synthesize all direct and indirect evidence that was available on the effectiveness (and otherwise) of TRE, CER, and STF as dietary interventions for overweight/obese individuals with T2DM.

A total of 13 randomized controlled trials involving 805 participants with overweight or obesity and T2DM were included in this analysis. Based on MID thresholds applied within the network meta-analysis, TRE was associated with greater reductions in HbA1c and FBG than GD, and with more pronounced weight loss. Improvements were also observed with TRE in BMI, WC, and TC. CER led to significant decreases in HbA1c and FBG and was accompanied by reductions in body weight, BMI, WC, and TC. The available evidence for STF remained insufficient to draw a definitive conclusion regarding glycemic control, body weight, or WC. In participants with obesity (BMI > 28 kg/m^2^), subgroup analyses indicated that TRE resulted in greater reductions in BMI and FBG compared with GD. Both TRE and CER were associated with clinically meaningful weight loss in this subgroup, whereas STF did not show significant effects on the assessed outcomes.

TRE emerged as a potentially favorable intervention in this NMA, and its observed benefits in HbA1c, fasting plasma glucose, body weight, BMI, and waist circumference are broadly consistent with proposed chrono-nutritional mechanisms. Although mechanistic explanations from prior literature provide biological plausibility, the present study was designed to compare clinical outcomes rather than test specific pathways; therefore, these mechanisms should be viewed as indirect interpretations of the observed effect patterns. For example, TRE has been hypothesized to align food intake with endogenous circadian rhythms and to prolong daily fasting periods, which may in turn reduce pancreatic *β*-cell stress and improve insulin sensitivity (15, 40–45). Similarly, the benefits observed with CER in our analysis are compatible with its established energy-deficit mechanism and potential effects on visceral and hepatic lipid mobilization (46–49). The limited and inconclusive evidence for STF in the current network does not allow firm mechanistic inferences, although prior literature has suggested that fasting-related metabolic switching or appetite effects may contribute to weight and metabolic changes (50–52). Overall, the mechanistic literature provides biologically plausible explanations for the patterns observed in this NMA, but it should be viewed as complementary to, rather than definitive evidence for, the comparative effects reported here.

The findings of this study further underscore the potential of dietary interventions in the management of overweight or obesity in patients with T2DM, suggesting that they may serve as feasible treatment options in appropriately selected patients. Although current clinical guidelines make only limited reference to emerging dietary approaches such as TRE, the present study provides comparative evidence on their metabolic effects that may help inform more specific and clinically actionable recommendations. Given the practical advantages of TRE and CER, including low cost, minimal adverse effects, and relative ease of implementation (53, 54), these interventions may be suitable for incorporation into primary care and community-based health programs (55).

When selecting a dietary intervention, both the patient’s metabolic targets and likelihood of adherence must be carefully considered, as a “one-size-fits-all” approach is often suboptimal in the management of T2DM. In our analysis, TRE ranked favorably across several glycemic and anthropometric outcomes, which may make it an attractive option for patients seeking a relatively simple dietary approach; however, the direct comparisons did not confirm clear superiority over CER. CER also showed beneficial effects on body weight, BMI, and waist circumference, supporting its continued role as a well-established dietary strategy. Regarding STF, the available evidence remains limited and low in certainty, and current data are insufficient to draw firm conclusions about its comparative efficacy. Thus, rather than selecting a single universally preferred intervention, dietary choice should be individualized according to the patient’s broader clinical profile, including baseline BMI, diabetes duration, medication regimens, and personal preferences. We advocate for a shared decision-making process in which the clinician and patient collaboratively choose the intervention that best aligns with the patient’s lifestyle and treatment goals.

A key methodological distinction of this study lies in its approach to intervention classification. Adhering to the current international consensus (18), we maintained a strict distinction between CER, STF, and TRE, evaluating each independently. In contrast, previous syntheses—notably those by Wang (56) and Borgundvaag et al. (57)—aggregated disparate intermittent fasting protocols into a single broad category for comparison against CER. Such broad categorization likely masks the inherent physiological and clinical heterogeneity between fasting subtypes. By employing a more granular stratification, our analysis revealed a significant divergence in efficacy between TRE and STF. These findings underscore the necessity for future research to adopt protocol-specific classifications to ensure more precise evaluations of dietary interventions.

The present study showed that TRE significantly improved glycemic control and reduced body weight in overweight or obese patients with T2DM, in close agreement with the findings of Nam et al. (43), who reported reductions in HbA1c and FBG after TRE. These overlapping findings suggest that TRE may be a practical and potentially effective dietary strategy, although this study did not directly measure the circadian or behavioral mechanisms proposed in prior literature. By contrast, STF did not show clear benefit over GD across the assessed outcomes in this study, and its ranking was generally lower in the SUCRA analysis. This pattern is consistent with the limited and low-certainty evidence for STF in our network, and further studies are needed to clarify its comparative effectiveness and durability in routine clinical practice. With respect to CER, our study confirmed its effectiveness in reducing HbA1c and body weight, consistent with prior reviews (56–59). Overall, the network ranking suggested that TRE may offer favorable comparative effects for glycemic and weight-related outcomes; however, the direct comparison between TRE and CER did not reach statistical significance, so this should be interpreted as a ranking signal rather than proof of superiority.

In clinical practice, sustained adherence to dietary interventions is a major determinant of therapeutic benefit. As TRE is comparatively straightforward and does not require meticulous daily calorie counting, it may be easier to implement in everyday life. This practical feature may help explain why TRE ranked favorably in this analysis and in a prior study (43). By contrast, STF and CER may require more structured monitoring of energy intake, which could affect long-term feasibility for some patients. This difference in adherence burden may partly influence real-world effectiveness, although adherence was not directly compared in the present study.

We adopted a rigorous and transparent strategy for evidence synthesis. The methodological quality of each included trial was evaluated using the ROB 2 tool. Confidence in the pooled estimates was further assessed using the CINeMA framework, which provides a structured appraisal of the credibility of network meta-analysis results. To address clinical heterogeneity, analyses were stratified according to baseline BMI, with outcomes in participants with T2DM reported separately within the overweight subgroup. The stability of the primary findings was examined through prespecified sensitivity analyses and network meta-regression. In addition, treatment effects were interpreted in relation to established MCIDs, thereby linking statistical significance to clinical relevance. Whenever permitted by the available data, results were analyzed by the intervention category to reduce conceptual heterogeneity and to avoid conflating mechanistically distinct therapeutic approaches. This structured comparative framework enhances the interpretability and immediate clinical applicability of the findings. Furthermore, to address potential concerns regarding heterogeneity and to validate the robustness of the network meta-analysis, we also conducted pairwise meta-analyses for all available direct head-to-head comparisons. These direct comparisons provided complementary evidence to the network estimates but were not expected to match the network league-table results exactly because the two approaches address different estimands. The overall direction of effect was broadly similar across analyses, supporting the robustness of the primary findings for TRE and CER.

Several limitations warrant careful consideration. First, the evidence base remains limited, as only 13 randomized controlled trials involving 805 participants were eligible for inclusion. Second, the observation period of the included studies was short, with a maximum follow-up of 12 weeks, which limits conclusions regarding the durability of weight reduction, glycemic improvement, and long-term adherence or safety. Third, although the pooled effects on body weight and HbA1c were statistically favorable, the magnitude of benefit was modest and should be interpreted cautiously in terms of clinical relevance, particularly in the context of short-term interventions. In addition, the inherent characteristics of dietary interventions preclude full blinding. Participants are unavoidably aware of the dietary regimen to which they are assigned, and outcome assessors are not always completely masked. Although the risk of bias was evaluated rigorously using the RoB 2 tool, performance and detection bias cannot be entirely excluded in unblinded settings, particularly given the influence of expectations and subjective assessment. The geometry of the evidence network is another constraint. Not all dietary strategies were directly compared, and some contrasts relied on indirect evidence, which may reduce the precision of effect estimates and attenuate certainty in comparative inferences. Data on adverse events and extended follow-up were also limited, restricting evaluation of long-term sustainability and comprehensive benefit–risk profiles. Collectively, these limitations indicate that our findings should be interpreted as preliminary evidence and that larger, well-powered trials with longer follow-up are needed to confirm the comparative effectiveness and clinical utility of these dietary strategies.

## 5 Conclusion

In summary, both TRE and CER were associated with statistically significant improvements in several metabolic parameters, whereas the current evidence supporting STF remains less conclusive. TRE ranked favorably across multiple outcomes, but direct comparisons with CER were generally not statistically significant. Therefore, TRE may be considered a promising dietary strategy, while the choice of dietary intervention should be individualized according to the patient’s clinical profile, treatment goals, and preferences. Further, adequately powered trials with longer follow-up are needed to clarify the comparative advantages and sustainability of these interventions.

## Data Availability

The original contributions presented in the study are included in the article/Supplementary material, further inquiries can be directed to the corresponding author.
