# Supplementary material for: Comparative efficacy of dietary interventions for overweight or obese adults with type 2 diabetes: a systematic review and network meta-analysis of randomized controlled trials
Source: Front Nutr. 2026 Jul 15;13:1853598. doi: 10.3389/fnut.2026.1853598 (PMC13414187; doi:10.3389/fnut.2026.1853598)
Supplement: Supplementary file 1 [file Supplementary_file_1.docx]

Comparative Efficacy of Dietary Interventions for Overweight or Obese Adults With Type 2 Diabetes: A Systematic Review and Network Meta-analysis of Randomized Controlled Trials

| **Table of Contents** | | |
| --- | --- | --- |
| Title | Content | page |
| Table S1 | PRISMA NMA Checklist of Items to Include When Reporting a Systematic Review Involving a Network Meta-analysis | 2-5 |
| Table S2 | Literature Search Strategy | 6-10 |
| Figure S1 | Forest plot of TRE versus GD for HbA1c | 11 |
| Figure S2 | Forest plot of TRE versus GD for FBG | 11 |
| Figure S3 | Forest plot of TRE versus GD for Weight | 12 |
| Figure S4 | Forest plot of TRE versus GD for BMI | 12 |
| Figure S5 | Forest plot of TRE versus GD for WC | 13 |
| Figure S6 | Forest plot of TRE versus GD for TC | 13 |
| Figure S7 | Forest plot of CER versus GD for HbA1c | 14 |
| Figure S8 | Forest plot of CER versus GD for Weight | 14 |
| Figure S9 | Forest plot of CER versus GD for BMI | 15 |
| Figure S10 | Forest plot of CER versus GD for WC | 15 |
| Figure S11 | Forest plot of CER versus GD for TC | 16 |
| Table S3 | Global inconsistency table for HbA1c, FBG, Weight, BMI, WC and TC outcomes in overweight/obese individuals with type 2 diabetes | 16 |
| Table S4 | Node-splitting analysis of HbA1c results in overweight/obese individuals with type 2 diabetes | 16 |
| Table S5 | Node-splitting analysis of FBG results in overweight/obese individuals with type 2 diabetes | 16 |
| Table S6 | Node-splitting analysis of Weight results in overweight/obese individuals with type 2 diabetes | 17 |
| Table S7 | Node-splitting analysis of BMI results in overweight/obese individuals with type 2 diabetes | 17 |
| Table S8 | Node-splitting analysis of WC results in overweight/obese individuals with type 2 diabetes | 17 |
| Table S9 | Node-splitting analysis of TC results in overweight/obese individuals with type 2 diabetes | 17 |
| Table S10 | Loop inconsistency plot for the HbA1c results in overweight/obese individuals with type 2 diabetes | 17 |
| Table S11 | Loop inconsistency plot for the FBG results in overweight/obese individuals with type 2 diabetes | 17 |
| Table S12 | Loop inconsistency plot for the Weight results in overweight/obese individuals with type 2 diabetes | 18 |
| Table S13 | Loop inconsistency plot for the BMI results in overweight/obese individuals with type 2 diabetes | 18 |
| Table S14 | Loop inconsistency plot for the WC results in overweight/obese individuals with type 2 diabetes | 18 |
| Table S15 | Loop inconsistency plot for the TC results in overweight/obese individuals with type 2 diabetes | 18 |
| Table S16 | Assessment Table of Heterogeneity (τ Values) for Clinical Outcome Measures in Overweight/Obese Patients with Type 2 Diabetes | 18 |
| Table S17 | SUCRA ranking table for the HbA1c outcome in overweight/obese individuals with type 2 diabetes | 18 |
| Table S18 | SUCRA ranking table for the FBG outcome in overweight/obese individuals with type 2 diabetes | 18-19 |
| Table S19 | SUCRA ranking table for the Weight outcome in overweight/obese individuals with type 2 diabetes | 19 |
| Table S20 | SUCRA ranking table for the BMI outcome in overweight/obese individuals with type 2 diabetes | 19 |
| Table S21 | SUCRA ranking table for the WC outcome in overweight/obese individuals with type 2 diabetes | 19 |
| Table S22 | SUCRA ranking table for the TC outcome in overweight/obese individuals with type 2 diabetes | 19 |
| Table S23 | Meta-regression analysis of HbA1c outcomes in overweight/obese patients with type 2 diabetes, with HbA1c, BMI, duration of intervention, age and country as moderator variables | 19-20 |
| Table S24 | Meta-regression analysis of FBG outcomes in overweight/obese patients with type 2 diabetes, with HbA1c, BMI, duration of intervention, age and country as moderator variables | 21-22 |
| Table S25 | Meta-regression analysis of Weight outcomes in overweight/obese patients with type 2 diabetes, with HbA1c, BMI, duration of intervention, age and country as moderator variables | 22-23 |
| Table S26 | Meta-regression analysis of BMI outcomes in overweight/obese patients with type 2 diabetes, with HbA1c, BMI, duration of intervention, age and country as moderator variables | 23-24 |
| Table S27 | Meta-regression analysis of WC outcomes in overweight/obese patients with type 2 diabetes, with HbA1c, BMI, duration of intervention, age and country as moderator variables | 25-26 |
| Table S28 | Meta-regression analysis of TC outcomes in overweight/obese patients with type 2 diabetes, with HbA1c, BMI, duration of intervention, age and country as moderator variables | 26-27 |
| Table S29 | Sensitivity analysis for the HbA1c outcome in overweight/obese patients with type 2 diabetes | 27-28 |
| Table S30 | Sensitivity analysis for the FBG outcome in overweight/obese patients with type 2 diabetes | 28-29 |
| Table S31 | Sensitivity analysis for the Weight outcome in overweight/obese patients with type 2 diabetes | 29-30 |
| Table S32 | Sensitivity analysis for the BMI outcome in overweight/obese patients with type 2 diabetes | 30 |
| Table S33 | Sensitivity analysis for the WC outcome in overweight/obese patients with type 2 diabetes | 30-31 |
| Table S34 | Sensitivity analysis for the TC outcome in overweight/obese patients with type 2 diabetes | 31 |
| Figure S12 | Comparison-adjusted funnel plot for the HbA1c outcome in overweight/obese patients with type 2 diabetes | 31 |
| Figure S13 | Comparison-adjusted funnel plot for the FBG outcome in overweight/obese patients with type 2 diabetes | 32 |
| Figure S14 | Comparison-adjusted funnel plot for the Weight outcome in overweight/obese patients with type 2 diabetes | 32 |
| Figure S15 | Comparison-adjusted funnel plot for the BMI outcome in overweight/obese patients with type 2 diabetes | 33 |
| Figure S16 | Comparison-adjusted funnel plot for the WC outcome in overweight/obese patients with type 2 diabetes | 33 |
| Figure S17 | Comparison-adjusted funnel plot for the TC outcome in overweight/obese patients with type 2  diabetes | 34 |
| Table S35 | GRADE certainty of evidence for the HbA1c outcome in overweight/obese patients with type 2 diabetes | 34 |
| Table S36 | GRADE certainty of evidence for the FBG outcome in overweight/obese patients with type 2 diabetes | 34 |
| Table S37 | GRADE certainty of evidence for the Weight outcome in overweight/obese patients with type 2 diabetes | 34-35 |
| Table S38 | GRADE certainty of evidence for the BMI outcome in overweight/obese patients with type 2 diabetes | 35 |
| Table S39 | GRADE certainty of evidence for the WC outcome in overweight/obese patients with type 2 diabetes | 35 |
| Table S40 | GRADE certainty of evidence for the TC outcome in overweight/obese patients with type 2 diabetes | 35 |

Table S1 PRISMA NMA Checklist of Items to Include When Reporting a Systematic Review Involving a Network Meta-analysis

| Section/Topic | Item # | Checklist Item | Reported on section # |
| --- | --- | --- | --- |
| TITLE |  |  |  |
| Title | 1 | Identify the report as a systematic review incorporating  anetwork meta-analysis (or related form of meta-analysis). | See the title and abstract |
| ABSTRACT |  |  |  |
| Structured summary | 2 | Provide a structured summary including, as applicable:  **Background:** main objectives  **Methods:** data sources; study eligibility criteria, participants, and interventions; study appraisal; and *synthesis methods, such as network meta-analysis.*  **Results:** number of studies and participants identified; summary estimates with corresponding confidence/credible intervals; treatment rankings may also be discussed. Authors may choose to summarize pairwise comparisons against a chosen treatment included in their analyses for brevity.  **Discussion/Conclusions:** limitations; conclusions and implications of findings.  **Other:** systematic review registration number with registry name. | See the abstract for details. |
| INTRODUCTION |  |  |  |
|  | 3 | Describe the rationale for the review in the context of what is already known*, including mention of why a network meta-analysis has been conducted.* | Section 1 |
|  | 4 | Provide an explicit statement of questions being addressed, with reference to participants, interventions, comparisons, outcomes, and study design (PICOS). | Section 1 |
| METHODS |  |  |  |
| Protocol and registration | 5 | Indicate whether a review protocol exists and if and where it can be accessed (e.g., Web address); and, if available, provide registration information, including registration number. | Section 2.1 |
| Eligibility criteria | 6 | Specify study characteristics (e.g., PICOS, length of follow-up) and report characteristics (e.g., years considered, language, publication status) used as criteria for eligibility, giving rationale. *Clearly describe eligible treatments included in the treatment network, and note whether any have been clustered or merged into the same node (with justification).* | Section 2.3 |
| Information sources | 7 | Describe all information sources (e.g., databases with dates of coverage, contact with study authors to identify additional studies) in the search and date last searched. | Section 2.2 |
| Search | 8 | Present full electronic search strategy for at least one database, including any limits used, such that it could be repeated. | Supplementary TableS2 |
| Study selection | 9 | State the process for selecting studies (i.e., screening, eligibility, included in systematic review, and, if applicable, included in the meta-analysis). | Table 1 |
| Data collection process | 10 | Describe method of data extraction from reports (e.g., piloted forms, independently, in duplicate) and any processes for obtaining and confirming data from investigators. | Section 2.4 |
| Data items | 11 | List and define all variables for which data were sought (e.g., PICOS, funding sources) and any assumptions and simplifications made. | Section 2.4 |
| Geometry of the network | S1 | Describe methods used to explore the geometry of the treatment network under study and potential biases related to it. This should include how the evidence base has been graphically summarized for presentation, and what characteristics were compiled and used to describe the evidence base to readers. | Section 2.6 |
| Risk of bias within individual studies | 12 | Describe methods used for assessing risk of bias of individual studies (including specification of whether this was done at the study or outcome level), and how this information is to be used in any data synthesis. | Section 2.5 |
| Summary measures | 13 | State the principal summary measures (e.g., risk ratio, difference in means). Also describe the use of additional summary measures assessed, such as treatment rankings and surface under the cumulative ranking curve (SUCRA) values, as well as modified approaches used to present summary findings from meta-analyses. | Section 2.6 |
| Planned methods of analysis | 14 | Describe the methods of handling data and combining results of studies for each network meta-analysis. This should include, but not be limited to:   - Handling of multi-arm trials; - Selection of variance structure; - Selection of prior distributions in Bayesian analyses; and - Assessment of model fit. | Section 2.6 |
| Assessment of Inconsistency | S2 | Describe the statistical methods used to evaluate the agreement of direct and indirect evidence in the treatment network(s) studied. Describe efforts taken to address its presence when found. | Section 2.6 |
| Risk of bias across studies | 15 | Specify any assessment of risk of bias that may affect the cumulative evidence (e.g., publication bias, selective reporting within studies). | Section 2.7 |
| Additional analyses | 16 | Describe methods of additional analyses if done, indicating which were pre-specified. This may include, but not be limited to, the following:   - Sensitivity or subgroup analyses; - Meta-regression analyses; - Alternative formulations of the treatment network; and - Use of alternative prior distributions for Bayesian analyses (if applicable). | Section 2.6 |
| RESULTS† |  |  |  |
| Study selection | 17 | Give numbers of studies screened, assessed for eligibility, and included in the review, with reasons for exclusions at each stage, ideally with a flow diagram. | Section 3.1，Fig1 |
| Presentation of network structure | S3 | Provide a network graph of the included studies to enable visualization of the geometry of the treatment network. | Section 3.4，Fig3 |
| Summary of network geometry | S4 | Provide a brief overview of characteristics of the treatment network. This may include commentary on the abundance of trials and randomized patients for the different interventions and pairwise comparisons in the network, gaps of evidence in the treatment network, and potential biases reflected by the network structure. | Section 3.1 and Section 3.4 |
| Study characteristics | 18 | For each study, present characteristics for which data were extracted (e.g., study size, PICOS, follow-up period) and provide the citations. | Section 3.1,Table 1 |
| Risk of bias within studies | 19 | Present data on risk of bias of each study and, if available, any outcome level assessment. | Section 2.5 and Section 3.2,Figure 2 |
| Results of individual studies | 20 | For all outcomes considered (benefits or harms), present, for each study: 1) simple summary data for each intervention group, and 2) effect estimates and confidence intervals. *Modified approaches may be needed to deal with information from larger networks.* | Section 3.3 |
| Synthesis of results | 21 | Present results of each meta-analysis done, including confidence/credible intervals. In larger networks, authors may focus on comparisons versus a particular comparator (e.g. placebo or standard care), with full findings presented in an appendix. League tables and forest plots may be considered to summarize pairwise comparisons. If additional summary measures were explored (such as treatment rankings), these should also be presented. | Section 3.3 to Section 3.10 |
| Exploration for inconsistency | S5 | Describe results from investigations of inconsistency. This may include such information as measures of model fit to compare consistency and inconsistency models, *P* values from statistical tests, or summary of inconsistency estimates from different parts of the treatment network. | Section 3.4 |
| Risk of bias across studies | 22 | Present results of any assessment of risk of bias across studies for the evidence base being studied. | Section 3.2 and Section 3.12, Supplementary table S10-15 |
| Results of additional analyses | 23 | Give results of additional analyses, if done (e.g., sensitivity or subgroup analyses, meta-regression analyses*, alternative network geometries studied, alternative choice of prior distributions for Bayesian analyses,* and so forth). | Section 3.11 and Section 3.12 |
| DISCUSSION |  |  |  |
| Summary of evidence | 24 | Summarize the main findings, including the strength of evidence for each main outcome; consider their relevance to key groups (e.g., healthcare providers, users, and policy-makers). | Section 3.13 and Section 4 |
| Limitations | 25 | Discuss limitations at study and outcome level (e.g., risk of bias), and at review level (e.g., incomplete retrieval of identified research, reporting bias). *Comment on the validity of the assumptions, such as transitivity and consistency. Comment on any concerns regarding network geometry (e.g., avoidance of certain comparisons).* | Section 4 |
| Conclusions | 26 | Provide a general interpretation of the results in the context of other evidence, and implications for future research. | Section 5 |
|  |  |  |  |
| FUNDING |  |  |  |
| Funding | 27 | Describe sources of funding for the systematic review and other support (e.g., supply of data); role of funders for the systematic review. This should also include information regarding whether funding has been received from manufacturers of treatments in the network and/or whether some of the authors are content experts with professional conflicts of interest that could affect use of treatments in the network. | Funding statement |

PICOS = population, intervention, comparators, outcomes, study design.

* Text in italics indicate S wording specific to reporting of network meta-analyses that has been added to guidance from the PRISMA statement.

† Authors may wish to plan for use of appendices to present all relevant information in full detail for items in this section.

| **Table S2.Literature Search Strategy** | |
| --- | --- |
| Pubmed | #1"Obesity"[Mesh]"Obesity"[MeSH Terms] 291437  #2 ((((((((Obesity[Title/Abstract]) OR (obese[Title/Abstract])) OR (overweight[Title/Abstract])) OR (adiposity[Title/Abstract])) OR (obesity, abdominal[Title/Abstract])) OR (central obesity[Title/Abstract])) OR (visceral obesity[Title/Abstract])) OR (morbid obesity[Title/Abstract])) OR (severe obesity[Title/Abstract]) 490761  #3 ("Obesity"[Mesh]) OR (((((((((Obesity[Title/Abstract]) OR (obese[Title/Abstract])) OR (overweight[Title/Abstract])) OR (adiposity[Title/Abstract])) OR (obesity, abdominal[Title/Abstract])) OR (central obesity[Title/Abstract])) OR (visceral obesity[Title/Abstract])) OR (morbid obesity[Title/Abstract])) OR (severe obesity[Title/Abstract])) 539681  #4 "Overweight"[Mesh] 303606  #5 (((((Overweight[Title/Abstract]) OR (overweight[Title/Abstract])) OR (over weight[Title/Abstract])) OR (over-weight[Title/Abstract])) OR (pre-obese[Title/Abstract])) OR (excess weight[Title/Abstract]) 110412  #6 ("Overweight"[Mesh]) OR ((((((Overweight[Title/Abstract]) OR (overweight[Title/Abstract])) OR (over weight[Title/Abstract])) OR (over-weight[Title/Abstract])) OR (pre-obese[Title/Abstract])) OR (excess weight[Title/Abstract])) 347015  #7 "Diabetes Mellitus, Type 2"[Mesh] 196306  #8 (((((((((Diabetes Mellitus, Type 2[Title/Abstract]) OR (type 2 diabetes[Title/Abstract])) OR (type II diabetes[Title/Abstract])) OR (T2DM[Title/Abstract])) OR (T2D[Title/Abstract])) OR (type 2 diabetes mellitus[Title/Abstract])) OR (type II diabetes mellitus[Title/Abstract])) OR (adult-onset diabetes[Title/Abstract])) OR (non-insulin dependent diabetes[Title/Abstract])) OR (NIDDM[Title/Abstract]) 223163  #9 ("Diabetes Mellitus, Type 2"[Mesh]) OR ((((((((((Diabetes Mellitus, Type 2[Title/Abstract]) OR (type 2 diabetes[Title/Abstract])) OR (type II diabetes[Title/Abstract])) OR (T2DM[Title/Abstract])) OR (T2D[Title/Abstract])) OR (type 2 diabetes mellitus[Title/Abstract])) OR (type II diabetes mellitus[Title/Abstract])) OR (adult-onset diabetes[Title/Abstract])) OR (non-insulin dependent diabetes[Title/Abstract])) OR (NIDDM[Title/Abstract])) 282412  #10 #3 or #6 542733  #11 #9 and #10 64404  #12 "Intermittent Fasting"[Mesh] 483  #13 ((((((((Intermittent Fasting[Title/Abstract]) OR (alternate-day fasting[Title/Abstract])) OR (alternate day fasting[Title/Abstract])) OR (alternate-day diet[Title/Abstract])) OR (alternate day diet[Title/Abstract])) OR (ADF[Title/Abstract])) OR (alternate day intermittent fasting[Title/Abstract])) OR (every other day fasting[Title/Abstract])) OR (every-other-day fasting[Title/Abstract]) 6330  #14 ("Intermittent Fasting"[Mesh]) OR (((((((((Intermittent Fasting[Title/Abstract]) OR (alternate-day fasting[Title/Abstract])) OR (alternate day fasting[Title/Abstract])) OR (alternate-day diet[Title/Abstract])) OR (alternate day diet[Title/Abstract])) OR (ADF[Title/Abstract])) OR (alternate day intermittent fasting[Title/Abstract])) OR (every other day fasting[Title/Abstract])) OR (every-other-day fasting[Title/Abstract])) 6402  #15 "Fasting"[Mesh] 40836  #16 (((((((Fasting[Title/Abstract]) OR (short-term fasting[Title/Abstract])) OR (short term fasting[Title/Abstract])) OR (short-term fast[Title/Abstract])) OR (short term fast[Title/Abstract])) OR (short fast[Title/Abstract])) OR (brief fasting[Title/Abstract])) OR (acute fasting[Title/Abstract]) 147206  #17 ("Fasting"[Mesh]) OR ((((((((Fasting[Title/Abstract]) OR (short-term fasting[Title/Abstract])) OR (short term fasting[Title/Abstract])) OR (short-term fast[Title/Abstract])) OR (short term fast[Title/Abstract])) OR (short fast[Title/Abstract])) OR (brief fasting[Title/Abstract])) OR (acute fasting[Title/Abstract])) 160205  #18 (((((((Intermittent Fasting[Title/Abstract]) OR (time-restricted eating[Title/Abstract])) OR (time restricted eating[Title/Abstract])) OR (time-restricted feeding[Title/Abstract])) OR (time restricted feeding[Title/Abstract])) OR (TRE[Title/Abstract])) OR (TRF[Title/Abstract])) OR (time-limited eating[Title/Abstract]) 10226  #19 ("Intermittent Fasting"[Mesh]) OR ((((((((Intermittent Fasting[Title/Abstract]) OR (time-restricted eating[Title/Abstract])) OR (time restricted eating[Title/Abstract])) OR (time-restricted feeding[Title/Abstract])) OR (time restricted feeding[Title/Abstract])) OR (TRE[Title/Abstract])) OR (TRF[Title/Abstract])) OR (time-limited eating[Title/Abstract])) 10268  #20 "Caloric Restriction"[Mesh] 8040  #21 (((Caloric Restriction[Title/Abstract]) OR (continuous energy restriction[Title/Abstract])) OR (continuous calorie restriction[Title/Abstract])) OR (continuous caloric restriction[Title/Abstract]) 6479  #22 ("Caloric Restriction"[Mesh]) OR ((((Caloric Restriction[Title/Abstract]) OR (continuous energy restriction[Title/Abstract])) OR (continuous calorie restriction[Title/Abstract])) OR (continuous caloric restriction[Title/Abstract])) 11790  #23 #14 or #17 or #19 or #22 18263  #24 #11 and #23 10070  #25 #11 and #23 Randomized Controlled Trial 961 |
|  |  |
|  |  |
|  |  |
|  |  |
|  |  |
|  |  |
| Web of Science | #1 TS=("Obesity" or "obesity" or "obese" or "overweight" or "adiposity" or "obesity, abdominal" or "central obesity" or "visceral obesity" or "morbid obesity" or "severe obesity") 658588  #2 TS=("Overweight" or "overweight" or "over weight" or "over-weight" or "pre-obese" or "excess weight") 155050  #3 TS=("Diabetes Mellitus, Type 2" or "type 2 diabetes" or "type II diabetes" or "T2DM" or "T2D" or "type 2 diabetes mellitus" or "type II diabetes mellitus" or "adult-onset diabetes" or "non-insulin dependent diabetes" or "NIDDM" or "type-2 diabetes") 284496  #4 #1 OR #2 660057  #5 #3 AND #4 70512  #6 TS=("Intermittent Fasting" or "alternate-day fasting" or "alternate day fasting" or "alternate-day diet" or "alternate day diet" or ADF or "alternate day intermittent fasting" or "every other day fasting" or "every-other-day fasting") 14576  #7 TS=("Fasting" or "short-term fasting" or "short term fasting" or "short-term fast" or "short term fast" or "brief fasting" or "acute fasting") 139934  #8 TS=("Intermittent Fasting" or "time-restricted eating" or "time restricted eating" or "time-restricted feeding" or "time restricted feeding" or TRE or TRF or "time-limited eating") 14886  #9 TS=("Caloric Restriction" or "continuous energy restriction" or "continuous calorie restriction" or "continuous caloric restriction" or "continuous energy restriction (CER)" or "daily calorie restriction" or "daily energy restriction" or "continuous dietary restriction") 12010  #10 #6 OR #7 OR #8 OR #9 173605  #11 #5 AND #10 11511  #12 TS=(  random* OR randomi* OR randomly  OR placebo* OR sham  OR trial OR "clinical trial" OR "controlled clinical trial"  OR "double blind" OR "single blind" OR "triple blind"  OR crossover OR "cross over" OR "parallel group"  OR cluster random*  OR RCT  ) 4433704  #13 #11 AND #12 2906 |
|  |  |
|  |  |
|  |  |
|  |  |
|  |  |
|  |  |
|  |  |
| Cochrane | #1 MeSH descriptor: [Obesity] explode all trees 22903  #2 obesity or obese or overweight or adiposity or "obesity, abdominal" or "central obesity" or "visceral obesity" or "morbid obesity" or "severe obesity" 72731  #3 #1 or #2 72835  #4 MeSH descriptor: [Overweight] explode all trees 26703  #5 overweight or "over weight" or "over-weight" or "pre-obese" or "excess weight" 26627  #6 #4 or #5 41359  #7 #3 or #6 72986  #8 MeSH descriptor: [Diabetes Mellitus, Type 2] explode all trees 27496  #9 "type 2 diabetes" or "type II diabetes" or "T2DM" or "T2D" or "type 2 diabetes mellitus" or "type II diabetes mellitus" or "adult-onset diabetes" or "non-insulin dependent diabetes" or "NIDDM" or "type-2 diabetes" 56802  #10 #8 or #9 60724  #11 #7 and #10 11832  #12 MeSH descriptor: [Intermittent Fasting] explode all trees 100  #13 "alternate-day fasting" or "alternate day fasting" or "alternate-day diet" or "alternate day diet" or ADF or "alternate day intermittent fasting" or "every other day fasting" or "every-other-day fasting" 267  #14 #12 or #13 358  #15 MeSH descriptor: [Fasting] explode all trees 4657  #16 "short-term fasting" or "short term fasting" or "short-term fast" or "short term fast" or "brief fasting" or "acute fasting" 119  #17 #15 or #16 4726  #18 MeSH descriptor: [Intermittent Fasting] explode all trees 100  #19 "time-restricted eating" or "time restricted eating" or "time-restricted feeding" or "time restricted feeding" or TRE or TRF or "time-limited eating" 1896  #20 #18 or #19 1944  #21 MeSH descriptor: [Caloric Restriction] explode all trees 1268  #22 "continuous energy restriction" or "continuous calorie restriction" or "continuous caloric restriction" or "continuous energy restriction (CER)" or "daily calorie restriction" or "daily energy restriction" or "continuous dietary restriction" 234  #23 #21 or #22 1436  #24 #14 or #17 or #20 or #23 7904  #25 #11 and #24 484 |
|  |  |
|  |  |
|  |  |
| Embase | #1 'adult onset diabetes'/exp OR 'adult onset diabetes' OR 'adult onset diabetes mellitus'/exp OR 'adult onset diabetes mellitus' OR 'diabetes mellitus type 2'/exp OR 'diabetes mellitus type 2' OR 'diabetes mellitus type ii'/exp OR 'diabetes mellitus type ii' OR 'diabetes mellitus, maturity onset'/exp OR 'diabetes mellitus, maturity onset' OR 'diabetes mellitus, non insulin dependent'/exp OR 'diabetes mellitus, non insulin dependent' OR 'diabetes mellitus, non-insulin-dependent'/exp OR 'diabetes mellitus, non-insulin-dependent' OR 'diabetes mellitus, type 2'/exp OR 'diabetes mellitus, type 2' OR 'diabetes mellitus, type ii'/exp OR 'diabetes mellitus, type ii' OR 'diabetes type 2'/exp OR 'diabetes type 2' OR 'diabetes type ii'/exp OR 'diabetes type ii' OR 'diabetes, adult onset'/exp OR 'diabetes, adult onset' OR 'dm 2'/exp OR 'dm 2' OR 'insulin independent diabetes'/exp OR 'insulin independent diabetes' OR 'insulin independent diabetes mellitus'/exp OR 'insulin independent diabetes mellitus' OR 'ketosis resistant diabetes mellitus'/exp OR 'ketosis resistant diabetes mellitus' OR 'maturity onset diabetes'/exp OR 'maturity onset diabetes' OR 'maturity onset diabetes mellitus'/exp OR 'maturity onset diabetes mellitus' OR 'niddm'/exp OR 'niddm' OR 'niddm (non insulin dependent diabetes mellitus)'/exp OR 'niddm (non insulin dependent diabetes mellitus)' OR 'non insulin dependent (type 2) diabetes mellitus'/exp OR 'non insulin dependent (type 2) diabetes mellitus' OR 'non insulin dependent diabetes'/exp OR 'non insulin dependent diabetes' OR 'non-insulin-dependent diabetes mellitus'/exp OR 'non-insulin-dependent diabetes mellitus' OR 'noninsulin dependent (type 2) diabetes mellitus'/exp OR 'noninsulin dependent (type 2) diabetes mellitus' OR 'noninsulin dependent diabetes'/exp OR 'noninsulin dependent diabetes' OR 'noninsulin dependent diabetes mellitus'/exp OR 'noninsulin dependent diabetes mellitus' OR 't2dm'/exp OR 't2dm' OR 'tiidm'/exp OR 'tiidm' OR 'type 2 (insulin independent) diabetes'/exp OR 'type 2 (insulin independent) diabetes' OR 'type 2 diabetes'/exp OR 'type 2 diabetes' OR 'type 2 diabetes mellitus'/exp OR 'type 2 diabetes mellitus' OR 'type ii diabetes'/exp OR 'type ii diabetes' OR 'type ii diabetes mellitus'/exp OR 'type ii diabetes mellitus' OR 'non insulin dependent diabetes mellitus'/exp OR 'non insulin dependent diabetes mellitus' 515632  #2 'over weight' OR 'adipositas'/exp OR 'adipositas' OR 'adiposity'/exp OR 'adiposity' OR 'alimentary obesity'/exp OR 'alimentary obesity' OR 'body weight, excess'/exp OR 'body weight, excess' OR 'corpulency'/exp OR 'corpulency' OR 'fat overload syndrome'/exp OR 'fat overload syndrome' OR 'nutritional obesity'/exp OR 'nutritional obesity' OR 'obesitas'/exp OR 'obesitas' OR 'overweight'/exp OR 'overweight' OR 'obesity'/exp OR 'obesity' OR 'over-weight' OR 'pre-obese' OR 'excess weight'/exp OR 'excess weight' OR 'obese' OR 'obesity, abdominal'/exp OR 'obesity, abdominal' OR 'central obesity'/exp OR 'central obesity' OR 'visceral obesity'/exp OR 'visceral obesity' OR 'morbid obesity'/exp OR 'morbid obesity' OR 'severe obesity'/exp OR 'severe obesity' 1028409  #3 #1 AND #2 146838  #4 'intermittent fasting'/exp OR 'intermittent fasting' OR 'alternate-day fasting'/exp OR 'alternate-day fasting' OR 'alternate day fasting'/exp OR 'alternate day fasting' OR 'alternate-day diet' OR 'alternate day diet' OR 'adf' OR 'alternate day intermittent fasting' OR 'every other day fasting'/exp OR 'every other day fasting' OR 'every-other-day fasting'/exp OR 'every-other-day fasting' 13347  #5 'fasting'/exp OR 'fasting' OR 'short-term fasting' OR 'short term fasting'/exp OR 'short term fasting' OR 'short-term fast' OR 'short term fast' OR 'brief fasting' OR 'acute fasting' 254047  #6 'intermittent fasting'/exp OR 'intermittent fasting' OR 'time-restricted eating'/exp OR 'time-restricted eating' OR 'time restricted eating'/exp OR 'time restricted eating' OR 'time-restricted feeding'/exp OR 'time-restricted feeding' OR 'time restricted feeding'/exp OR 'time restricted feeding' OR 'tre' OR 'trf' OR 'time-limited eating' 32726  #7 'caloric restriction'/exp OR 'caloric restriction' OR 'continuous energy restriction'/exp OR 'continuous energy restriction' OR 'continuous calorie restriction' OR 'continuous caloric restriction' OR 'continuous energy restriction (cer)' OR 'daily calorie restriction' OR 'daily energy restriction' OR 'continuous dietary restriction' 24547  #8 #4 OR #5 OR #6 OR #7 308572  #9 #3 AND #8 24542  #10 #9 AND 'randomized controlled trial'/de 2701 |
|  |  |
|  |  |
|  |  |
|  |  |
|  |  |
|  |  |
|  |  |
|  |  |
|  |  |
|  |  |


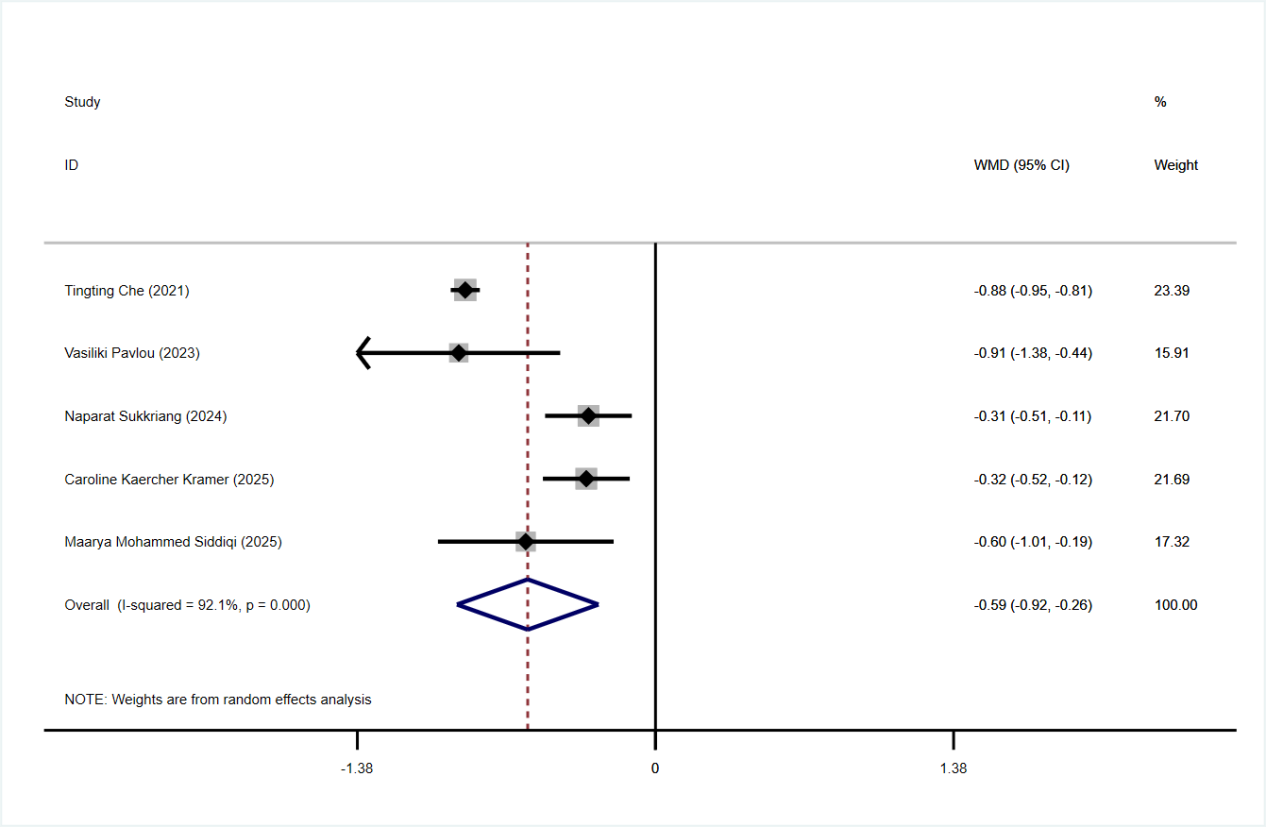


Supplementary Figure 1 Forest plot of TRE versus GD for HbA1c


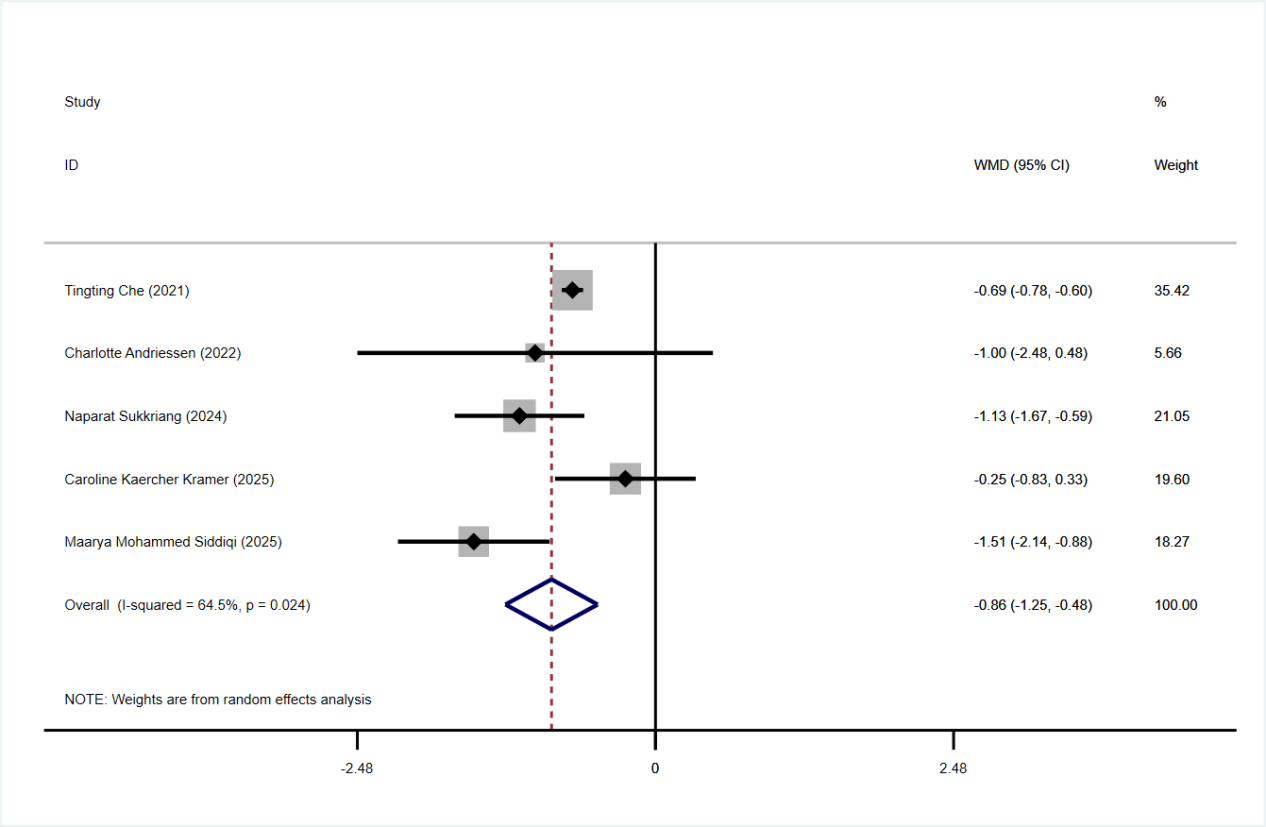


Supplementary Figure 2 Forest plot of TRE versus GD for FBG


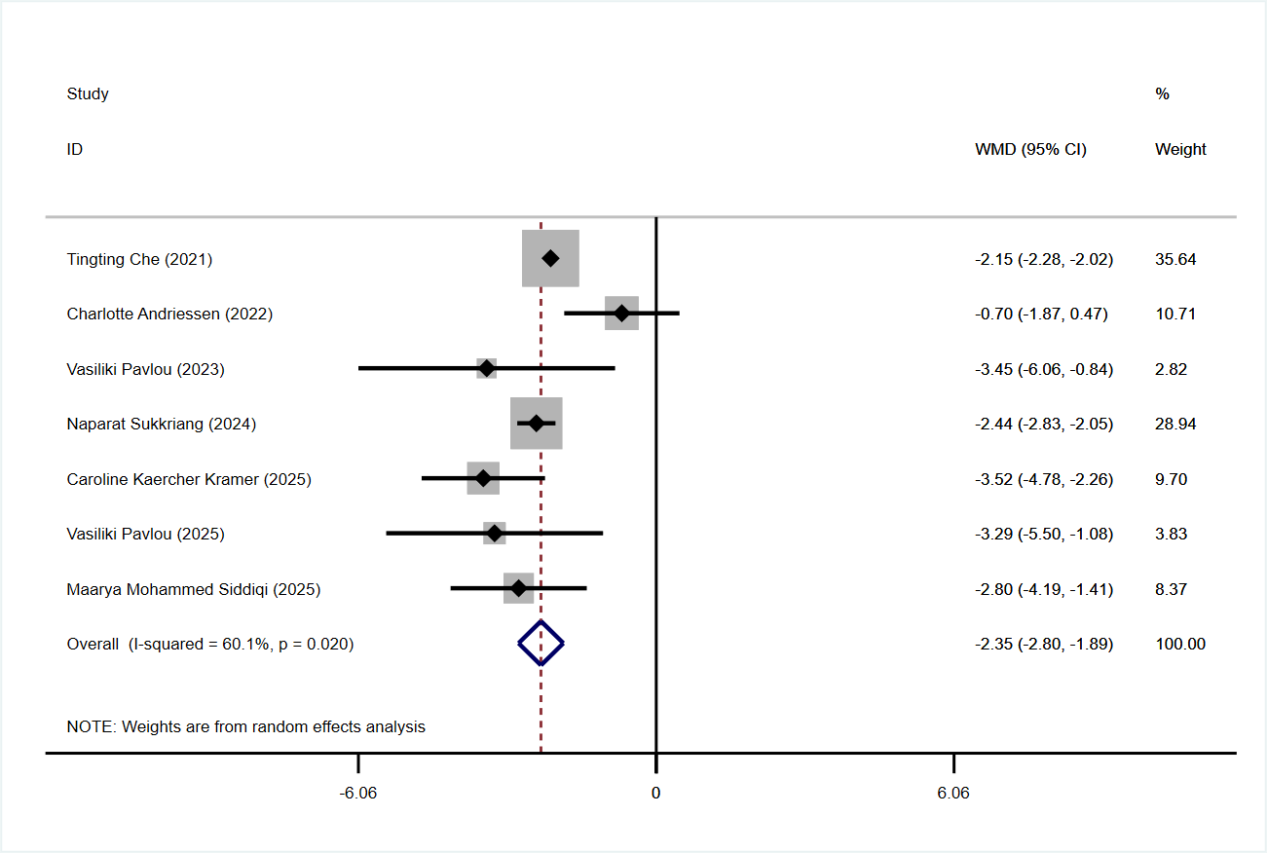


Supplementary Figure 3 Forest plot of TRE versus GD for Weight


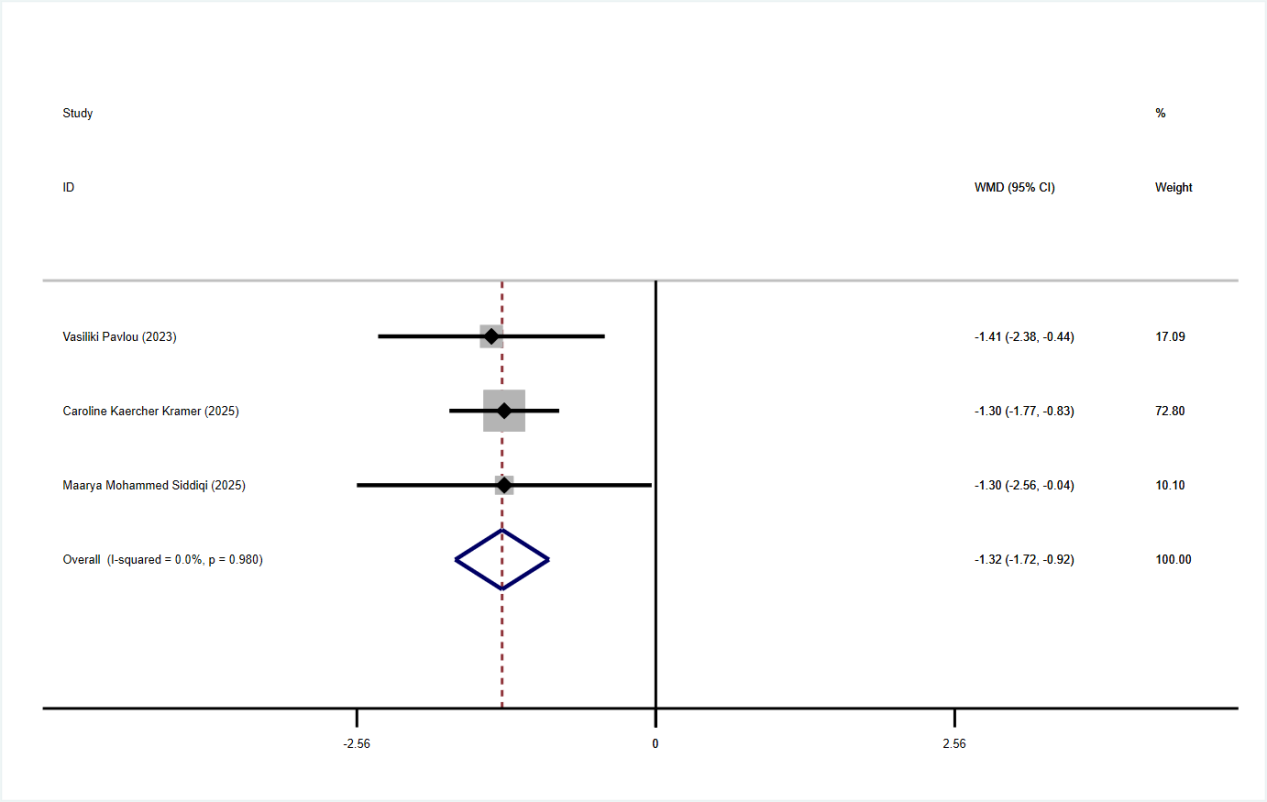


Supplementary Figure 4 Forest plot of TRE versus GD for BMI


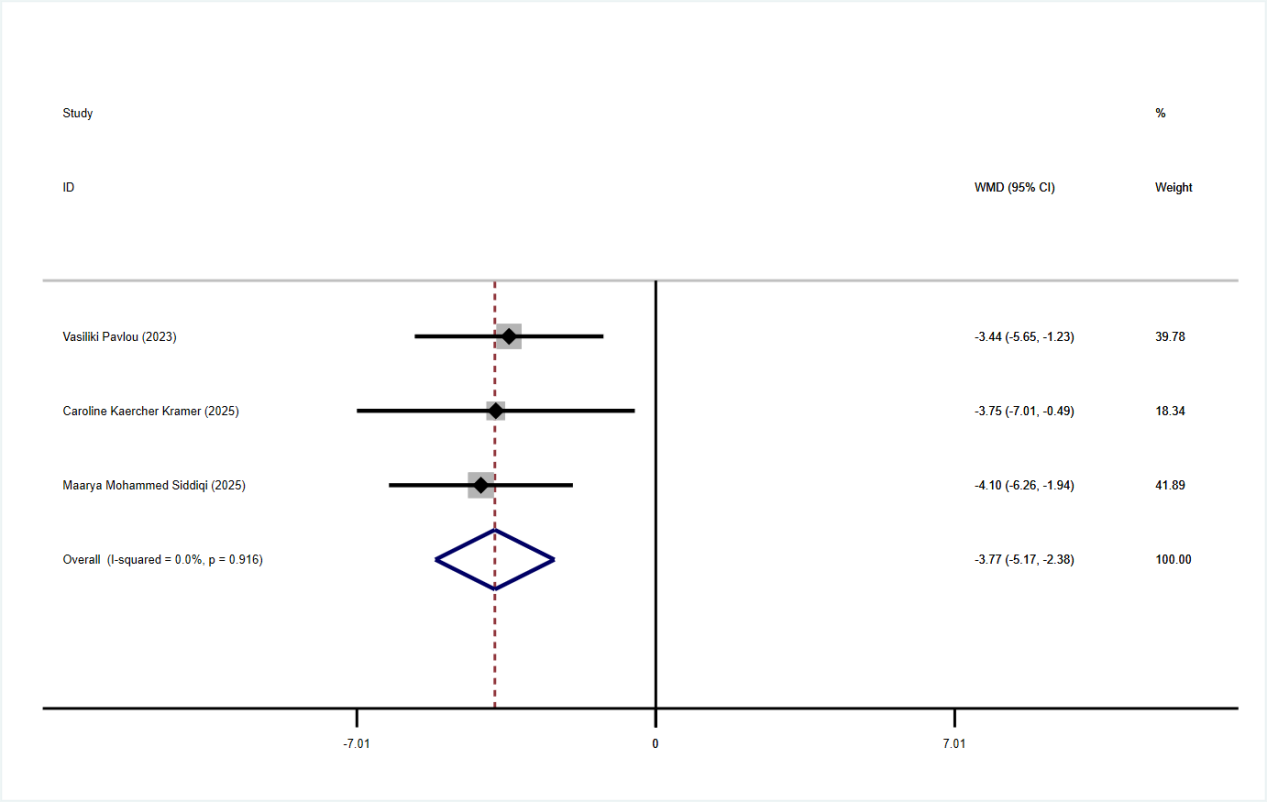


Supplementary Figure 5 Forest plot of TRE versus GD for WC


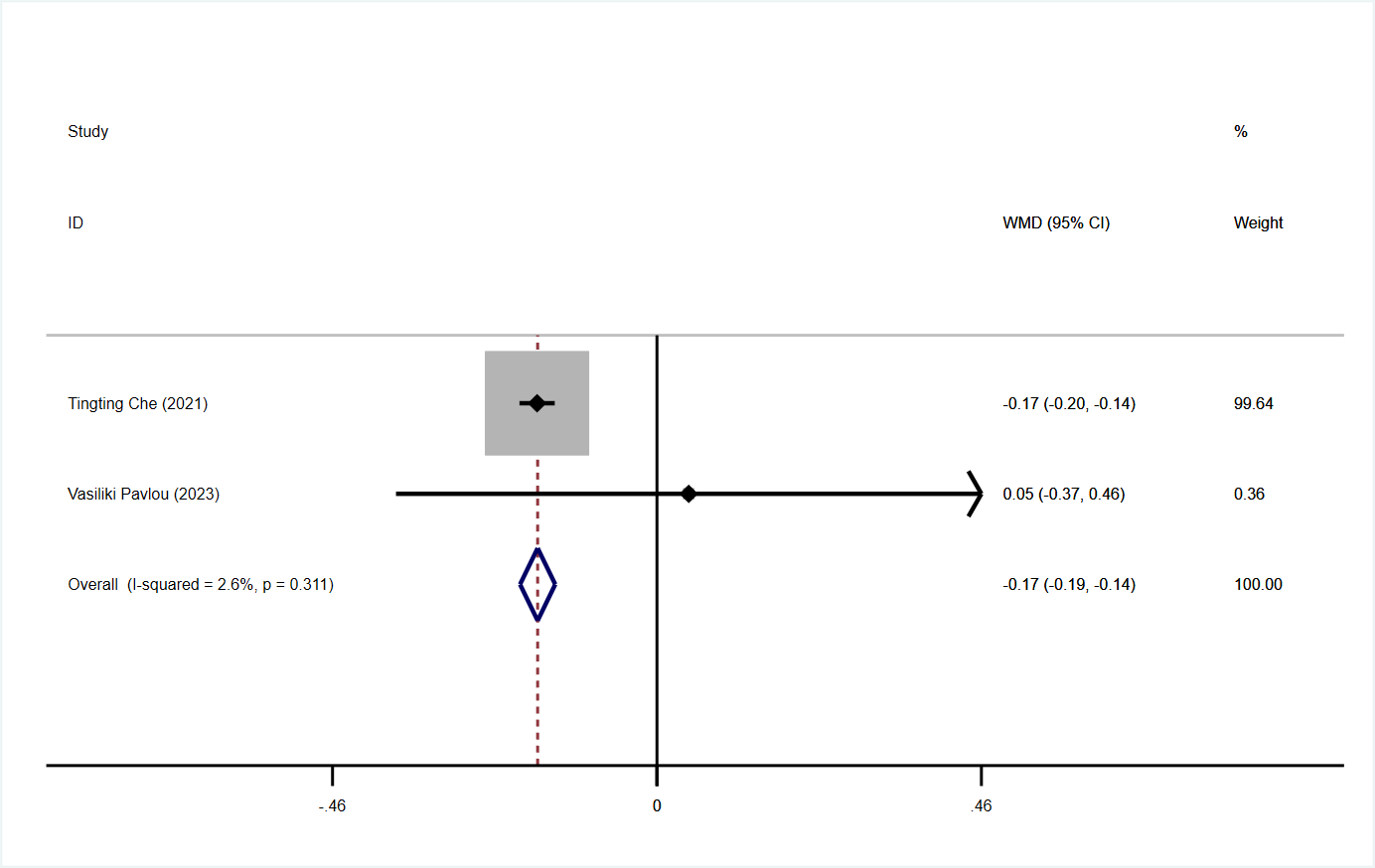


Supplementary Figure 6 Forest plot of TRE versus GD for TC


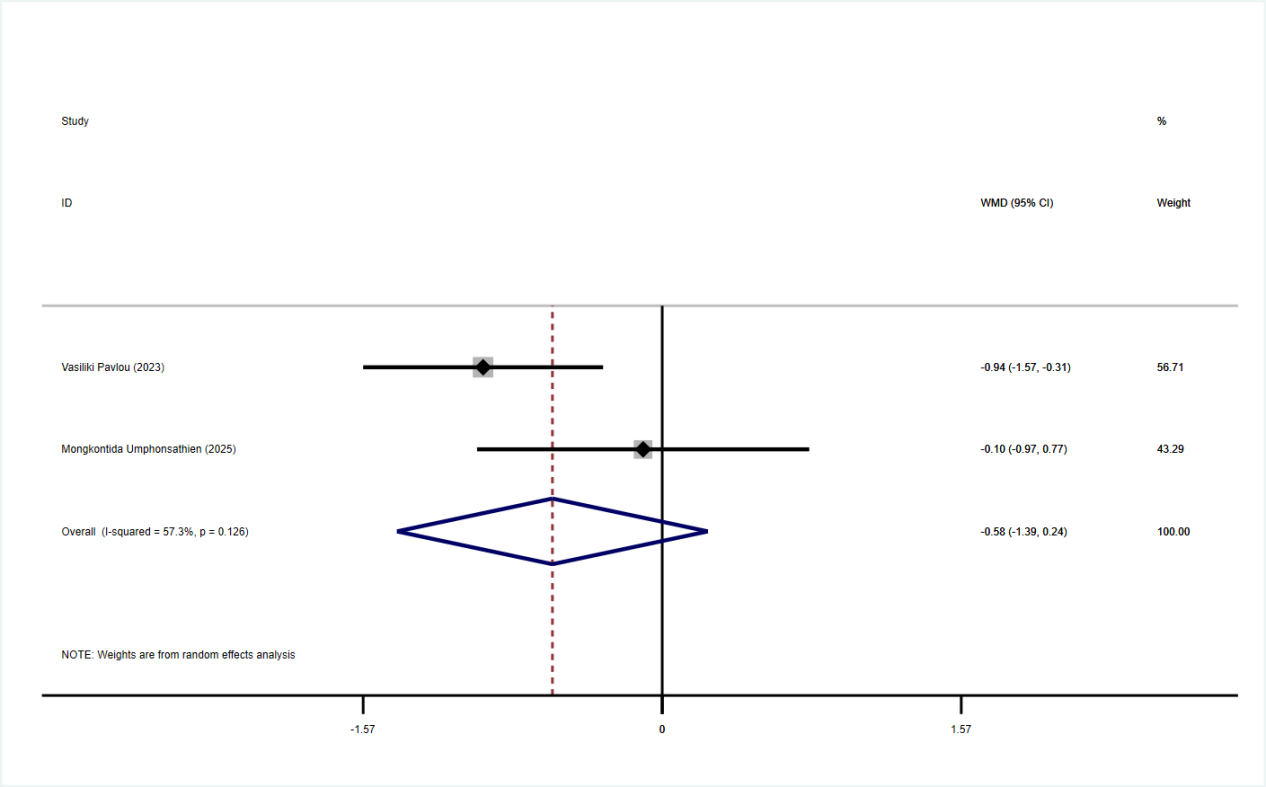


Supplementary Figure 7 Forest plot of CER versus GD for HbA1c


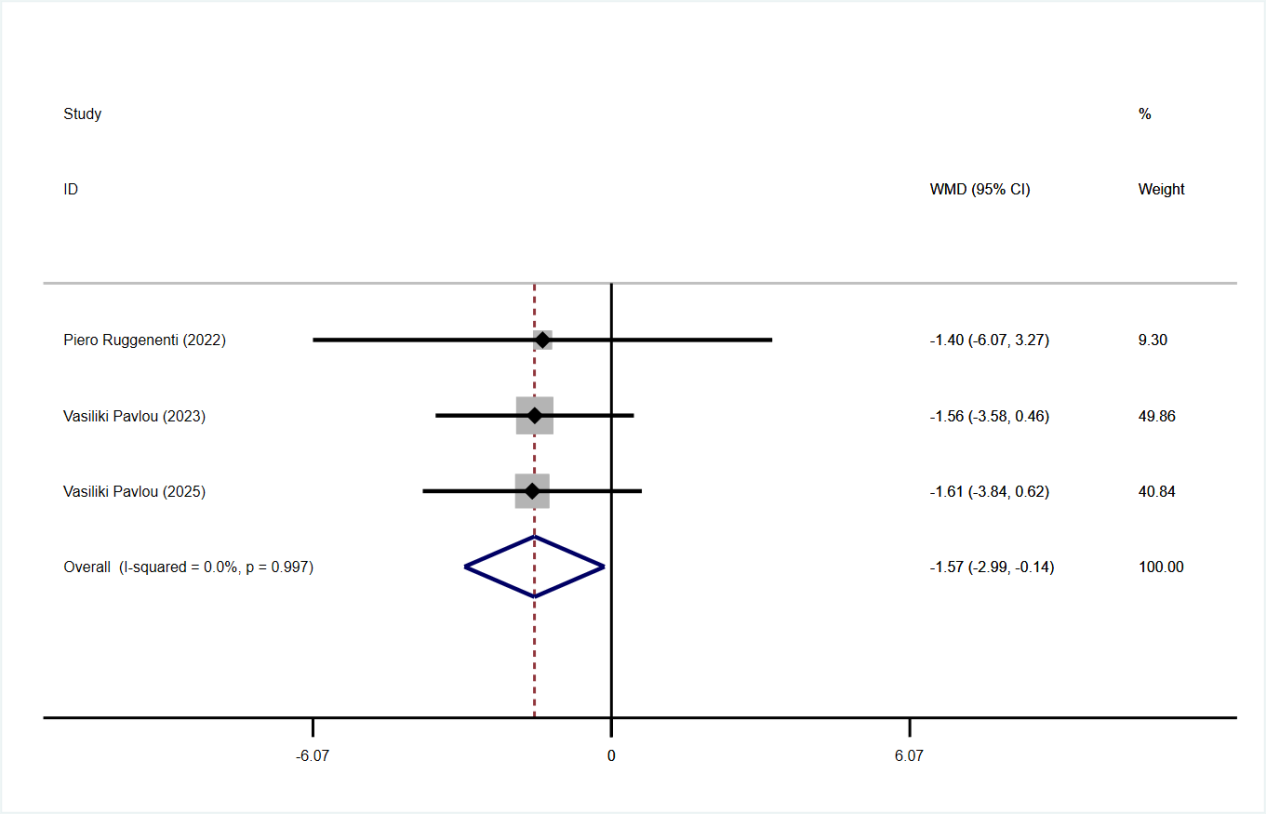


Supplementary Figure 8 Forest plot of CER versus GD for Weight


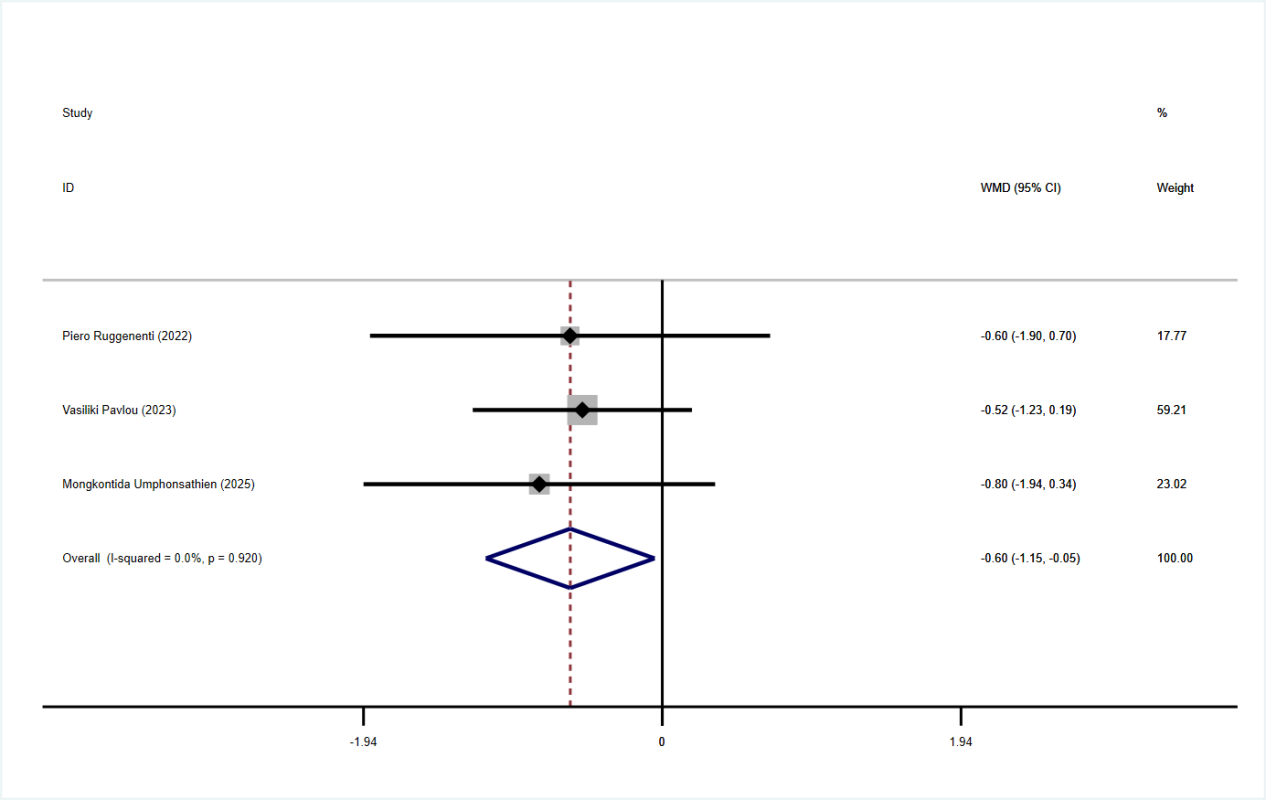


Supplementary Figure 9 Forest plot of CER versus GD for BMI


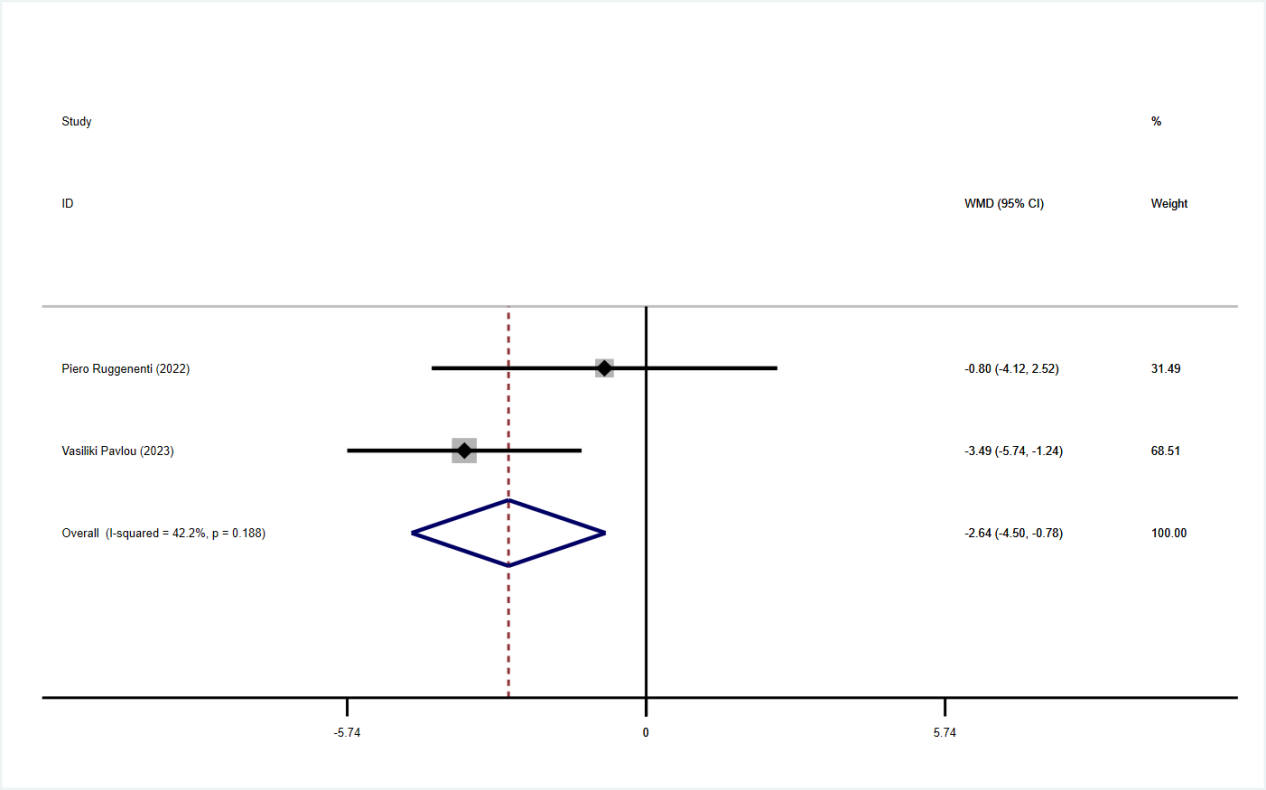


Supplementary Figure 10 Forest plot of CER versus GD for WC


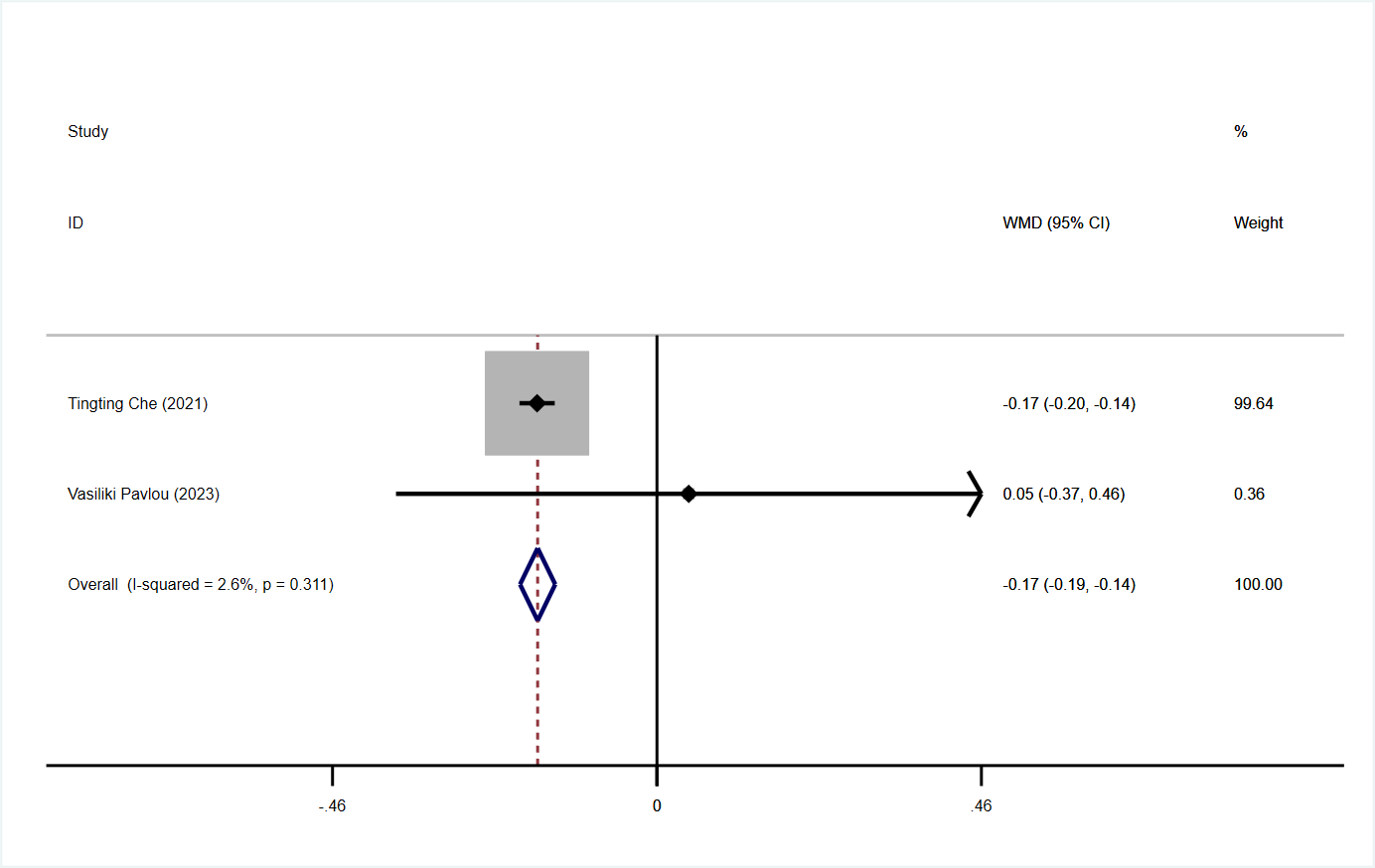


Supplementary Figure 11 Forest plot of CER versus GD for TC

Supplementary Table3. Global inconsistency table for HbA1c, FBG, Weight, BMI, WC and TC outcomes in overweight/obese individuals with type 2 diabetes

| **outcome indicator** | **P-value for global inconsistency analysis** |
| --- | --- |
| HbA1c | 0.6105 |
| FBG | 0.7256 |
| Weight | 0.6307 |
| BMI | 0.9691 |
| WC | 0.3661 |
| TC | 0.4222 |

Supplementary Table4. Node-splitting analysis of HbA1c results in overweight/obese individuals with type 2 diabetes

| **Side** | **Coef.** | **Std. Err.** | **Coef.** | **Std. Err.** | **Coef.** | **Std. Err.** | **P>z** | **tau** |
| --- | --- | --- | --- | --- | --- | --- | --- | --- |
| CER VS GD | 0.6126788 | 0.3095845 | 0.5069808 | 0.2274828 | 0.105698 | 0.3841101 | 0.783 | 0.2278121 |
| CER VS STF | 0.2 | 0.2576404 | 0.6843719 | 0.6062349 | -0.4843719 | 0.6587103 | 0.462 | 0.2194767 |
| CER VS TRE | 0.0233656 | 0.1757464 | -0.1490522 | 0.3732461 | 0.1724179 | 0.4046384 | 0.67 | 0.2294311 |
| GD VS STF | 0.1000057 | 0.5768937 | -0.3844149 | 0.3179664 | 0.4844206 | 0.6587173 | 0.462 | 0.2194767 |
| GD VS TRE | -0.5774815 | 0.1159041 | -0.1606552 | 0.4177784 | -0.4168263 | 0.4305343 | 0.333 | 0.2210153 |

Supplementary Table5. Node-splitting analysis of FBG results in overweight/obese individuals with type 2 diabetes

| **Side** | **Coef.** | **Std. Err.** | **Coef.** | **Std. Err.** | **Coef.** | **Std. Err.** | **P>z** | **tau** |
| --- | --- | --- | --- | --- | --- | --- | --- | --- |
| CER VS GD | 0.32 | 1.195302 | 0.7658108 | 0.4557758 | -0.4458109 | 1.27925 | 0.727 | 0.3427261 |
| CER VS TRE | -0.1 | 0.4076796 | -0.5431019 | 1.194746 | 0.4431019 | 1.262387 | 0.726 | 0.3426097 |
| GD VS TRE | -0.8670767 | 0.2056368 | -0.4187712 | 1.259623 | -0.4483054 | 1.276299 | 0.725 | 0.342806 |

Supplementary Table6. Node-splitting analysis of weight results in overweight/obese individuals with type 2 diabetes

| **Side** | **Coef.** | **Std. Err.** | **Coef.** | **Std. Err.** | **Coef.** | **Std. Err.** | **P>z** | **tau** |
| --- | --- | --- | --- | --- | --- | --- | --- | --- |
| CER VS GD | 1.739395 | 0.7857561 | 1.601898 | 1.069284 | 0.1374969 | 1.332021 | 0.918 | 0.7052192 |
| CER VS STF | 2.22e-11 | 2.068378 | 2.204911 | 6.693547 | -2.204911 | 7.005837 | 0.753 | 0.6297077 |
| CER VS TRE | -0.9045909 | 0.7317018 | 0.0947282 | 1.219402 | -0.9993191 | 1.447534 | 0.49 | 0.6638293 |
| GD VS STF | 0.5000011 | 6.665518 | -1.705021 | 2.157269 | 2.205022 | 7.005921 | 0.753 | 0.6297078 |
| GD VS TRE | -2.353668 | 0.3639488 | -1.470974 | 1.832742 | -0.8826941 | 1.862207 | 0.635 | 0.6564211 |

Supplementary Table7. Node-splitting analysis of BMI results in overweight/obese individuals with type 2 diabetes

| **Side** | **Coef.** | **Std. Err.** | **Coef.** | **Std. Err.** | **Coef.** | **Std. Err.** | **P>z** | **tau** |
| --- | --- | --- | --- | --- | --- | --- | --- | --- |
| CER VS GD | 0.5956796 | 0.27979 | 0.4363154 | 0.7602248 | 0.1593642 | 0.8207023 | 0.846 | 4.45e-10 |
| CER VS STF | 0.1000001 | 0.6726837 | 0.8902293 | 1.76393 | -0.7902292 | 1.887843 | 0.676 | 2.07e-10 |
| CER VS TRE | -0.8244297 | 0.426607 | -0.6474714 | 0.4584939 | -0.1769583 | 0.6534592 | 0.787 | 5.54e-11 |
| GD VS STF | 0.3000012 | 1.744465 | -0.4902706 | 0.721715 | 0.7902718 | 1.887864 | 0.676 | 1.10e-10 |
| GD VS TRE | -1.313833 | 0.2048344 | -1.401998 | 0.9469287 | 0.0881646 | 0.9585228 | 0.927 | 1.10e-09 |

Supplementary Table8. Node-splitting analysis of WC results in overweight/obese individuals with type 2 diabetes

| **Side** | **Coef.** | **Std. Err.** | **Coef.** | **Std. Err.** | **Coef.** | **Std. Err.** | **P>z** | **tau** |
| --- | --- | --- | --- | --- | --- | --- | --- | --- |
| CER VS GD | 2.642759 | 0.9500369 | 4.172123 | 2.89052 | -1.529364 | 2.890656 | 0.597 | 5.05e-07 |
| CER VS TRE | 0.0367199 | 1.390162 | -2.571715 | 1.765811 | 2.608435 | 2.241758 | 0.245 | 2.65e-10 |
| GD VS STF | 1.8 | 5.084186 | -5.402241 | 968.0334 | 7.202241 | 968.041 | 0.994 | 1.40e-07 |
| GD VS TRE | -3.773312 | 0.7119352 | 1.778812 | 4.063342 | -5.552125 | 4.065486 | 0.172 | 3.11e-09 |

Supplementary Table9. Node-splitting analysis of TC results in overweight/obese individuals with type 2 diabetes

| **Side** | **Coef.** | **Std. Err.** | **Coef.** | **Std. Err.** | **Coef.** | **Std. Err.** | **P>z** | **tau** |
| --- | --- | --- | --- | --- | --- | --- | --- | --- |
| CER VS GD | 0.0322413 | 0.1490993 | 0.2167041 | 0.3723267 | -0.1844629 | 0.4154879 | 0.657 | 0.0764703 |
| CER VS STF | 0.33 | 0.3053722 | 0.592322 | 0.3480789 | -0.262322 | 0.4630455 | 0.571 | 0.1004711 |
| CER VS TRE | 0.2065677 | 0.2218148 | -0.2103665 | 0.1396174 | 0.4169341 | 0.2541389 | 0.101 | 7.10e-08 |
| GD VS STF | 0.5000003 | 0.3157284 | 0.2376408 | 0.3387206 | 0.2623595 | 0.4630507 | 0.571 | 0.1004722 |
| GD VS TRE | -0.1692215 | 0.0127339 | 0.4941791 | 0.5146194 | -0.6634006 | 0.514857 | 0.198 | 1.87e-09 |

Supplementary Table10. Loop inconsistency plot for the HbA1c outcomes in overweight/obese individuals with type 2 diabetes

| **Loop** | **IF** | **seIF** | **z_value** | **p_value** | **CI_95** | **Loop_Heterog_tau2** |
| --- | --- | --- | --- | --- | --- | --- |
| CER-GD-STF | 0.552 | 0.679 | 0.813 | 0.416 | (0.00,1.88) | 0 |
| CER-GD-TRE | 0.028 | 0.384 | 0.072 | 0.943 | (0.00,0.78) | 0.056 |

Supplementary Table11. Loop inconsistency plot for the FBG outcomes in overweight/obese individuals with type 2 diabetes

| **Loop** | **IF** | **seIF** | **z_value** | **p_value** | **CI_95** | **Loop_Heterog_tau2** |
| --- | --- | --- | --- | --- | --- | --- |
| CER-GD-TRE | 0.925 | 1.322 | 0.7 | 0.484 | (0.00,3.52) | 0.82 |

Supplementary Table12. Loop inconsistency plot for the Weight outcomes in overweight/obese individuals with type 2 diabetes

| **Loop** | **IF** | **seIF** | **z_value** | **p_value** | **CI_95** | **Loop_Heterog_tau2** |
| --- | --- | --- | --- | --- | --- | --- |
| CER-GD-STF | 2.233 | 6.955 | 0.321 | 0.748 | (0.00,15.86) | 0 |
| CER-GD-TRE | 0.461 | 1.146 | 0.402 | 0.688 | (0.00,2.71) | 0.472 |

Supplementary Table13. Loop inconsistency plot for the BMI outcomes in overweight/obese individuals with type 2 diabetes

| **Loop** | **IF** | **seIF** | **z_value** | **p_value** | **CI_95** | **Loop_Heterog_tau2** |
| --- | --- | --- | --- | --- | --- | --- |
| CER-GD-STF | 0.799 | 1.891 | 0.422 | 0.673 | (0.00,4.50) | 0 |
| CER-GD-TRE | 0.113 | 0.57 | 0.199 | 0.842 | (0.00,1.23) | 0 |

Supplementary Table14. Loop inconsistency plot for the WC outcomes in overweight/obese individuals with type 2 diabetes

| **Loop** | **IF** | **seIF** | **z_value** | **p_value** | **CI_95** | **Loop_Heterog_tau2** |
| --- | --- | --- | --- | --- | --- | --- |
| CER-GD-TRE | 1.401 | 1.918 | 0.73 | 0.465 | (0.00,5.16) | 0 |

Supplementary Table15. Loop inconsistency plot for the TC outcomes in overweight/obese individuals with type 2 diabetes

| **Loop** | **IF** | **seIF** | **z_value** | **p_value** | **CI_95** | **Loop_Heterog_tau2** |
| --- | --- | --- | --- | --- | --- | --- |
| CER-GD-TRE | 0.343 | 0.263 | 1.307 | 0.191 | (0.00,0.86) | 0 |
| CER-GD-STF | 0.202 | 0.437 | 0.462 | 0.644 | (0.00,1.06) | 0 |

Supplementary Table16. Assessment Table of Heterogeneity (τ Values) for Clinical Outcome Measures in Overweight/Obese Patients with Type 2 Diabetes

| **outcome indicator** | **Heteroscedasticity τ-value** |
| --- | --- |
| HbA1c | 0.23567115 |
| FBG | 0.34261017 |
| Weight | 0.65163319 |
| BMI | 6.229e-10 |
| WC | 2.850e-09 |

Supplementary Table17. SUCRA ranking table for the HbA1c outcome in overweight/obese individuals with type 2 diabetes

| **Treatm~t** | **SUCRA** | **PrBest** | **MeanRank** |
| --- | --- | --- | --- |
| CER | 78.9 | 43.3 | 1.6 |
| GD | 5.8 | 0 | 3.8 |
| STF | 36.3 | 7.9 | 2.9 |
| TRE | 79 | 48.9 | 1.6 |

Supplementary Table18. SUCRA ranking table for the FBG outcome in overweight/obese individuals with type 2 diabetes

| **Treatm~t** | **SUCRA** | **PrBest** | **MeanRank** |
| --- | --- | --- | --- |
| CER | 66 | 24.4 | 2 |
| GD | 11.6 | 0 | 3.7 |
| STF | 43.3 | 22.3 | 2.7 |
| TRE | 79.1 | 53.4 | 1.6 |

Supplementary Table19.SUCRA ranking table for the Weight outcome in overweight/obese individuals with type 2 diabetes

| **Treatm~t** | **SUCRA** | **PrBest** | **MeanRank** |
| --- | --- | --- | --- |
| CER | 56.8 | 8.7 | 2.3 |
| GD | 8 | 0 | 3.8 |
| STF | 51.1 | 31.6 | 2.5 |
| TRE | 84 | 59.7 | 1.5 |

Supplementary Table20. SUCRA ranking table for the BMI outcome in overweight/obese individuals with type 2 diabetes

| **Treatm~t** | **SUCRA** | **PrBest** | **MeanRank** |
| --- | --- | --- | --- |
| CER | 54.6 | 0.6 | 2.4 |
| GD | 10.1 | 0 | 3.7 |
| STF | 38.3 | 8.2 | 2.9 |
| TRE | 97 | 91.3 | 1.1 |

Supplementary Table21.SUCRA ranking table for the WC outcome in overweight/obese individuals with type 2 diabetes

| **Treatm~t** | **SUCRA** | **PrBest** | **MeanRank** |
| --- | --- | --- | --- |
| CER | 67.2 | 17 | 2 |
| GD | 21.9 | 0 | 3.3 |
| STF | 22 | 12.8 | 3.3 |
| TRE | 89 | 70.1 | 1.3 |

Supplementary Table22.SUCRA ranking table for the TC outcome in overweight/obese individuals with type 2 diabetes

| **Treatm~t** | **SUCRA** | **PrBest** | **MeanRank** |
| --- | --- | --- | --- |
| CER | 64.2 | 21.9 | 2.1 |
| GD | 41 | 0 | 2.8 |
| STF | 2.3 | 0.5 | 3.9 |
| TRE | 92.4 | 77.6 | 1.2 |

Supplementary Table23.Meta-regression analysis of HbA1c outcomes in overweight/obese patients with type 2 diabetes, with HbA1c, BMI, duration of intervention, age and country as moderator variables

| **Intervention** | **Covariate** | **Coefficient** | **Standard Error** | **Z-statistic** | **P>z** | **lower confidence interval** | **upper confidence interval** |
| --- | --- | --- | --- | --- | --- | --- | --- |
| CER VS GD |  |  |  |  |  |  |  |
|  | HbA1c | 0.2611301 | 0.2460725 | 1.06 | 0.289 | -0.2211631 | 0.7434233 |
|  | Cons | -1.455879 | 1.831151 | -0.8 | 0.427 | -5.044869 | 2.133112 |
| CER VS STF |  |  |  |  |  |  |  |
|  | HbA1c | 0.6393879 | 0.6957384 | 0.92 | 0.358 | -0.7242343 | 2.00301 |
|  | Cons | -4.504806 | 5.20562 | -0.87 | 0.387 | -14.70763 | 5.698022 |
| CER VS TRE |  |  |  |  |  |  |  |
|  | HbA1c | -0.0467034 | 0.2150937 | -0.22 | 0.828 | -0.4682792 | 0.3748725 |
|  | Cons | 0.3723017 | 1.570444 | 0.24 | 0.813 | -2.705712 | 3.450315 |
| CER VS GD |  |  |  |  |  |  |  |
|  | BMI | -0.0040393 | 0.0525624 | -0.08 | 0.939 | -0.1070596 | 0.0989811 |
|  | Cons | 0.6861606 | 1.753189 | 0.39 | 0.696 | -2.750026 | 4.122348 |
| CER VS STF |  |  |  |  |  |  |  |
|  | BMI | 0.3264753 | 0.6003618 | 0.54 | 0.587 | -0.8502122 | 1.503163 |
|  | Cons | -11.43832 | 21.52766 | -0.53 | 0.595 | -53.63176 | 30.75512 |
| CER VS TRE |  |  |  |  |  |  |  |
|  | BMI | 0.007803 | 0.0451796 | 0.17 | 0.863 | -0.0807473 | 0.0963533 |
|  | Cons | -0.2381322 | 1.516152 | -0.16 | 0.875 | -3.209735 | 2.733471 |
| CER VS GD |  |  |  |  |  |  |  |
|  | Duration of intervention | 0.0302666 | 0.0317435 | 0.95 | 0.34 | -0.0319495 | 0.0924827 |
|  | Cons | 0.0826425 | 0.5711484 | 0.14 | 0.885 | -1.036788 | 1.202073 |
| CER VS STF |  |  |  |  |  |  |  |
|  | Duration of intervention | -0.0025376 | 0.0143435 | -0.18 | 0.86 | -0.0306504 | 0.0255752 |
|  | Cons | 0.3218062 | 0.4332889 | 0.74 | 0.458 | -0.5274244 | 1.171037 |
| CER VS TRE |  |  |  |  |  |  |  |
|  | Duration of intervention | 0.0083631 | 0.0279438 | 0.3 | 0.765 | -0.0464059 | 0.063132 |
|  | Cons | -0.1631771 | 0.5284746 | -0.31 | 0.757 | -1.198968 | 0.8726141 |
| CER VS GD |  |  |  |  |  |  |  |
|  | Age | 0.0214531 | 0.0374899 | 0.57 | 0.567 | -0.0520258 | 0.094932 |
|  | Cons | -0.64031 | 2.06173 | -0.31 | 0.756 | -4.681227 | 3.400607 |
| CER VS STF |  |  |  |  |  |  |  |
|  | Age | -0.3330473 | 0.4751809 | -0.7 | 0.483 | -1.264385 | 0.5982901 |
|  | Cons | 20.6267 | 29.02948 | 0.71 | 0.477 | -36.27003 | 77.52344 |
| CER VS TRE |  |  |  |  |  |  |  |
|  | Age | 0.0162971 | 0.0267559 | 0.61 | 0.542 | -0.0361436 | 0.0687377 |
|  | Cons | -0.9463133 | 1.587497 | -0.6 | 0.551 | -4.057751 | 2.165124 |
| CER VS GD |  |  |  |  |  |  |  |
|  | Country | -0.0723556 | 0.0603858 | -1.2 | 0.231 | -0.1907096 | 0.0459985 |
|  | Cons | 0.9751812 | 0.3050611 | 3.2 | 0.001 | 0.3772724 | 1.57309 |
| CER VS STF |  |  |  |  |  |  |  |
|  | Country | 0.2928644 | 0.2914573 | 1 | 0.315 | -0.2783813 | 0.8641102 |
|  | Cons | -0.3857289 | 0.6805707 | -0.57 | 0.571 | -1.719623 | 0.9481652 |
| CER VS TRE |  |  |  |  |  |  |  |
|  | Country | 0.0064432 | 0.0597105 | 0.11 | 0.914 | -0.1105872 | 0.1234736 |
|  | Cons | 0.0168707 | 0.3026331 | 0.06 | 0.956 | -0.5762793 | 0.6100208 |

Supplementary Table24.Meta-regression analysis of FBG outcomes in overweight/obese patients with type 2 diabetes, with HbA1c, BMI, duration of intervention, age and country as moderator variables

| **Intervention** | **Covariate** | **Coefficient** | **Standard Error** | **Z-statistic** | **P>z** | **lower confidence interval** | **upper confidence interval** |
| --- | --- | --- | --- | --- | --- | --- | --- |
| CER VS GD |  |  |  |  |  |  |  |
|  | HbA1c | 0.1737086 | 0.9023887 | 0.19 | 0.847 | -1.594941 | 1.942358 |
|  | Cons | -0.6602978 | 6.484177 | -0.1 | 0.919 | -13.36905 | 12.04846 |
| CER VS STF |  |  |  |  |  |  |  |
|  | Cons | 0.3 | 0.7156548 | 0.42 | 0.675 | -1.102658 | 1.702658 |
| CER VS TRE |  |  |  |  |  |  |  |
|  | HbA1c | -0.0632327 | 0.8491343 | -0.07 | 0.941 | -1.727505 | 1.60104 |
|  | Cons | 0.3045622 | 6.033417 | 0.05 | 0.96 | -11.52072 | 12.12984 |
| CER VS GD |  |  |  |  |  |  |  |
|  | BMI | -0.0513432 | 0.2611249 | -0.2 | 0.844 | -0.5631386 | 0.4604522 |
|  | Cons | 2.273341 | 8.138137 | 0.28 | 0.78 | -13.67712 | 18.2238 |
| CER VS STF |  |  |  |  |  |  |  |
|  | Cons | 0.3 | 0.752212 | 0.4 | 0.69 | -1.174308 | 1.774308 |
| CER VS TRE |  |  |  |  |  |  |  |
|  | BMI | -0.0170997 | 0.2476857 | -0.07 | 0.945 | -0.5025547 | 0.4683553 |
|  | Cons | 0.3885415 | 7.737626 | 0.05 | 0.96 | -14.77693 | 15.55401 |
| CER VS GD |  |  |  |  |  |  |  |
|  | Duration of intervention | 0.0637339 | 0.0600244 | 1.06 | 0.288 | -0.0539118 | 0.1813795 |
|  | Cons | -0.1601868 | 1.017865 | -0.16 | 0.875 | -2.155165 | 1.834792 |
| CER VS STF |  |  |  |  |  |  |  |
|  | Cons | 0.3 | 0.5923347 | 0.51 | 0.613 | -0.8609548 | 1.460955 |
| CER VS TRE |  |  |  |  |  |  |  |
|  | Duration of intervention | 0.003422 | 0.0551153 | 0.06 | 0.95 | -0.1046019 | 0.111446 |
|  | Cons | -0.1822031 | 0.9643501 | -0.19 | 0.85 | -2.072295 | 1.707888 |
| CER VS GD |  |  |  |  |  |  |  |
|  | Age | 0.0128577 | 0.0688382 | 0.19 | 0.852 | -0.1220626 | 0.147778 |
|  | Cons | -0.068694 | 4.018382 | -0.02 | 0.986 | -7.944579 | 7.807191 |
| CER VS STF |  |  |  |  |  |  |  |
|  | Cons | 0.3 | 0.7211971 | 0.42 | 0.677 | -1.11352 | 1.71352 |
| CER VS TRE |  |  |  |  |  |  |  |
|  | Age | 0.0137336 | 0.0604665 | 0.23 | 0.82 | -0.1047785 | 0.1322457 |
|  | Cons | -0.9845726 | 3.68541 | -0.27 | 0.789 | -8.207844 | 6.238699 |
| CER VS GD |  |  |  |  |  |  |  |
|  | Country | 0.0076752 | 0.2153737 | 0.04 | 0.972 | -0.4144496 | 0.4298 |
|  | Cons | 0.6246598 | 1.129168 | 0.55 | 0.58 | -1.58847 | 2.837789 |
| CER VS STF |  |  |  |  |  |  |  |
|  | Cons | 0.3 | 0.7309675 | 0.41 | 0.682 | -1.13267 | 1.73267 |
| CER VS TRE |  |  |  |  |  |  |  |
|  | Country | -0.033595 | 0.2044834 | -0.16 | 0.87 | -0.434375 | 0.3671851 |
|  | Cons | -0.0085606 | 1.016226 | -0.01 | 0.993 | -2.000328 | 1.983207 |

Supplementary Table25.Meta-regression analysis of Weight outcomes in overweight/obese patients with type 2 diabetes, with HbA1c, BMI, duration of intervention, age and country as moderator variables

| **Intervention** | **Covariate** | **Coefficient** | **Standard Error** | **Z-statistic** | **P>z** | **lower confidence interval** | **upper confidence interval** |
| --- | --- | --- | --- | --- | --- | --- | --- |
| CER VS GD |  |  |  |  |  |  |  |
|  | HbA1c | -0.5468305 | 1.023608 | -0.53 | 0.593 | -2.553066 | 1.459405 |
|  | Cons | 5.886092 | 7.907437 | 0.74 | 0.457 | -9.6122 | 21.38438 |
| CER VS STF |  |  |  |  |  |  |  |
|  | HbA1c | 1.847314 | 7.072375 | 0.26 | 0.794 | -12.01429 | 15.70891 |
|  | Cons | -13.48539 | 52.30725 | -0.26 | 0.797 | -116.0057 | 89.03493 |
| CER VS TRE |  |  |  |  |  |  |  |
|  | HbA1c | -0.8581898 | 0.9623793 | -0.89 | 0.373 | -2.744419 | 1.028039 |
|  | Cons | 5.850092 | 7.348937 | 0.8 | 0.426 | -8.55356 | 20.25374 |
| CER VS GD |  |  |  |  |  |  |  |
|  | BMI | -0.0970516 | 0.1330954 | -0.73 | 0.466 | -0.3579138 | 0.1638106 |
|  | Cons | 5.287681 | 4.664815 | 1.13 | 0.257 | -3.855189 | 14.43055 |
| CER VS STF |  |  |  |  |  |  |  |
|  | BMI | 3.179686 | 10.01839 | 0.32 | 0.751 | -16.456 | 22.81538 |
|  | Cons | -114.4687 | 361.2801 | -0.32 | 0.751 | -822.5646 | 593.6273 |
| CER VS TRE |  |  |  |  |  |  |  |
|  | BMI | -0.2071836 | 0.1348136 | -1.54 | 0.124 | -0.4714133 | 0.0570462 |
|  | Cons | 6.350827 | 4.671578 | 1.36 | 0.174 | -2.805298 | 15.50695 |
| CER VS GD |  |  |  |  |  |  |  |
|  | Duration of intervention | -0.0144277 | 0.0318712 | -0.45 | 0.651 | -0.076894 | 0.0480386 |
|  | Cons | 2.37706 | 1.009768 | 2.35 | 0.019 | 0.3979519 | 4.356169 |
| CER VS STF |  |  |  |  |  |  |  |
|  | Duration of intervention | -0.0751055 | 0.1944397 | -0.39 | 0.699 | -0.4562003 | 0.3059893 |
|  | Cons | 3.605065 | 8.955156 | 0.4 | 0.687 | -13.94672 | 21.15685 |
| CER VS TRE |  |  |  |  |  |  |  |
|  | Duration of intervention | -0.1032273 | 0.0620953 | -1.66 | 0.096 | -0.2249319 | 0.0184772 |
|  | Cons | 1.048737 | 1.242959 | 0.84 | 0.399 | -1.387418 | 3.484893 |
| CER VS GD |  |  |  |  |  |  |  |
|  | Age | 0.0515518 | 0.1227945 | 0.42 | 0.675 | -0.189121 | 0.2922246 |
|  | Cons | -1.525293 | 7.172772 | -0.21 | 0.832 | -15.58367 | 12.53308 |
| CER VS STF |  |  |  |  |  |  |  |
|  | Age | -1.87506 | 6.386057 | -0.29 | 0.769 | -14.3915 | 10.64138 |
|  | Cons | 114.3786 | 388.9354 | 0.29 | 0.769 | -647.9207 | 876.678 |
| CER VS TRE |  |  |  |  |  |  |  |
|  | Age | 0.101362 | 0.1133504 | 0.89 | 0.371 | -0.1208006 | 0.3235247 |
|  | Cons | -6.539446 | 6.741238 | -0.97 | 0.332 | -19.75203 | 6.673138 |
| CER VS GD |  |  |  |  |  |  |  |
|  | Country | 0.336852 | 0.3068083 | 1.1 | 0.272 | -0.2644812 | 0.9381853 |
|  | Cons | 0.1817543 | 1.529373 | 0.12 | 0.905 | -2.815761 | 3.17927 |
| CER VS STF |  |  |  |  |  |  |  |
|  | Country | 1.014534 | 3.515627 | 0.29 | 0.773 | -5.875968 | 7.905036 |
|  | Cons | -2.029068 | 7.919191 | -0.26 | 0.798 | -17.5504 | 13.49226 |
| CER VS TRE |  |  |  |  |  |  |  |
|  | Country | 0.2743592 | 0.3184033 | 0.86 | 0.389 | -0.3496998 | 0.8984182 |
|  | Cons | -1.859374 | 1.571147 | -1.18 | 0.237 | -4.938765 | 1.220018 |

Supplementary Table26.Meta-regression analysis of BMI outcomes in overweight/obese patients with type 2 diabetes, with HbA1c, BMI, duration of intervention, age and country as moderator variables

| **Intervention** | **Covariate** | **Coefficient** | **Standard Error** | **Z-statistic** | **P>z** | **lower confidence interval** | **upper confidence interval** |
| --- | --- | --- | --- | --- | --- | --- | --- |
| CER VS GD |  |  |  |  |  |  |  |
|  | HbA1c | -0.2341257 | 0.6527477 | -0.36 | 0.72 | -1.513488 | 1.045236 |
|  | Cons | 2.422398 | 5.078346 | 0.48 | 0.633 | -7.530978 | 12.37577 |
| CER VS STF |  |  |  |  |  |  |  |
|  | HbA1c | 0.6791232 | 1.920666 | 0.35 | 0.724 | -3.085312 | 4.443558 |
|  | Cons | -4.857599 | 14.27037 | -0.34 | 0.734 | -32.82702 | 23.11182 |
| CER VS TRE |  |  |  |  |  |  |  |
|  | HbA1c | -0.2716449 | 0.6692746 | -0.41 | 0.685 | -1.583399 | 1.040109 |
|  | Cons | 1.375045 | 5.198985 | 0.26 | 0.791 | -8.814779 | 11.56487 |
| CER VS GD |  |  |  |  |  |  |  |
|  | BMI | -0.034948 | 0.0840204 | -0.42 | 0.677 | -0.199625 | 0.129729 |
|  | Cons | 1.880291 | 3.055846 | 0.62 | 0.538 | -4.109056 | 7.869638 |
| CER VS STF |  |  |  |  |  |  |  |
|  | BMI | 1.139516 | 2.699855 | 0.42 | 0.673 | -4.152101 | 6.431134 |
|  | Cons | -40.92259 | 97.43622 | -0.42 | 0.674 | -231.8941 | 150.0489 |
| CER VS TRE |  |  |  |  |  |  |  |
|  | BMI | -0.0485553 | 0.0956137 | -0.51 | 0.612 | -0.2359547 | 0.1388441 |
|  | Cons | 1.020917 | 3.437447 | 0.3 | 0.766 | -5.716356 | 7.758191 |
| CER VS GD |  |  |  |  |  |  |  |
|  | Duration of intervention | -0.0006571 | 0.0095498 | -0.07 | 0.945 | -0.0193743 | 0.0180601 |
|  | Cons | 0.6231907 | 0.4195634 | 1.49 | 0.137 | -0.1991385 | 1.44552 |
| CER VS STF |  |  |  |  |  |  |  |
|  | Duration of intervention | -0.0226463 | 0.0527888 | -0.43 | 0.668 | -0.1261104 | 0.0808178 |
|  | Cons | 1.187022 | 2.380396 | 0.5 | 0.618 | -3.478468 | 5.852513 |
| CER VS TRE |  |  |  |  |  |  |  |
|  | Duration of intervention | -0.0044335 | 0.0273643 | -0.16 | 0.871 | -0.0580665 | 0.0491994 |
|  | Cons | -0.6541445 | 0.5701925 | -1.15 | 0.251 | -1.771701 | 0.4634122 |
| CER VS GD |  |  |  |  |  |  |  |
|  | Age | -0.0118456 | 0.0559193 | -0.21 | 0.832 | -0.1214453 | 0.0977542 |
|  | Cons | 1.269016 | 3.089286 | 0.41 | 0.681 | -4.785872 | 7.323905 |
| CER VS STF |  |  |  |  |  |  |  |
|  | Age | -0.6903915 | 1.7357 | -0.4 | 0.691 | -4.092301 | 2.711518 |
|  | Cons | 42.21388 | 105.6426 | 0.4 | 0.689 | -164.8418 | 249.2696 |
| CER VS TRE |  |  |  |  |  |  |  |
|  | Age | 0.1133427 | 0.4128295 | 0.27 | 0.784 | -0.6957882 | 0.9224735 |
|  | Cons | -7.066454 | 23.04557 | -0.31 | 0.759 | -52.23493 | 38.10203 |
| CER VS GD |  |  |  |  |  |  |  |
|  | Country | 0.0461281 | 0.1552683 | 0.3 | 0.766 | -0.2581922 | 0.3504485 |
|  | Cons | 0.4011183 | 0.7431399 | 0.54 | 0.589 | -1.055409 | 1.857646 |
| CER VS STF |  |  |  |  |  |  |  |
|  | Country | 0.3927967 | 0.9457955 | 0.42 | 0.678 | -1.460928 | 2.246522 |
|  | Cons | -0.6855934 | 2.221627 | -0.31 | 0.758 | -5.039902 | 3.668716 |
| CER VS TRE |  |  |  |  |  |  |  |
|  | Country | 0.0522957 | 0.1578074 | 0.33 | 0.74 | -0.2570011 | 0.3615925 |
|  | Cons | -0.9526931 | 0.795137 | -1.2 | 0.231 | -2.511133 | 0.6057468 |

Supplementary Table27.Meta-regression analysis of WC outcomes in overweight/obese patients with type 2 diabetes, with HbA1c, BMI, duration of intervention, age and country as moderator variables

| **Intervention** | **Covariate** | **Coefficient** | **Standard Error** | **Z-statistic** | **P>z** | **lower confidence interval** | **upper confidence interval** |
| --- | --- | --- | --- | --- | --- | --- | --- |
| CER VS GD |  |  |  |  |  |  |  |
|  | HbA1c | 2.777951 | 2.03404 | 1.37 | 0.172 | -1.208694 | 6.764596 |
|  | Cons | -18.92292 | 15.87801 | -1.19 | 0.233 | -50.04324 | 12.19741 |
| CER VS STF |  |  |  |  |  |  |  |
|  | Cons | 5.933858 | 5.272109 | 1.13 | 0.26 | -4.399285 | 16.267 |
| CER VS TRE |  |  |  |  |  |  |  |
|  | HbA1c | 2.739844 | 2.323336 | 1.18 | 0.238 | -1.813811 | 7.293499 |
|  | Cons | -22.39718 | 18.25353 | -1.23 | 0.22 | -58.17344 | 13.37908 |
| CER VS GD |  |  |  |  |  |  |  |
|  | BMI | 0.3949397 | 0.3005912 | 1.31 | 0.189 | -0.1942081 | 0.9840876 |
|  | Cons | -11.91652 | 11.12121 | -1.07 | 0.284 | -33.71369 | 9.880651 |
| CER VS STF |  |  |  |  |  |  |  |
|  | Cons | 4.377621 | 5.172168 | 0.85 | 0.397 | -5.759642 | 14.51488 |
| CER VS TRE |  |  |  |  |  |  |  |
|  | BMI | 0.4572121 | 0.3214064 | 1.42 | 0.155 | -0.1727329 | 1.087157 |
|  | Cons | -17.77044 | 11.84354 | -1.5 | 0.134 | -40.98336 | 5.442476 |
| CER VS GD |  |  |  |  |  |  |  |
|  | Duration of intervention | -0.0385725 | 0.028258 | -1.37 | 0.172 | -0.0939572 | 0.0168122 |
|  | Cons | 4.50303 | 1.607067 | 2.8 | 0.005 | 1.353237 | 7.652824 |
| CER VS STF |  |  |  |  |  |  |  |
|  | Cons | 5.839947 | 5.259422 | 1.11 | 0.267 | -4.46833 | 16.14823 |
| CER VS TRE |  |  |  |  |  |  |  |
|  | Duration of intervention | -0.0401467 | 0.1071984 | -0.37 | 0.708 | -0.2502517 | 0.1699583 |
|  | Cons | 0.762303 | 2.776159 | 0.27 | 0.784 | -4.678868 | 6.203474 |
| CER VS GD |  |  |  |  |  |  |  |
|  | Age | -0.3027288 | 0.2298038 | -1.32 | 0.188 | -0.753136 | 0.1476784 |
|  | Cons | 20.14433 | 13.31707 | 1.51 | 0.13 | -5.956661 | 46.24531 |
| CER VS STF |  |  |  |  |  |  |  |
|  | Cons | 3.810744 | 5.194531 | 0.73 | 0.463 | -6.370349 | 13.99184 |
| CER VS TRE |  |  |  |  |  |  |  |
|  | Age | -0.7423954 | 1.208102 | -0.61 | 0.539 | -3.110232 | 1.625441 |
|  | Cons | 40.86928 | 67.19678 | 0.61 | 0.543 | -90.83399 | 172.5725 |
| CER VS GD |  |  |  |  |  |  |  |
|  | Country | -0.8948875 | 0.6814726 | -1.31 | 0.189 | -2.230549 | 0.4407744 |
|  | Cons | 6.170731 | 2.851061 | 2.16 | 0.03 | 0.5827533 | 11.75871 |
| CER VS STF |  |  |  |  |  |  |  |
|  | Cons | 4.39103 | 5.172232 | 0.85 | 0.396 | -5.746357 | 14.52842 |
| CER VS TRE |  |  |  |  |  |  |  |
|  | Country | -0.9961313 | 0.7004471 | -1.42 | 0.155 | -2.368982 | 0.3767198 |
|  | Cons | 3.048555 | 3.049734 | 1 | 0.317 | -2.928815 | 9.025924 |

Supplementary Table28.Meta-regression analysis of TC outcomes in overweight/obese patients with type 2 diabetes, with HbA1c, BMI, duration of intervention, age and country as moderator variables

| **Intervention** | **Covariate** | **Coefficient** | **Standard Error** | **Z-statistic** | **P>z** | **lower confidence interval** | **upper confidence interval** |
| --- | --- | --- | --- | --- | --- | --- | --- |
| CER VS GD |  |  |  |  |  |  |  |
|  | HbA1c | 0.1766893 | 0.292259 | 0.6 | 0.545 | -0.3961278 | 0.7495063 |
|  | Cons | -1.274148 | 2.164546 | -0.59 | 0.556 | -5.51658 | 2.968284 |
| CER VS STF |  |  |  |  |  |  |  |
|  | HbA1c | 0.3623499 | 0.5108912 | 0.71 | 0.478 | -0.6389784 | 1.363678 |
|  | Cons | -2.315155 | 3.899548 | -0.59 | 0.553 | -9.958128 | 5.327819 |
| CER VS TRE |  |  |  |  |  |  |  |
|  | HbA1c | -0.3509753 | 0.6959917 | -0.5 | 0.614 | -1.715094 | 1.013143 |
|  | Cons | 3.046275 | 5.654223 | 0.54 | 0.59 | -8.035798 | 14.12835 |
| CER VS GD |  |  |  |  |  |  |  |
|  | BMI | 0.025561 | 0.0429418 | 0.6 | 0.552 | -0.0586033 | 0.1097253 |
|  | Cons | -0.8402076 | 1.471302 | -0.57 | 0.568 | -3.723907 | 2.043492 |
| CER VS STF |  |  |  |  |  |  |  |
|  | BMI | 0.3826625 | 0.6434555 | 0.59 | 0.552 | -0.8784871 | 1.643812 |
|  | Cons | -13.44585 | 23.35007 | -0.58 | 0.565 | -59.21115 | 32.31945 |
| CER VS TRE |  |  |  |  |  |  |  |
|  | BMI | 0.0425414 | 0.0385002 | 1.1 | 0.269 | -0.0329176 | 0.1180004 |
|  | Cons | -1.455938 | 1.348242 | -1.08 | 0.28 | -4.098443 | 1.186568 |
| CER VS GD |  |  |  |  |  |  |  |
|  | Duration of intervention | -0.0024743 | 0.0040791 | -0.61 | 0.544 | -0.0104692 | 0.0055205 |
|  | Cons | 0.2176376 | 0.3340177 | 0.65 | 0.515 | -0.437025 | 0.8723002 |
| CER VS STF |  |  |  |  |  |  |  |
|  | Duration of intervention | -0.0099423 | 0.0140747 | -0.71 | 0.48 | -0.0375282 | 0.0176436 |
|  | Cons | 0.8072304 | 0.5637543 | 1.43 | 0.152 | -0.2977078 | 1.912169 |
| CER VS TRE |  |  |  |  |  |  |  |
|  | Duration of intervention | 0.0155082 | 0.0197876 | 0.78 | 0.433 | -0.0232747 | 0.0542912 |
|  | Cons | -0.1681551 | 0.480191 | -0.35 | 0.726 | -1.109312 | 0.773002 |
| CER VS GD |  |  |  |  |  |  |  |
|  | Age | -0.0195755 | 0.0325727 | -0.6 | 0.548 | -0.0834167 | 0.0442657 |
|  | Cons | 1.231709 | 2.000793 | 0.62 | 0.538 | -2.689773 | 5.153191 |
| CER VS STF |  |  |  |  |  |  |  |
|  | Age | -0.2082918 | 0.399027 | -0.52 | 0.602 | -0.9903704 | 0.5737867 |
|  | Cons | 13.0358 | 24.15217 | 0.54 | 0.589 | -34.30158 | 60.37318 |
| CER VS TRE |  |  |  |  |  |  |  |
|  | Age | 0.0138456 | 0.0551517 | 0.25 | 0.802 | -0.0942498 | 0.121941 |
|  | Cons | -0.5592194 | 3.059547 | -0.18 | 0.855 | -6.555822 | 5.437383 |
| CER VS GD |  |  |  |  |  |  |  |
|  | Country | -0.0582538 | 0.0968031 | -0.6 | 0.547 | -0.2479843 | 0.1314767 |
|  | Cons | 0.330245 | 0.5133841 | 0.64 | 0.52 | -0.6759692 | 1.336459 |
| CER VS STF |  |  |  |  |  |  |  |
|  | Country | 0.133606 | 0.2249315 | 0.59 | 0.553 | -0.3072517 | 0.5744637 |
|  | Cons | 0.062788 | 0.6721978 | 0.09 | 0.926 | -1.254695 | 1.380272 |
| CER VS TRE |  |  |  |  |  |  |  |
|  | Country | 0.0502683 | 0.1717041 | 0.29 | 0.77 | -0.2862655 | 0.3868022 |
|  | Cons | 0.0517198 | 0.5736454 | 0.09 | 0.928 | -1.072604 | 1.176044 |

Supplementary Table29.Sensitivity analysis for the HbA1c outcome in overweight/obese patients with type 2 diabetes

| **dropped_id** | **comparison** | **eff** | **lci** | **uci** | **connected** |
| --- | --- | --- | --- | --- | --- |
| S Carter 2016 | GD VS CER | -0.55146373 | -0.9111096 | -0.1918179 | 1 |
| S Carter 2016 | GD VS STF | -0.20311502 | -0.8985105 | 0.4922805 | 1 |
| S Carter 2016 | GD VS TRE | -0.55185868 | -0.7769339 | -0.3267834 | 1 |
| B T Corley 2018 | GD VS CER | -0.58463142 | -0.9500693 | -0.2191935 | 1 |
| B T Corley 2018 | GD VS STF | -0.38460292 | -1.007946 | 0.2387398 | 1 |
| B T Corley 2018 | GD VS TRE | -0.56213395 | -0.7837741 | -0.3404938 | 1 |
| S Carter 2019 | GD VS CER | -0.54823242 | -0.906624 | -0.1898409 | 1 |
| S Carter 2019 | GD VS STF | -0.23327007 | -0.8692873 | 0.4027472 | 1 |
| S Carter 2019 | GD VS TRE | -0.55086884 | -0.7757784 | -0.3259593 | 1 |
| Tingting Che 2021 | GD VS CER | -0.41824528 | -0.6495361 | -0.1869545 | 1 |
| Tingting Che 2021 | GD VS STF | -0.17805573 | -0.6010067 | 0.2448953 | 1 |
| Tingting Che 2021 | GD VS TRE | -0.37736338 | -0.5042447 | -0.250482 | 1 |
| Vasiliki Pavlou 2023 | GD VS CER | -0.45546577 | -0.8790182 | -0.0319134 | 1 |
| Vasiliki Pavlou 2023 | GD VS STF | -0.19489246 | -0.784795 | 0.3950101 | 1 |
| Vasiliki Pavlou 2023 | GD VS TRE | -0.49786866 | -0.7461252 | -0.2496122 | 1 |
| Evelyn B Parr 2024 | GD VS CER | -0.54222786 | -0.9313772 | -0.1530785 | 1 |
| Evelyn B Parr 2024 | GD VS STF | -0.26736836 | -0.8379511 | 0.3032144 | 1 |
| Evelyn B Parr 2024 | GD VS TRE | -0.54955804 | -0.7775685 | -0.3215475 | 1 |
| Naparat Sukkriang 2024 | GD VS CER | -0.60133881 | -0.9560941 | -0.2465835 | 1 |
| Naparat Sukkriang 2024 | GD VS STF | -0.31990323 | -0.8629625 | 0.223156 | 1 |
| Naparat Sukkriang 2024 | GD VS TRE | -0.61833348 | -0.856849 | -0.379818 | 1 |
| Domenico Tricò 2024 | GD VS CER | -0.48999111 | -0.9069924 | -0.0729898 | 1 |
| Domenico Tricò 2024 | GD VS STF | -0.22392591 | -0.8073437 | 0.3594919 | 1 |
| Domenico Tricò 2024 | GD VS TRE | -0.55663115 | -0.7857332 | -0.3275291 | 1 |
| Caroline Kaercher Kramer 2025 | GD VS CER | -0.59805579 | -0.9549674 | -0.2411442 | 1 |
| Caroline Kaercher Kramer 2025 | GD VS STF | -0.31673435 | -0.8622584 | 0.2287898 | 1 |
| Caroline Kaercher Kramer 2025 | GD VS TRE | -0.61491945 | -0.8556356 | -0.3742033 | 1 |
| Maarya Mohammed Siddiqi 2025 | GD VS CER | -0.53652644 | -0.9060304 | -0.1670225 | 1 |
| Maarya Mohammed Siddiqi 2025 | GD VS STF | -0.26221787 | -0.8254351 | 0.3009993 | 1 |
| Maarya Mohammed Siddiqi 2025 | GD VS TRE | -0.54072682 | -0.7886423 | -0.2928114 | 1 |
| Mongkontida Umphonsathien 2025 | GD VS CER | -0.61240424 | -0.9861032 | -0.2387053 | 1 |
| Mongkontida Umphonsathien 2025 | GD VS STF | -0.32742483 | -0.883689 | 0.2288394 | 1 |
| Mongkontida Umphonsathien 2025 | GD VS TRE | -0.570589 | -0.7923567 | -0.3488213 | 1 |

Supplementary Table30.Sensitivity analysis for the FBG outcome in overweight/obese patients with type 2 diabetes

| **dropped_id** | **comparison** | **eff** | **lci** | **uci** | **connected** |
| --- | --- | --- | --- | --- | --- |
| S Carter 2019 | GD VS CER | -0.70944181 | -1.531075 | 0.1121912 | 1 |
| S Carter 2019 | GD VS STF | -0.85383687 | -1.23939 | -0.4682842 | 1 |
| Tingting Che 2021 | GD VS CER | -0.77946179 | -1.702042 | 0.1431179 | 1 |
| Tingting Che 2021 | GD VS STF | -0.47944427 | -2.142345 | 1.183457 | 1 |
| Tingting Che 2021 | GD VS TRE | -0.93790905 | -1.45862 | -0.417198 | 1 |
| Charlotte Andriessen 2022 | GD VS CER | -0.70259213 | -1.548594 | 0.1434096 | 1 |
| Charlotte Andriessen 2022 | GD VS STF | -0.4025779 | -1.982951 | 1.177796 | 1 |
| Charlotte Andriessen 2022 | GD VS TRE | -0.84776693 | -1.260548 | -0.4349864 | 1 |
| Evelyn B Parr 2024 | GD VS CER | -0.68155684 | -1.732037 | 0.3689229 | 1 |
| Evelyn B Parr 2024 | GD VS STF | -0.38154277 | -2.094459 | 1.331373 | 1 |
| Evelyn B Parr 2024 | GD VS TRE | -0.85992085 | -1.274838 | -0.4450034 | 1 |
| Naparat Sukkriang 2024 | GD VS CER | -0.64727702 | -1.519111 | 0.2245574 | 1 |
| Naparat Sukkriang 2024 | GD VS STF | -0.34726401 | -1.950947 | 1.256418 | 1 |
| Naparat Sukkriang 2024 | GD VS TRE | -0.7866159 | -1.246711 | -0.3265206 | 1 |
| Domenico Tricò 2024 | GD VS CER | -0.66517016 | -1.808777 | 0.4784368 | 1 |
| Domenico Tricò 2024 | GD VS STF | -0.3651565 | -2.137376 | 1.407063 | 1 |
| Domenico Tricò 2024 | GD VS TRE | -0.86055563 | -1.276401 | -0.4447106 | 1 |
| Caroline Kaercher Kramer 2025 | GD VS CER | -0.82726399 | -1.632186 | -0.0223422 | 1 |
| Caroline Kaercher Kramer 2025 | GD VS STF | -0.52724814 | -2.035128 | 0.9806319 | 1 |
| Caroline Kaercher Kramer 2025 | GD VS TRE | -0.98134867 | -1.39355 | -0.5691471 | 1 |
| Maarya Mohammed Siddiqi 2025 | GD VS CER | -0.58012976 | -1.252553 | 0.0922938 | 1 |
| Maarya Mohammed Siddiqi 2025 | GD VS STF | -0.28012141 | -1.632262 | 1.07202 | 1 |
| Maarya Mohammed Siddiqi 2025 | GD VS TRE | -0.70269324 | -0.9384859 | -0.4669006 | 1 |
| Mongkontida Umphonsathien 2025 | GD VS CER | -0.76698714 | -1.662342 | 0.1283676 | 1 |
| Mongkontida Umphonsathien 2025 | GD VS STF | -0.46697142 | -2.06809 | 1.134148 | 1 |
| Mongkontida Umphonsathien 2025 | GD VS TRE | -0.86707964 | -1.270327 | -0.4638321 | 1 |

Supplementary Table31.Sensitivity analysis for the Weight outcome in overweight/obese patients with type 2 diabetes

| **dropped_id** | **comparison** | **eff** | **lci** | **uci** | **connected** |
| --- | --- | --- | --- | --- | --- |
| B T Corley 2018 | GD VS CER | -1.7068259 | -2.910916 | -0.5027359 | 1 |
| B T Corley 2018 | GD VS STF | -1.7067906 | -5.93642 | 2.522839 | 1 |
| B T Corley 2018 | GD VS TRE | -2.3194295 | -2.995084 | -1.643775 | 1 |
| S Carter 2019 | GD VS CER | -1.7068328 | -2.910926 | -0.5027395 | 1 |
| S Carter 2019 | GD VS STF | 0.5 | -12.56461 | 13.56461 | 1 |
| S Carter 2019 | GD VS TRE | -2.3194308 | -2.995087 | -1.643775 | 1 |
| Tingting Che 2021 | GD VS CER | -1.7305631 | -3.048845 | -0.412281 | 1 |
| Tingting Che 2021 | GD VS STF | -1.5250455 | -5.677866 | 2.627774 | 1 |
| Tingting Che 2021 | GD VS TRE | -2.4092067 | -3.331272 | -1.487141 | 1 |
| Charlotte Andriessen 2022 | GD VS CER | -1.7621487 | -2.838946 | -0.6853514 | 1 |
| Charlotte Andriessen 2022 | GD VS STF | -1.5754518 | -5.442791 | 2.291887 | 1 |
| Charlotte Andriessen 2022 | GD VS TRE | -2.4241613 | -3.027457 | -1.820866 | 1 |
| Piero Ruggenenti 2022 | GD VS CER | -1.7097091 | -2.956557 | -0.462861 | 1 |
| Piero Ruggenenti 2022 | GD VS STF | -1.5148076 | -5.558296 | 2.528681 | 1 |
| Piero Ruggenenti 2022 | GD VS TRE | -2.3222801 | -3.011314 | -1.633246 | 1 |
| Vasiliki Pavlou 2023 | GD VS CER | -1.8984082 | -3.360156 | -0.4366606 | 1 |
| Vasiliki Pavlou 2023 | GD VS STF | -1.6846059 | -5.809454 | 2.440243 | 1 |
| Vasiliki Pavlou 2023 | GD VS TRE | -2.2696254 | -3.000031 | -1.53922 | 1 |
| Evelyn B Parr 2024 | GD VS CER | -1.6881759 | -2.894595 | -0.481757 | 1 |
| Evelyn B Parr 2024 | GD VS STF | -1.4958582 | -5.521966 | 2.53025 | 1 |
| Evelyn B Parr 2024 | GD VS TRE | -2.3171404 | -2.992933 | -1.641348 | 1 |
| Naparat Sukkriang 2024 | GD VS CER | -1.6812059 | -2.994925 | -0.367487 | 1 |
| Naparat Sukkriang 2024 | GD VS STF | -1.4806614 | -5.627488 | 2.666165 | 1 |
| Naparat Sukkriang 2024 | GD VS TRE | -2.3135516 | -3.232531 | -1.394572 | 1 |
| Domenico Tricò 2024 | GD VS CER | -1.3757213 | -2.745089 | -0.0063532 | 1 |
| Domenico Tricò 2024 | GD VS STF | -1.210383 | -5.285024 | 2.864258 | 1 |
| Domenico Tricò 2024 | GD VS TRE | -2.3744979 | -3.081501 | -1.667495 | 1 |
| Caroline Kaercher Kramer 2025 | GD VS CER | -1.6001089 | -2.71178 | -0.4884378 | 1 |
| Caroline Kaercher Kramer 2025 | GD VS STF | -1.4233969 | -5.339058 | 2.492265 | 1 |
| Caroline Kaercher Kramer 2025 | GD VS TRE | -2.1306148 | -2.685295 | -1.575935 | 1 |
| Maarya Mohammed Siddiqi 2025 | GD VS CER | -1.5672216 | -2.840194 | -0.2942497 | 1 |
| Maarya Mohammed Siddiqi 2025 | GD VS STF | -1.3848324 | -5.436235 | 2.66657 | 1 |
| Maarya Mohammed Siddiqi 2025 | GD VS TRE | -2.299029 | -2.984823 | -1.613235 | 1 |

Supplementary Table32.Sensitivity analysis for the BMI outcome in overweight/obese patients with type 2 diabetes

| **dropped_id** | **comparison** | **eff** | **lci** | **uci** | **connected** |
| --- | --- | --- | --- | --- | --- |
| B T Corley 2018 | GD VS CER | -0.59024282 | -1.102764 | -0.0777215 | 1 |
| B T Corley 2018 | GD VS STF | -0.49023128 | -1.904793 | 0.9243305 | 1 |
| B T Corley 2018 | GD VS CER | -1.3190564 | -1.715284 | -0.9228292 | 1 |
| S Carter 2019 | GD VS STF | -0.59024617 | -1.102769 | -0.0777232 | 1 |
| S Carter 2019 | GD VS TRE | 0.30000001 | -3.119151 | 3.719151 | 1 |
| S Carter 2019 | GD VS CER | -1.3190569 | -1.715284 | -0.9228297 | 1 |
| Piero Ruggenenti 2022 | GD VS STF | -0.57061444 | -1.121826 | -0.019403 | 1 |
| Piero Ruggenenti 2022 | GD VS TRE | -0.37085187 | -1.691289 | 0.9495856 | 1 |
| Piero Ruggenenti 2022 | GD VS CER | -1.3162744 | -1.713544 | -0.9190053 | 1 |
| Vasiliki Pavlou 2023 | GD VS STF | -0.70446738 | -1.502424 | 0.0934891 | 1 |
| Vasiliki Pavlou 2023 | GD VS TRE | -0.48737566 | -1.900117 | 0.9253656 | 1 |
| Vasiliki Pavlou 2023 | GD VS CER | -1.2890862 | -1.72492 | -0.8532523 | 1 |
| Evelyn B Parr 2024 | GD VS STF | -0.55672507 | -1.071634 | -0.0418159 | 1 |
| Evelyn B Parr 2024 | GD VS TRE | -0.35876068 | -1.668045 | 0.9505234 | 1 |
| Evelyn B Parr 2024 | GD VS CER | -1.3269036 | -1.725796 | -0.9280109 | 1 |
| Caroline Kaercher Kramer 2025 | GD VS CER | -0.58458332 | -1.111991 | -0.0571757 | 1 |
| Caroline Kaercher Kramer 2025 | GD VS STF | -0.38301228 | -1.696061 | 0.9300367 | 1 |
| Caroline Kaercher Kramer 2025 | GD VS TRE | -1.3577296 | -2.089647 | -0.6258124 | 1 |
| Maarya Mohammed Siddiqi 2025 | GD VS CER | -0.57551036 | -1.084002 | -0.0670191 | 1 |
| Maarya Mohammed Siddiqi 2025 | GD VS STF | -0.37511394 | -1.682496 | 0.932268 | 1 |
| Maarya Mohammed Siddiqi 2025 | GD VS TRE | -1.3187471 | -1.735836 | -0.9016578 | 1 |
| Mongkontida Umphonsathien 2025 | GD VS CER | -0.519934 | -1.086335 | 0.0464669 | 1 |
| Mongkontida Umphonsathien 2025 | GD VS STF | -0.32673274 | -1.652033 | 0.9985672 | 1 |
| Mongkontida Umphonsathien 2025 | GD VS TRE | -1.3090912 | -1.706789 | -0.9113931 | 1 |

Supplementary Table33.Sensitivity analysis for the WC outcome in overweight/obese patients with type 2 diabetes

| **dropped_id** | **comparison** | **eff** | **lci** | **uci** | **connected** |
| --- | --- | --- | --- | --- | --- |
| B T Corley 2018 | GD VS CER | -2.725486 | -4.562257 | -0.8887147 | 1 |
| B T Corley 2018 | GD VS STF | -3.6852447 | -5.074908 | -2.295581 | 1 |
| Piero Ruggenenti 2022 | GD VS STF | -3.5761769 | -5.781684 | -1.37067 | 1 |
| Piero Ruggenenti 2022 | GD VS TRE | 1.8000001 | -8.165004 | 11.765 | 1 |
| Piero Ruggenenti 2022 | GD VS CER | -3.773292 | -5.168689 | -2.377895 | 1 |
| Vasiliki Pavlou 2023 | GD VS STF | -0.80000019 | -4.118125 | 2.518125 | 1 |
| Vasiliki Pavlou 2023 | GD VS TRE | 1.8000001 | -8.165004 | 11.765 | 1 |
| Vasiliki Pavlou 2023 | GD VS CER | -3.993424 | -5.791526 | -2.195322 | 1 |
| Caroline Kaercher Kramer 2025 | disconnected | | | | 0 |
| Maarya Mohammed Siddiqi 2025 | disconnected | | | | 0 |

Supplementary Table34.Sensitivity analysis for the TC outcome in overweight/obese patients with type 2 diabetes

| **dropped_id** | **comparison** | **eff** | **lci** | **uci** | **connected** |
| --- | --- | --- | --- | --- | --- |
| B T Corley 2018 | disconnected | | | | 0 |
| S Carter 2019 | disconnected | | | | 0 |
| Tingting Che 2021 | disconnected | | | | 0 |
| Piero Ruggenenti 2022 | disconnected | | | | 0 |
| Vasiliki Pavlou 2023 | disconnected | | | | 0 |


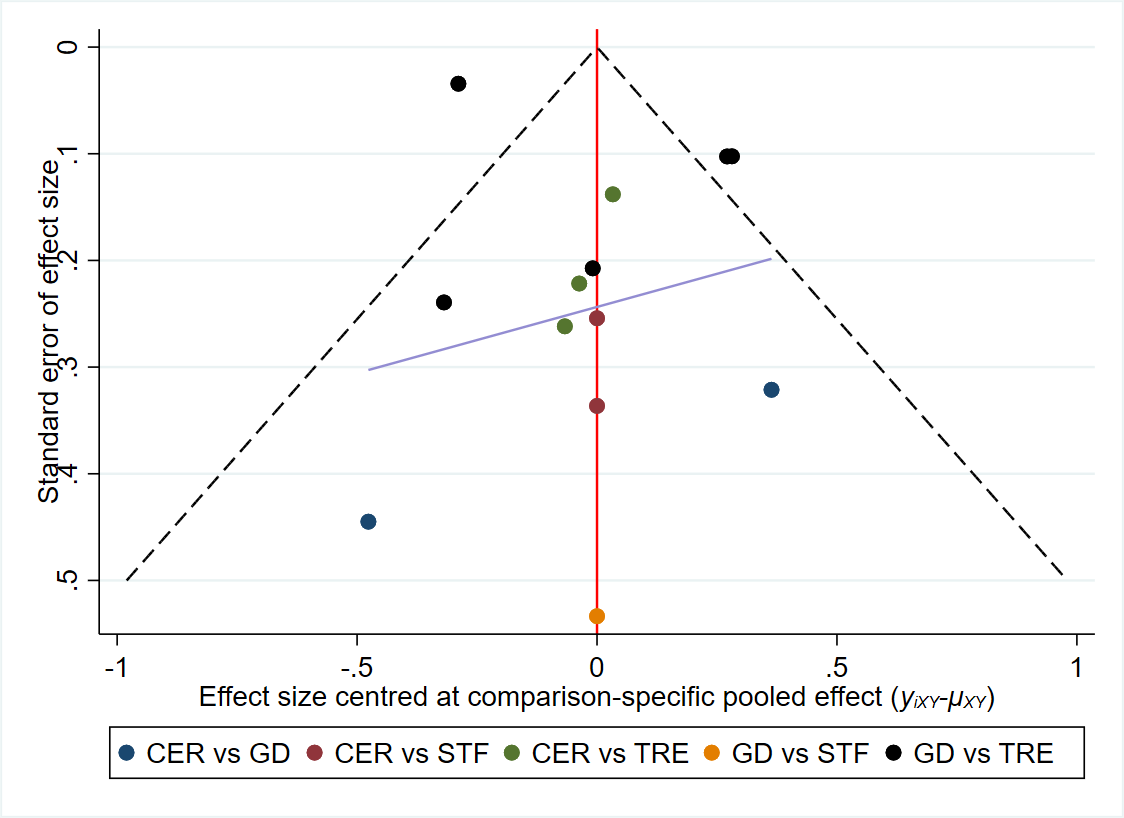


Supplementary Figure 12.Comparison-adjusted funnel plot for the HbA1c outcome in overweight/obese patients with type 2 diabetes


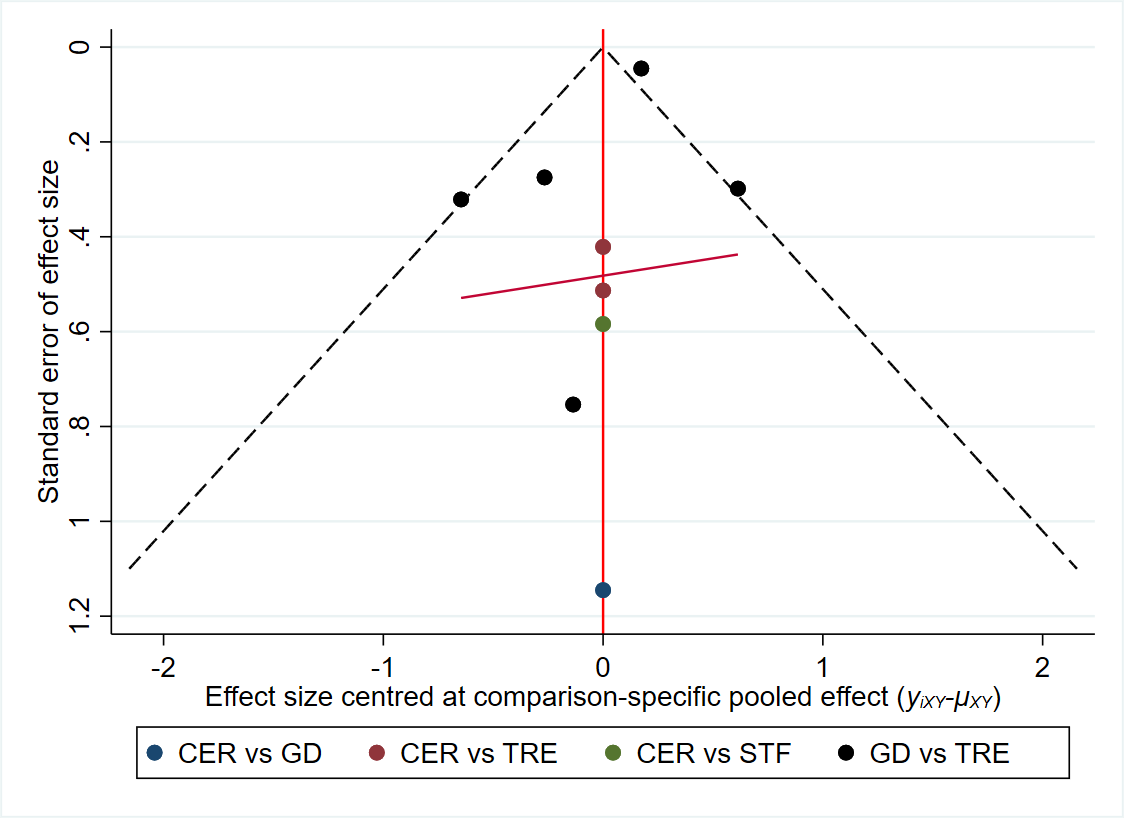
Supplementary Figure 13.Comparison-adjusted funnel plot for the FBG outcome in overweight/obese patients with type 2 diabetes


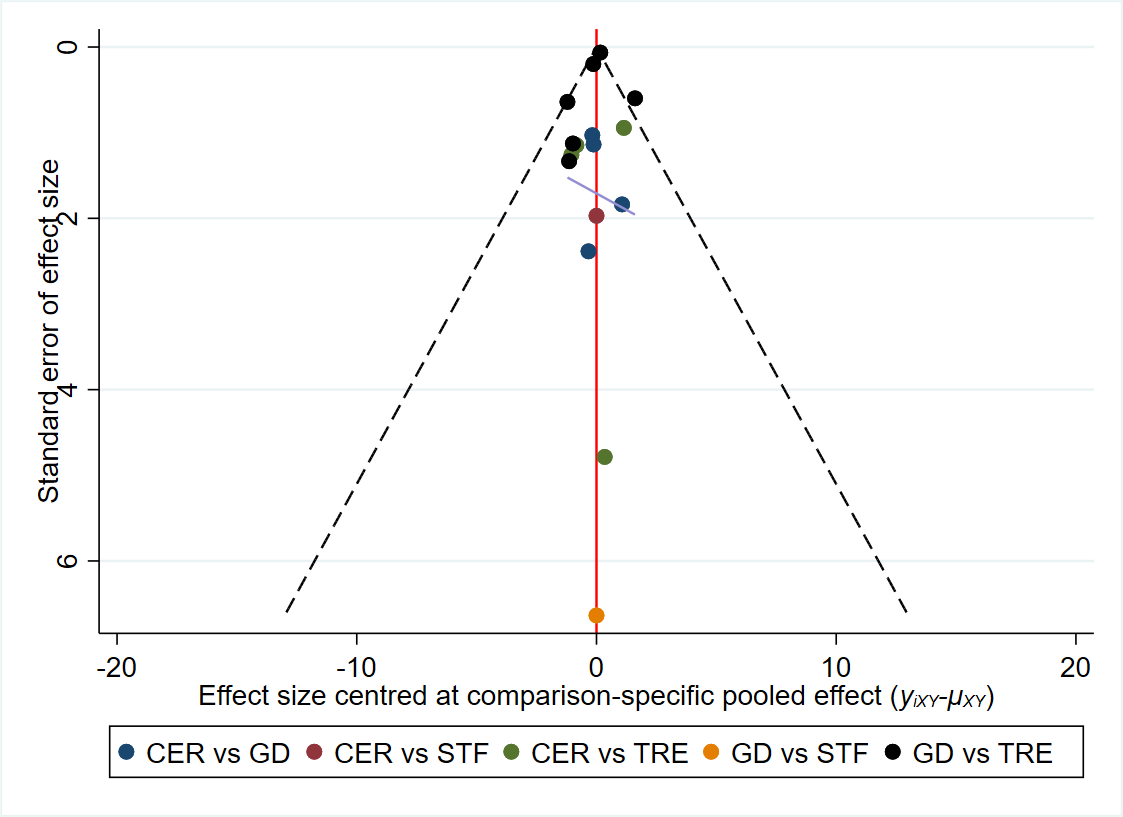


Supplementary Figure 14.Comparison-adjusted funnel plot for the Weight outcome in overweight/obese patients with type 2 diabetes


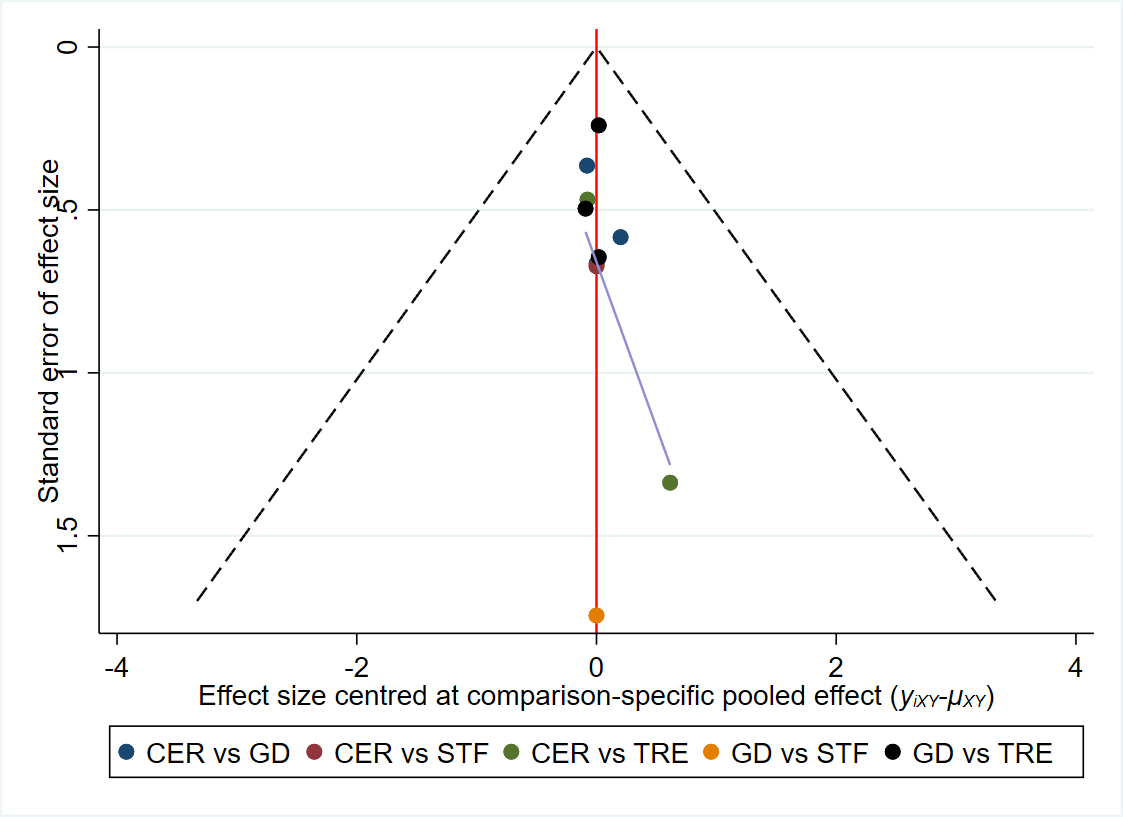


Supplementary Figure 15.Comparison-adjusted funnel plot for the BMI outcome in overweight/obese patients with type 2 diabetes


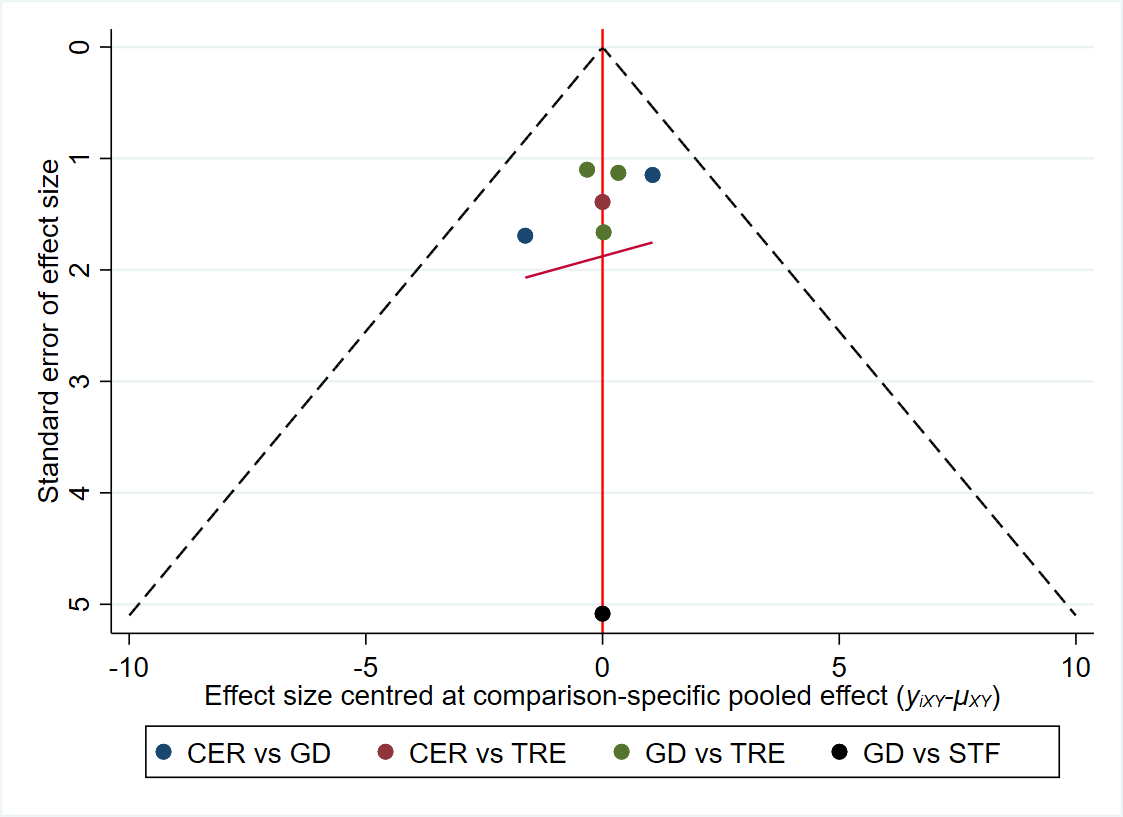


Supplementary Figure 16.Comparison-adjusted funnel plot for the WC outcome in overweight/obese patients with type 2 diabetes


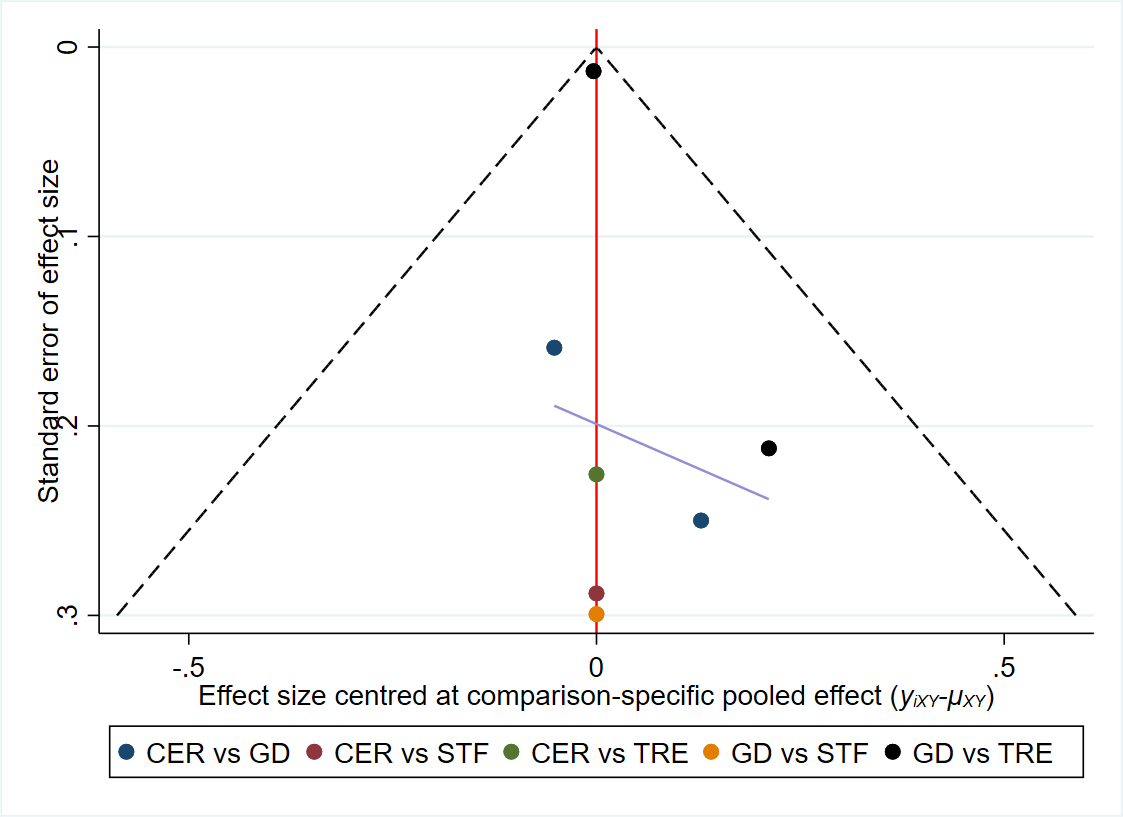


Supplementary Figure 17.Comparison-adjusted funnel plot for the TC outcome in overweight/obese patients with type 2 diabetes

Supplementary Table35.GRADE certainty of evidence for the HbA1c outcome in overweight/obese patients with type 2 diabetes

| **Comparison** | **Number of studies** | **Within-study bias** | **Reporting bias** | **Indirectness** | **Imprecision** | **Heterogeneity** | **Incoherence** | **Confidence rating** | **Reason**  **(s) for downgrading** |
| --- | --- | --- | --- | --- | --- | --- | --- | --- | --- |
| CER:GD | 2 | Some concerns | Low risk | No concerns | No concerns | Some concerns | No concerns | Low | ["Within-study bias","Heterogeneity"] |
| CER:STF | 2 | Some concerns | Low risk | No concerns | Some concerns | Some concerns | No concerns | Very low | ["Within-study bias","Imprecision","Heterogeneity"] |
| CER:TRE | 3 | Some concerns | Low risk | No concerns | No concerns | Major concerns | No concerns | Very low | ["Within-study bias","Heterogeneity"] |
| GD:STF | 1 | Some concerns | Low risk | No concerns | Some concerns | Some concerns | No concerns | Very low | ["Imprecision","Heterogeneity"] |
| GD:TRE | 5 | Some concerns | Low risk | No concerns | No concerns | Some concerns | No concerns | Low | ["Within-study bias","Heterogeneity"] |
| STF:TRE | 0 | Some concerns | Low risk | No concerns | Some concerns | Some concerns | No concerns | Very low | ["Within-study bias","Imprecision","Heterogeneity"] |

Supplementary Table36.GRADE certainty of evidence for the FBG outcome in overweight/obese patients with type 2 diabetes

| **Comparison** | **Number of studies** | **Within-study bias** | **Reporting bias** | **Indirectness** | **Imprecision** | **Heterogeneity** | **Incoherence** | **Confidence rating** | **Reason**  **(s) for downgrading** |
| --- | --- | --- | --- | --- | --- | --- | --- | --- | --- |
| CER:GD | 1 | Some concerns | Low risk | No concerns | Some concerns | Some concerns | No concerns | Very low | ["Within-study bias","Imprecision","Heterogeneity"] |
| CER:STF | 1 | Some concerns | Low risk | No concerns | Major concerns | No concerns | No concerns | Very low | ["Within-study bias","Imprecision"] |
| CER:TRE | 2 | Some concerns | Low risk | No concerns | Major concerns | No concerns | No concerns | Very low | ["Within-study bias","Imprecision"] |
| GD:TRE | 5 | Some concerns | Low risk | No concerns | No concerns | No concerns | No concerns | Moderate | ["Within-study bias"] |
| GD:STF | 0 | Some concerns | Low risk | No concerns | Major concerns | No concerns | No concerns | Very low | ["Within-study bias","Imprecision"] |
| STF:TRE | 0 | Some concerns | Low risk | No concerns | Major concerns | No concerns | No concerns | Very low | ["Within-study bias","Imprecision"] |

Supplementary Table37.GRADE certainty of evidence for the Weight outcome in overweight/obese patients with type 2 diabetes

| **Comparison** | **Number of studies** | **Within-study bias** | **Reporting bias** | **Indirectness** | **Imprecision** | **Heterogeneity** | **Incoherence** | **Confidence rating** | **Reason**  **(s) for downgrading** |
| --- | --- | --- | --- | --- | --- | --- | --- | --- | --- |
| CER:GD | 4 | Some concerns | Low risk | No concerns | No concerns | No concerns | No concerns | Moderate | ["Within-study bias"] |
| CER:STF | 1 | Some concerns | Low risk | No concerns | Major concerns | No concerns | No concerns | Very low | ["Within-study bias","Imprecision"] |
| CER:TRE | 4 | Some concerns | Low risk | No concerns | No concerns | No concerns | No concerns | Moderate | ["Within-study bias"] |
| GD:STF | 1 | Some concerns | Low risk | No concerns | Major concerns | No concerns | No concerns | Very low | ["Within-study bias","Imprecision"] |
| GD:TRE | 6 | No concerns | Low risk | No concerns | No concerns | No concerns | No concerns | High | [] |
| STF:TRE | 0 | Some concerns | Low risk | No concerns | Major concerns | No concerns | No concerns | Very low | ["Within-study bias","Imprecision"] |

Supplementary Table38.GRADE certainty of evidence for the BMI outcome in overweight/obese patients with type 2 diabetes

| **Comparison** | **Number of studies** | **Within-study bias** | **Reporting bias** | **Indirectness** | **Imprecision** | **Heterogeneity** | **Incoherence** | **Confidence rating** | **Reason**  **(s) for downgrading** |
| --- | --- | --- | --- | --- | --- | --- | --- | --- | --- |
| CER:GD | 3 | No concerns | Low risk | No concerns | No concerns | Some concerns | No concerns | Moderate | ["Heterogeneity"] |
| CER:STF | 1 | Some concerns | Low risk | No concerns | Major concerns | No concerns | No concerns | Very low | ["Within-study bias","Imprecision"] |
| CER:TRE | 2 | No concerns | Low risk | No concerns | No concerns | Some concerns | No concerns | Moderate | ["Heterogeneity"] |
| GD:STF | 1 | Some concerns | Low risk | No concerns | Some concerns | Some concerns | No concerns | Very low | ["Within-study bias","Imprecision","Heterogeneity"] |
| GD:TRE | 3 | Some concerns | Low risk | No concerns | No concerns | No concerns | No concerns | Moderate | ["Within-study bias"] |
| STF:TRE | 0 | Some concerns | Low risk | No concerns | Some concerns | No concerns | No concerns | Low | ["Within-study bias","Imprecision"] |

Supplementary Table39.GRADE certainty of evidence for the WC outcome in overweight/obese patients with type 2 diabetes

| **Comparison** | **Number of studies** | **Within-study bias** | **Reporting bias** | **Indirectness** | **Imprecision** | **Heterogeneity** | **Incoherence** | **Confidence rating** | **Reason**  **(s) for downgrading** |
| --- | --- | --- | --- | --- | --- | --- | --- | --- | --- |
| CER:GD | 2 | No concerns | Low risk | No concerns | No concerns | Some concerns | No concerns | Moderate | ["Heterogeneity"] |
| CER:TRE | 1 | No concerns | Low risk | No concerns | Some concerns | Some concerns | No concerns | Low | ["Imprecision","Heterogeneity"] |
| GD:STF | 1 | Some concerns | Low risk | No concerns | Major concerns | No concerns | No concerns | Very low | ["Within-study bias","Imprecision"] |
| GD:TRE | 3 | Some concerns | Low risk | No concerns | No concerns | No concerns | No concerns | Moderate | ["Within-study bias"] |
| CER:STF | 0 | Some concerns | Low risk | No concerns | Major concerns | No concerns | No concerns | Very low | ["Within-study bias","Imprecision"] |
| STF:TRE | 0 | Some concerns | Low risk | No concerns | Major concerns | No concerns | No concerns | Very low | ["Within-study bias","Imprecision"] |

Supplementary Table40.GRADE certainty of evidence for the TC outcome in overweight/obese patients with type 2 diabetes

| **Comparison** | **Number of studies** | **Within-study bias** | **Reporting bias** | **Indirectness** | **Imprecision** | **Heterogeneity** | **Incoherence** | **Confidence rating** | **Reason**  **(s) for downgrading** |
| --- | --- | --- | --- | --- | --- | --- | --- | --- | --- |
| CER:GD | 2 | No concerns | Low risk | No concerns | No concerns | Major concerns | No concerns | Low | ["Heterogeneity"] |
| CER:STF | 1 | Some concerns | Low risk | No concerns | No concerns | Major concerns | No concerns | Very low | ["Within-study bias","Heterogeneity"] |
| CER:TRE | 1 | No concerns | Low risk | No concerns | No concerns | Major concerns | No concerns | Low | ["Heterogeneity"] |
| GD:STF | 1 | Some concerns | Low risk | No concerns | Some concerns | Some concerns | No concerns | Very low | ["Within-study bias","Imprecision","Heterogeneity"] |
| GD:TRE | 2 | No concerns | Low risk | No concerns | No concerns | No concerns | No concerns | High | [] |
| STF:TRE | 0 | No concerns | Low risk | No concerns | No concerns | Major concerns | No concerns | Low | ["Heterogeneity"] |
